# Azaindolo[3,2,1‐*jk*]carbazoles: New Building Blocks for Functional Organic Materials

**DOI:** 10.1002/chem.201805578

**Published:** 2019-02-27

**Authors:** Thomas Kader, Berthold Stöger, Johannes Fröhlich, Paul Kautny

**Affiliations:** ^1^ Institute of Applied Synthetic Chemistry TU Wien Getreidemarkt 9/163 1060 Vienna Austria; ^2^ X-Ray Center TU Wien Getreidemarkt 9 1060 Vienna Austria

**Keywords:** C−H activation, carbazole, functional organic materials, heterocycles, synthetic methods

## Abstract

The preparation and characterization of 12 azaindolo[3,2,1‐*jk*]carbazoles is presented. Ring‐closing C−H activation allowed for the convenient preparation of six singly and six doubly nitrogen‐substituted indolo[3,2,1‐*jk*]carbazole derivatives in which ten of the materials have not been described in the literature before. The detailed photophysical and electrochemical characterization of the developed materials revealed a significant impact of the incorporation of pyridine‐like nitrogen into the fully planar indolo[3,2,1‐*jk*]carbazole backbone. Furthermore, the nitrogen position decisively impacted intermolecular hydrogen bonding and thus the solid‐state alignment. Ultimately, the versatility of the azaindolo[3,2,1‐*jk*]carbazoles scaffold makes this class of materials an attractive new building block for the design of functional organic materials.

## Introduction

The development of π‐conjugated small molecules with tailored molecular properties has been one of the major driving forces for the rapid development of the field of organic electronics over the last decades.^1^, ^2^ In particular, small to medium sized polycyclic (hetero)aromatic molecules with a defined molecular structure are of tremendous importance.^2^, ^3^, ^4^, ^5^, ^6^ Accordingly, many materials based on these building blocks have been developed for applications in organic light‐emitting diodes (OLEDs),^2^, ^7^, ^8^, ^9^, ^10^, ^11^, ^12^ organic field‐effect transistors (OFETs),^1^, ^2^, ^6^, ^7^, ^13^, ^14^, ^15^ organic photovoltaics (OPVs),^2^, ^16^, ^17^, ^18^, ^19^ and sensing technology,^11^, ^20^, ^21^, ^22^ to name a few. As a consequence, there is an ongoing quest for novel fused aromatic moieties to fulfill the requirements of the respective technological application.^2^, ^3^, ^12^

**Figure 1:**
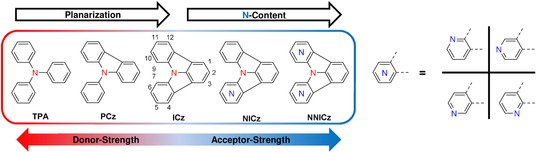
Schematic representation of the concept of planarization of the triarylamine framework and nitrogen incorporation to control donor and acceptor strength.

Recently, we introduced indolo[3,2,1‐*jk*]carbazole (ICz) as new building block for optoelectronic materials.^23^, ^24^, ^25^, ^26^ ICz can be considered a fully planarized derivative of triphenylamine (TPA). Although arylamines are widely employed as electron‐donating moieties, reports on the application of ICz have been scarce for a long time,^27^, ^28^, ^29^ owing to the elaborate preparation of the planarized scaffold (e.g., vacuum flash pyrolysis).^30^, ^31^ However, a new synthetic strategy based on modern C−H activation renders the wider application of this building block possible.^23^, ^32^ Compared with TPA and phenylcarbazole (PCz), ICz is a weaker electron donor (Figure 1). Due to the increasing planarization, the electron‐donating power of the triarylamines significantly decreases from TPA to PCz and ICz. This gradual decrease of donor strength is due to the contribution of the lone pair of the nitrogen to the aromaticity of the pyrroles formed by planarization. Therefore, the lone pair is more tightly bound to the arylamine core and less prone to be delocalized in a donor–acceptor molecule.^24^ Effectively, the ICz moiety can exhibit light acceptor properties itself as evidenced in bipolar host materials.^23^, ^33^ Furthermore, the ICz building block has been utilized as a weak electron acceptor in thermally activated delayed fluorescent (TADF) emitters.^34^ Increasing the electron‐accepting strength by substitution of the ICz scaffold with electron‐withdrawing cyano groups enabled the preparation of pure blue TADF emitters.^35^

Based on these results and the growing interest regarding the application of the indolo[3,2,1‐*jk*]carbazole as a building block for optoelectronic materials, we set out to further expand the concept of planarization to intrinsically modulate the acceptor strength by incorporation of electron‐withdrawing pyridine‐like nitrogen atoms into the ICz moiety [Figure 1, henceforth the atoms in the nitrogen‐substituted ICzs (NICzs) are numbered according to the original numbering of ICz, in which the numbers specify the positions of nitrogen atoms]. The concept of heteroatom doping is widely employed in silicon‐based semiconductor technology. Analogously, the incorporation of heteroatoms into polycyclic aromatic scaffolds has been demonstrated to effectively modify the photophysical and electrochemical properties of the parent material.^36^, ^37^ In particular, nitrogen incorporation into acenes has been successfully realized to induce n‐type charge‐transport properties in materials for OFET applications.^38^, ^39^, ^40^ Moreover, carbolines, which contain an electron‐deficient pyridine unit, were successfully employed as electron‐transporting units in host materials for efficient phosphorescent OLEDs.^41^, ^42^ Notably, the exact position of the nitrogen atom within the carboline scaffold significantly impacted the molecular properties of the materials and the device efficiency.^42^, ^43^

In addition to the electronic effects, the structure directing potential of nitrogen atoms by means of weak C−H⋅⋅⋅N hydrogen bonds can also be employed to induce intra‐44, 45 or intermolecular^46^, ^47^ interactions to control optical or electronic properties of the organic materials.^48^

Accordingly, the NICzs are an interesting class of materials. A reliable synthetic access to this scaffold would allow for the intriguing possibility to fine‐tune the molecular properties of optoelectronic materials by the subtle variation of the nitrogen position and content of the NICz building block. Notably, three NICz derivatives have already been prepared, namely **5NICz**, **7NICz**, and **7,9NICz**, which are accessible as single regioisomers by vacuum flash pyrolysis.^49^ However, all other NICzs are unknown in the literature.

In this work, we present a comprehensive synthetic approach towards all possible singly, as well as six doubly substituted NICzs employing a convenient C−H activation reaction. This strategy allowed for the control of the substitution pattern of the NICz by choice of substrate as well as the regioselectivity of the ring‐closing reaction by electronic manipulation of the nitrogen atom by oxidation. Ultimately, our approach enabled the preparation of a full set of NICz building blocks with tunable photophysical and electrochemical properties.

## Results and Discussion

### Precursor synthesis

**Scheme 1:**
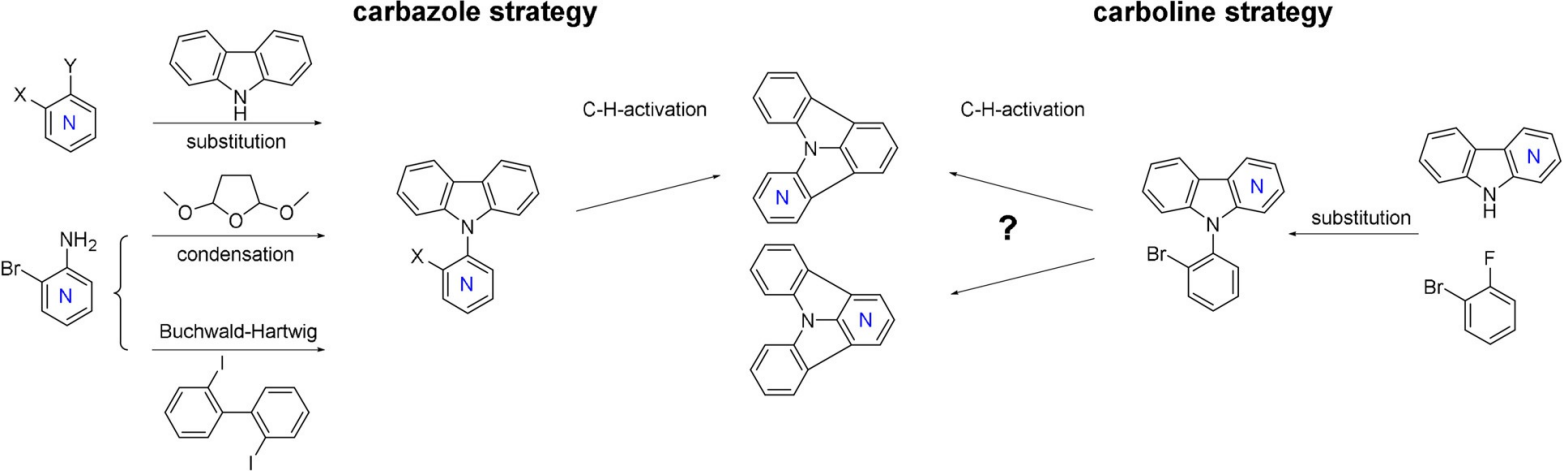
Synthetic approaches towards precursors for C−H‐activation; carbazole and carboline strategy. X and Y are either Br and Cl or Cl and F, respectively.

Compared with plain ICz, the preparation of the substrates for C−H activation towards NICz proved to be more challenging, owing to altered reactivity, stability, and availability of the required substituted pyridines. Furthermore, the issue of regioselectivity during the ring‐closing step arises as a result of the desymmetrization of the ICz scaffold by nitrogen incorporation. Therefore, we opted to investigate different approaches for the synthesis of these precursors (Scheme 1). These different strategies not only allow for the convenient synthesis of the required precursors but also pave the way for the future preparation of substituted NICzs as well as for the incorporation of the NICz moiety into larger π‐conjugated systems. The pursued approaches are divided into two groups and the results of the respective reactions towards NICz precursors are summarized in Table 1.

**Table 1:** Synthesis of the precursors for C−H activation reactions towards mono‐substituted NICzs.

Precursor	Method	Halogen X	Yield [%]
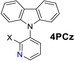	condensation^[a]^	Br	52
	Buchwald–Hartwig^[b]^	Br	–
	substitution^[c]^	Br	–^[e]^
	substitution^[c]^	Cl	69
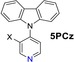	condensation^[a]^	Br	32
	Buchwald–Hartwig^[b]^	Br	81
	substitution^[c]^	Br	69
	substitution^[c]^	Cl	95
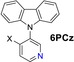	condensation^[a]^	Br	–
	Buchwald–Hartwig^[b]^	Br	67
	substitution^[c]^	Br	–^[f]^
	substitution^[c]^	Cl	57
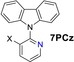	condensation^[a]^	Br	39^[g]^
	Buchwald–Hartwig^[b]^	Br	4
	substitution^[c]^	Br	37
	substitution^[c]^	Cl	94
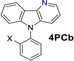	substitution^[d]^	Br	86
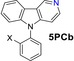	substitution^[d]^	Br	82
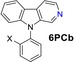	substitution^[d]^	Br	92
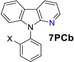	substitution^[d]^	Br	34

Reaction conditions: [a] condensation: 2,5‐dimethoxytetrahydrofuran (4 equiv), acetic acid, reflux. [b] Buchwald–Hartwig amination: 2,2′‐diiodo‐1,1′‐biphenyl (1 equiv), NaO*t*Bu (6 equiv), Pd_2_(dba)_3_ (2 mol %), 1,1′‐bis(diphenylphosphino)ferrocene (dppf, 4 mol %), toluene, reflux. [c] Nucleophilic aromatic substitution towards PCzs: dihalogenated pyridine (1 equiv), Cs_2_CO_3_ (1.1 equiv), DMF, 130 °C. [d] Nucleophilic substitution towards PCbs: 1‐bromo‐2‐fluorobenzene (2 equiv), Cs_2_CO_3_ (2 equiv), DMF, 130 °C. [e] Chloro‐**7PCz** was isolated in 35 % yield. [f] Chloro‐**5PCz** was isolated in 37 % yield. [g] Product contained inseparable byproducts.

The first strategy focused on the establishment of a carbazole unit attached to a halogen‐substituted pyridine. Notably, the outcome of the cyclization reaction for these precursors was solely determined by the substitution pattern of the pyridine. Accordingly, this strategy allowed for the preparation of NICzs with the nitrogen atom incorporated in the peripheral benzene unit of the ICz scaffold (Table 1, **4PCz**–**7PCz**, position 4–7). The most straightforward preparation of the respective precursors was a nucleophilic aromatic substitution of a appropriately substituted dihalogenated pyridine (Scheme 1). Following this approach, the brominated precursors bromo‐**5PCz** and bromo‐**7PCz** were obtained in 69 and 37 % yield, respectively, by heating the corresponding bromochloropyridines and carbazole in the presence of a base (see Table 1 for reaction conditions). The two remaining carbazole precursors, bromo‐**4PCz** and bromo‐**6PCz**, however, could not be prepared using this method. Instead, nucleophilic substitution occurred in the more activated 2 and 4 positions of the pyridines, giving chloro‐**7PCz** and chloro‐**5PCz** in low yields. Therefore, we used brominated aminopyridines as alternative substrates (Scheme 1); bromo‐**4PCz** was obtained by condensation of 3‐amino‐2‐bromopyridine and three equivalents of 2,5‐dimethoxytetrahydrofuran^50^ in 52 % yield. In the case of bromo‐**6PCz**, the carbazole moiety was formed by a Pd‐catalyzed Nozaki‐type Buchwald–Hartwig amination.^51^ Notably, the condensation reaction did not yield bromo‐**6PCz** and the Nozaki approach failed for the preparation of bromo‐**4PCz**. To fully explore the accessibility of the carbazole precursors, we also employed these two methods for the preparation of bromo‐**5PCz**, which was obtained in good yields from both reactions, as well as bromo‐**7PCz**, which could not be successfully prepared following these approaches.

The second strategy employs the four different carboline derivatives as starting materials (Scheme 1, right). The corresponding four carboline‐based NICz precursors (PCbs) were prepared by nucleophilic aromatic substitution of 1‐bromo‐2‐fluorobenzene and were obtained in excellent yields (82–92 %) with the exception of bromo‐**7PCb** (34 %; Table 1). In contrast to the PCz precursors, the unsymmetrical nature of carbolines **4PCb**, **5PCb**, and **6PCb** leads to two possible sites at which ring closure may occur. Although reactions at the benzene unit of the carbolines would yield the same products, which are available through the carbazole strategy, ring closure at the pyridine unit would give the two remaining NICz derivatives. Notably, the site selectivity and ratio of product is determined by the electronic demand of the ring‐closing reaction and thus, may be significantly influenced by the nitrogen position.

Furthermore, we aimed to explore the substrate scope of the C−H activation. Accordingly, the chloro‐PCz precursors were prepared starting from carbazole and the corresponding chlorofluoropyridines. In contrast to the bromo‐precursors all chloro‐derivatives were obtained by nucleophilic aromatic substitution in acceptable to excellent yield (57–95 %; Table 1).

### C−H activation to form nitrogen‐substituted indolo[3,2,1‐*jk*]carbazoles

**Scheme 2:**
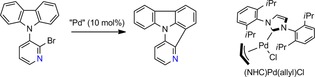
Synthesis of **4NICz** starting from bromo‐**4PCz** as a model system for the optimization of the reaction conditions; molecular structure of (NHC)Pd(allyl)Cl.

**Figure 2:**
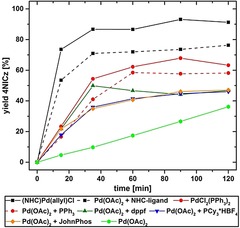
C−H activation of bromo‐**4PCz** to yield **4NICz** applying different catalysts. Reactions conditions: bromo‐**4PCz** (0.025 mmol), K_2_CO_3_ (2 equiv), catalyst (10 mol %), and ligand [12 mol %: N‐heterocyclic carbene (NHC), 1,1′‐bis(diphenylphosphino)ferrocene (dppf); 22 mol %: PPh_3_, PCy_3_⋅HBF_4_, (2‐biphenyl)di‐*tert*‐butylphosphine (JohnPhos)], DMA, 130 °C.

During initial experiments regarding the ring closing reaction by C−H activation, we encountered difficulties with reproducibility of reaction time and yields of the reactions. Thus, we decided to revisit the reaction conditions of this Pd‐catalyzed key step focusing on the example producing **4NICz** from bromo‐**4PCz** (Scheme 2, Table 2). First, various catalytic systems (catalyst loading: 10 mol %) were investigated by applying a catalyst based on an N‐heterocyclic carbene ligand [(NHC)Pd(allyl)Cl]^52^, ^53^ (Scheme 2) as well as different phosphine ligands, which were previously applied in the preparation of **ICz** 23, 32 or in other intramolecular arylation reactions.^54^, ^55^, ^56^ Yields were determined using GC (FID). As depicted in Figure 2, (NHC)Pd(allyl)Cl provided by far the best results with over 90 % yield within 90 minutes. In comparison, the use of phosphine‐based catalysts resulted in longer reaction times and lower yields (47–68 %) due to increased formation of dehalogenated byproduct (Figure S63, Supporting Information). Notably, the precursor salt of the ligand, [1,3‐bis(2,6‐diisopropylphenyl)‐1*H*‐imidazol‐3‐ium chloride], could also be applied directly in combination with Pd(OAc)_2_ 26 to give the product in 76 % yield after 120 min. Variations of the base (Figure S64, Supporting Information) and solvent decreased the reaction rate. Additionally, the reactivity of (NHC)Pd(allyl)Cl towards the chlorinated precursor chloro‐**4PCz** was investigated to explore the scope of the reaction. Notably, **4NICz** was obtained in 68 % yield after 180 min (Table 2, entry 16). In contrast, starting from chloro‐**4PCz**, the reaction with PdCl_2_(PPh_3_)_2_ gave **4NICz** with a yield of only 10 % after 180 min (Figure S65, Supporting Information), highlighting the importance of the NHC ligand. Accordingly, the combination of (NHC)Pd(allyl)Cl and K_2_CO_3_ in dimethylacetamide (DMA) provides the optimal reaction conditions. Hence, we decreased the catalyst loading to establish the final reaction conditions with a catalyst loading of 5 mol % (Figure S66, Supporting Information).

**Table 2:** Screening of the reaction conditions for the ring closure towards **4NICz** employing bromo‐**4PCz** (entries 1–15) or chloro‐**4PCz** (entries 16–17).

Entry	Catalyst	Ligand	Base^[a]^	Solvent^[b]^	*T* [°C]	*t* [min]^[c]^	Yield [%]^[d]^
1	Pd(OAc)_2_^[e]^	NHC⋅HCl^[f]^	K_2_CO_3_	DMA	130	120 (91 %)	76
2	PdCl_2_(PPh_3_)_2_^[e]^	–	K_2_CO_3_	DMA	130	60	68
3	Pd(OAc)_2_^[e]^	PPh_3_^[g]^	K_2_CO_3_	DMA	130	60	59
4	Pd(OAc)_2_^[e]^	dppf^[f]^	K_2_CO_3_	DMA	130	35	50
5	Pd(OAc)_2_^[e]^	PCy_3_⋅HBF_4_^[g]^	K_2_CO_3_	DMA	130	120 (65 %)	47
6	Pd(OAc)_2_^[e]^	JohnPhos^[g]^	K_2_CO_3_	DMA	130	120	47
7	Pd(OAc)_2_^[e]^	–	K_2_CO_3_	DMA	130	120 (71 %)	36
8	(NHC)Pd(allyl)Cl^[e]^	–	K_2_CO_3_	DMA	130	60	93
9	(NHC)Pd(allyl)Cl^[e]^	–	Na_2_CO_3_	DMA	130	180	94
10	(NHC)Pd(allyl)Cl^[e]^	–	K_3_PO_4_	DMA	130	60	84
11	(NHC)Pd(allyl)Cl^[e]^	–	Et_3_N	DMA	130	1200 (76 %)	10
12	(NHC)Pd(allyl)Cl^[e]^	–	K_2_CO_3_	toluene	110	150 (6 %)	3
13	(NHC)Pd(allyl)Cl^[e]^	–	K_2_CO_3_	dioxane	100	150 (4 %)	1
14	(NHC)Pd(allyl)Cl^[h]^	–	K_2_CO_3_	DMA	130	360	73
15	(NHC)Pd(allyl)Cl^[i]^	–	K_2_CO_3_	DMA	130	1200 (93 %)	52
16^[j]^	(NHC)Pd(allyl)Cl^[e]^	–	K_2_CO_3_	DMA	130	180 (72 %)	68
17^[j]^	PdCl_2_(PPh_3_)_2_^[e]^	–	K_2_CO_3_	DMA	130	180 (47 %)	10

[a] 2 equiv. [b] The water content of DMA was adjusted to 3000 ppm. [c] Reaction time until ≥97 % conversion was reached, unless noted otherwise in bracket. [d] Determined by GC‐FID analysis. [e] 10 mol %. [f] 12 mol %. [g] 22 mol %. [h] 5 mol %. [i] 2 mol %. [j] Starting from chloro‐**4PCz**.

Initial experiments on 1 mmol scale were performed using carbazole precursors **4PCz**–**7PCz** (Table 3). To our delight, the conversion of the bromo substrates smoothly delivered all four NICz derivatives (**4NICz**–**7NICz**) in excellent yields of 80–96 % after reasonable reaction times (4–8 h). Inspired by this initial success, we investigated the reactivity of the respective chloro precursors. Surprisingly, the results obtained for the chloro substrates in these quantitative experiments exceeded those of the bromo precursors. This finding contrasts previous results that suggested a lower reactivity of chloro substrates in the C−H activation step towards **ICz**.^23^ Nevertheless, **4NICz**–**7NICz** were obtained from the corresponding chloro‐**PCzs** in excellent yields between 92 and 98 % after a short reaction time of 4 h (Table 3). In the next step, we investigated the reactivity of the carboline precursors (PCbs, Table 4). In contrast to the carbazole strategy, two possible products can be formed in the C−H activation step from the PCbs. Although the formation of a product mixture is a clear disadvantage, **1NICz** and **2NICz** are only accessible through this strategy. Precursors **4PCb**–**6PCb** were all converted to ring‐closed NICzs. In contrast, hardly any conversion of **7PCb** was observed and starting material, along with small amounts of dehalogenated byproduct, was recovered even after prolonged reaction times of 96 h. We assume that the *ortho*‐nitrogen of **7PCb** stabilizes the initially formed Pd complex and thus prevents productive ring closure. Analyzing the products formed in the C−H activation, we determined that a regioselective ring closure occurred at the 4 (**4PCb**) and 3 (**5PCb**) positions of the pyridine ring. Accordingly, **1NICz** and **4NICz** were obtained from **4PCb** in a 3:1 ratio after separation, whereas the reaction of **5PCb** yielded **2NICz** and **5NICz** in a ratio of 6.9:1. Notably, the separation of the regioisomers was achieved by simple column chromatography. Thus, the two missing NICz regioisomers were indeed prepared using the carboline strategy. In the case of **6PCb**, ring closure on the benzene ring of the carboline was preferred, presumably owing to the highly electron‐poor alternative 2‐position of the pyridine. Therefore, **1NICz** and **6NICz** were formed in a ratio of 1:3.9.

**Table 3:** C−H activation towards mono‐substituted NICzs starting from carbazole precursors.

Precursor	Product	Halogen X	*t* [h]	Yield [%]
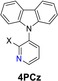	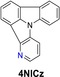	Br	6	93
		Cl	4	98
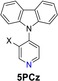	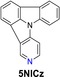	Br	4	96
		Cl	4	97
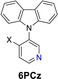	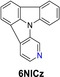	Br	4	80
		Cl	4	92
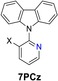	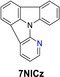	Br	8	84
		Cl	4	96

**Table 4:** C−H activation towards mono‐substituted NICzs starting from carboline precursors.

Precursor	*t* [h]	Product and yield [%]^[a]^	
		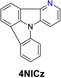	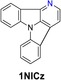
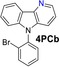	6	n.d.^[b]^/(15)	n.d.^[b]^/(45)
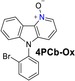	8	7/(3)	61/(58)
		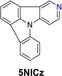	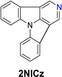
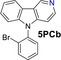	4	11/(0)	76/(46)
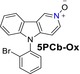	4	14/(4)	62/(44)
		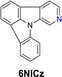	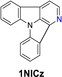
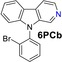	6	74/(61)	19/(16)
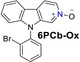	4	16/(12)	71/(57)

[a] Overall yield of ring‐closed products (ratio determined by ^1^H NMR) and yields of isolated material in brackets. For reactions starting from N‐oxides, yields over two steps (C−H activation and reduction) are given. [b] Not determined owing to overlapping signals from small amounts of dehalogenated byproduct.

Inspired by these results, we wondered if we could control the regioselectivity of the C−H activation by manipulation of the electron density of the pyridine unit. Thus, we oxidized the nitrogen atom of the pyridine to increase the electron density of the aromatic ring; the reaction with *meta*‐chloroperbenzoic acid (*m*CPBA) yielded the corresponding N‐oxides in good to excellent yields (61–90 %). Employing these N‐oxides, we indeed observed an increased tendency for the ring closure on the now more electron‐rich pyridine rings (Table 4), whereas high yields (89– 92 %) were retained. Reduction of the N‐oxides was smoothly accomplished using Fe powder in acetic acid (AcOH) in 76–97 % yields. In the case of **4PCb**, the ratio of isolated **1NICz** to **4NICz** was significantly shifted towards **1NICz** when starting form **4PCb‐Ox** (3:1 vs. 19.3:1). The ratio of the products remained roughly the same for **5PCb** and **5PCb‐Ox**. Most strikingly, however, the regioselectivity could be inverted for **6PCb**. Although **6NICz** is the favored product starting from **6PCb**, **1NCz** is formed predominately from **6PCb‐Ox** (**1NICz**:**6NICz**=4.4:1). This remarkable result underlines the impact of the electronic layout of the carboline systems on the outcome of the ring‐closing step. This oxidation–reduction sequence was established as a powerful tool to control the regioselectivity of the C−H activation reaction, which is of particular interest for the synthesis of more complex and/or extended annulated systems (see section on diazaindolo[3,2,1‐*jk*]carbazoles).

### Diazaindolo[3,2,1‐*jk*]carbazoles

Inspired by the successful preparation of all mono‐substituted NICz regioisomers, we explored the potential to further increase the electron deficiency of the ICz scaffold by double pyridine incorporation. Given the numerous possibilities of where to position two nitrogen atoms in the annulated system, we limited these investigations to substrates that would yield symmetrically substituted products **4,12NICz**, **5,11NICz**, and **6,10NICz** (Table 5). The respective precursors **4,12PyCb**, **5,11PyCb**, and **6,10PyCb** were prepared by nucleophilic substitution of suitably decorated dihalogenated pyridines with the respective carbolines. To our delight, these precursors also underwent smooth ring closure under the optimized conditions, giving the doubly substituted NICzs in high overall yields. In analogy to the mono substituted compounds, ring closure occurred on the pyridine ring in the case of **4,12PyCb** and **5,11PyCB**, preferentially yielding unsymmetrical **1,4NICz** and **2,5NICz**, respectively (Table 5). Separation of the regioisomers was accomplished by HPLC on the preparative scale with an acceptable product loss. In contrast, the reaction of **6,10PyCb** yielded symmetric **6,10NICz** as the major product. Compared with the mono‐substituted precursor **6PCb**, the electron‐deficient 2‐position of the pyridine ring was disfavored to a greater extent in **6,10PyCb** because symmetric **6,10NICz** and unsymmetrical **1,10NICz** were obtained in a ratio of 15.2:1. To control the regioselectivity of the C−H activation, we again employed the oxidation–reduction strategy. Indeed, the selectivity was inverted and **1,10NICz** was the preferred product starting from **6,10PyCb‐Ox** (**6,10NICz**:**1,10NICz**=1:12.7). The complete inversion of the regioselectivity clearly underlines the potential of the N‐oxide methodology to control the outcome of the ring‐closing step.

**Table 5:** C−H activation towards doubly substituted NICzs.

Precursor	*t* [h]	Product and yield [%]^[a]^	
		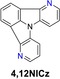	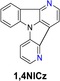
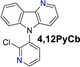	4	28/(21)	65/(40)
		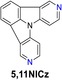	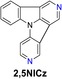
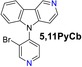	4	19/(15)	72/(53)
		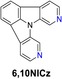	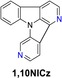
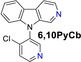	4	76/(76)	n.d. ^[b]^/(5)
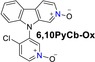	4	3/(3)	38/(38)

[a] Overall yield of ring‐closed products (ratio determined by ^1^H NMR) and yields of isolated material in brackets. For reactions starting from N‐oxides, yields over two steps (C−H activation and reduction) are given. [b] Not determined owing to overlapping signals from small amounts of dehalogenated byproduct.

### Characterization

After the isolation of all six possible mono‐substituted NICzs and three symmetrical doubly substituted NICzs as well as their respective unsymmetrical congeners, we investigated the molecular properties of the new building blocks.

#### Photophysical properties

**Figure 3:**
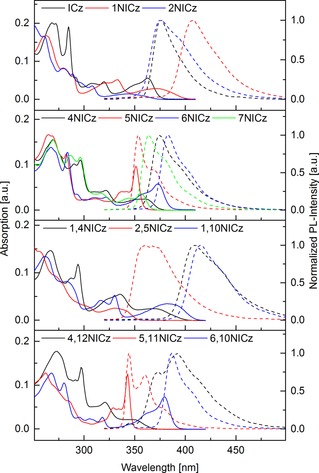
UV/Vis absorption (full lines) and normalized fluorescence spectra (dotted lines) of **ICz** and the synthesized NICz building blocks. All spectra recorded as 5 μm solutions in CH_2_Cl_2_.

**Figure 4:**
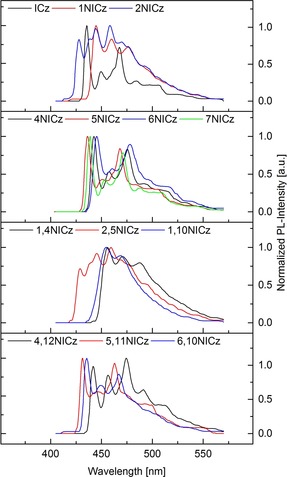
Normalized low‐temperature phosphorescence spectra (77 K) of **ICz** and the synthesized NICz building blocks.

To investigate the effects of the nitrogen incorporation on the photophysical properties of the synthesized building blocks, UV/Vis absorption and fluorescence spectra in dichloromethane, as well as low temperature phosphorescence spectra were recorded (Figures 3, 4, and Table 6). Molar attenuation coefficients are given in Table S1 (Supporting Information). Plain **ICz** shows a distinct absorption peak at 285 nm that is attributed to a π–π* transition of the conjugated molecular scaffold.^25^, ^26^ This signal disappeared almost completely with the incorporation of nitrogen in the doubly annulated central benzene ring in **1NICz** and **2NICz**. The lowest‐energy transition of **ICz** can be observed as a clear signal at 363 nm with a shoulder at shorter wavelength. In contrast, **1NICz** and **2NICz** show a rather broad and weak absorption at longer wavelengths. Although **ICz** and **2NICz** have nearly the same absorption onset at approximately 375 nm, the absorption onset of **1NICz** is clearly redshifted to 400 nm.

**Table 6:** Electrochemical and photophysical data of the developed materials.

	Opt. BG^[a]^ [eV]	*λ_max_* ^[b]^ [nm]	*E_T_* ^[c]^ [eV]	HOMO^[d]^ [eV]	LUMO^[d]^ [eV]
**ICz**	3.30	375	2.84	−5.78	−2.27
**1NICz**	3.10	407	2.79	−6.22	−2.68
**2NICz**	3.33	375	2.90	−6.28	−2.43
**4NICz**	3.30	375	2.80	−6.22	−2.57
**5NICz**	3.45	354	2.84	−6.32	−2.42
**6NICz**	3.21	384	2.78	−6.23	−2.62
**7NICz**	3.38	364	2.82	−6.00	−2.46
**1,4NICz**	3.10	410	2.72	−6.28	−2.84
**2,5NICz**	3.51	361	2.89	−6.48	−2.58
**1,10NICz**	3.03	416	2.72	−6.31	−2.85
**4,12NICz**	3.37	392	2.81	−6.35	−2.70
**5,11NICz**	3.56	345	2.87	−6.42	−2.54
**6,10NICz**	3.18	387	2.84	−6.44	−2.75

[a] Optical band gap determined from the absorption onset measured in CH_2_Cl_2_ solutions (5 μm) at room temperature. [b] Emission maximum measured in CH_2_Cl_2_solutions (5 μm) at room temperature. [c] Determined from the highest vibronic transition in solid solutions of toluene/EtOH (9:1; 0.5 mg mL^−1^) at 77 K. [d] Calculated from the onset of the oxidation and reduction peak observed during cyclic voltammetry.

The isomers with a nitrogen in one peripheral benzene ring show distinct peaks between 280–300 nm. Analogous to **ICz**, **5NICz** and **6NICz** exhibit a sharp absorption peak at about 285 nm. Although **7NICz** and **4NICz** feature peaks at 286 and 290 nm, respectively, those two compounds also feature additional further redshifted absorption peaks at 297 nm. At longer wavelengths, the absorption of **5NICz** and **6NICz** resembles that of **ICz** with the shoulder of a distinct peak oriented towards shorter wavelengths and absorption onsets at 359 and 386 nm, respectively. In contrast, **7NICz** and **4NICz** exhibit broader and weaker absorption bands with onsets at 366 and 375 nm, respectivley. Notably, nitrogen incorporation and the altered substitution position significantly impact the electronic layout and thus the absorption behavior of the NICz building blocks. Accordingly, the optical band gap of the mono‐substituted NICz, which is an important characteristic for practical applications, can be tuned over a range of 0.35 eV from 359 to 400 nm solely by choice of the nitrogen position.

The absorption characteristics of unsymmetrically, doubly nitrogen‐substituted derivatives **1,4NICz** and **1,10NICz** as well as **2,5NICz** clearly resemble those of **1NICz** and **2NICz** (Figure 3, Table 6). This result is in line with the finding that compounds **4**–**7NICz** more closely resemble plain **ICz**. Thus, nitrogen substitution in the central benzene ring is the crucial factor determining the absorption properties of the doubly substituted derivatives. Notably, the distinct absorption band of **4NICz** at 295 nm is also observed for **1,4NICz**. The onset of absorption of unsymmetrical **1,4NICz** is identical to that of **1NICz** at 400 nm, whereas the broad lowest‐energy transition of **1,10NICz** is shifted to longer wavelengths with an absorption onset of 410 nm. In contrast, the absorption onset of unsymmetrical isomer **2,5NICz** at 353 nm is shifted towards higher energy compared with **2NICz** (373 nm). Accordingly, the optical band gaps of the unsymmetrically, doubly substituted derivatives span a larger range of 0.49 eV compared with the mono‐substituted NICzs.

Symmetrical building blocks **4,12NICz**, **5,11NICz**, and **6,10NICz** exhibit absorption properties similar to their respective mono‐substituted congeners. The absorption onset of **4,12NICz** and **5,11NICz** are slightly blueshifted compared with **4NICz** and **5NICz**, whereas the onset of **6,10NICz** is shifted to a slightly longer wavelength than that of **6NICz**.

Both **1NICz** and **2NICz** exhibit similar fluorescence characteristics to those of **ICz**. Analogous to the absorption onset, the emission maximum of **2NICz** is found at the same wavelength as that for **ICz** (375 nm), whereas the emission of **1NICz** is distinctly redshifted to 407 nm. In contrast, all isomers with nitrogen substitution in the peripheral benzene ring exhibit a sharper emission peak in this area as well as a redshifted shoulder. The nitrogen position influences the emission maxima, which follow the same order as the absorption onset from **5NICz** (354 nm) to **7NICz** (364 nm), **4NICz** (375 nm), and **6NICz** (384 nm).

The unsymmetrical substituted isomers **1,4NICz**, **2,5NICz**, and **1,10NICz** each show one broad emission peak. Similar to **1NICz**, the emission maxima of **1,4NICz** and **1,10NICz** are rather redshifted at 410 and 416 nm, respectively. In contrast, **2,5NICz** exhibits an emission maximum at 361 nm, which is blueshifted compared with that of **2NICz** and closer to the emission of peripherally substituted **5NICz**. Analogous to the absorption properties, **5,11NICz** and **6,10NICz** feature similar emission properties as their respective mono‐substituted derivatives. Notably, **5,11NICz** exhibits the highest‐energy emission of the investigated materials with an emission maximum at 345 nm. Unlike the other derivatives, the emission characteristic of **4,12NICz** clearly differs from that of **4NICz**. Although the emission maximum of **4,12NICz** is redshifted to 392 nm compared with **4NICz** an additional high‐energy band at 372 nm emerges.

The triplet energy, corresponding to the highest‐energy phosphorescent transition, of building blocks for organic electronics is a decisive factor in particular for applications in OLEDs.^10^ Therefore, phosphorescence spectra of the developed building blocks were recorded. All compounds exhibit vibronically resolved phosphorescence. The phosphorescence spectra of the NICz isomers with the nitrogen in the peripheral ring are similar to the spectrum of **ICz**. Although the highest energy maximum of **5NICz** at 436 nm is the same compared to **ICz**, this transition is slightly redshifted for **7NICz** (439 nm), **4NICz** (443 nm), and **6NICz** (446 nm). Notably, this redshift occurs in the same order as for the fluorescence. In contrast, **1NICz** and **2NICz** show clearly different phosphorescence. The first maximum is blueshifted to 428 nm in the case of **2NICz**, but redshifted to 445 nm for **1NICz**. The spectra of the unsymmetrically doubly substituted **NICz** isomers are clearly impacted by the nitrogen atoms in the central benzene ring because they show great similarity to the spectra of **1NICz** and **2NICz**. Although **1,4NICz** and **1,10NICz** have highest energy maxima at 456 and 455 nm, respectively, **2,5NICz** is blueshifted to 429 nm. The spectra of the symmetrical doubly substituted isomers are similar to the peripheral mono‐substituted derivatives. Accordingly, the highest‐energy vibronic transitions of **5,11NICz** and **4,12NICz** are located at about the same energy as those of **5NICz** and **4NICz** at 432 and 442 nm, respectively. In contrast, this transition (436 nm) is slightly blueshifted for **6,10NICz** compared with **6NICz**. Notably, the triplet energies of the NICz derivatives are located between 2.90 and 2.72 eV and are therefore suitable for the development of functional organic materials for applications in blue, and even deep blue, phosphorescent and TADF‐based OLEDs.^57^

In summary, clear trends for the impact of the different nitrogen substitution positions were found. Substitution of one of the peripheral benzene rings (position 4–7, Figure 1) resulted in photophysical properties similar to those of plain **ICz**, in which the exact energetic localization of absorption and emission (blueshift, redshift) depended on the substitution position. Derivatives with symmetrical double substitution of the peripheral rings follow these tendencies, although the resulting shifts were generally more pronounced. In contrast, substitution of the central benzene ring (position 1 or 2) significantly altered the photophysical behavior, in which nitrogen incorporation at position 1 led to a shift towards lower energies and incorporation at position 2 towards higher energies. In the case of the unsymmetrical doubly substituted derivatives with one pyridine‐like nitrogen in the central ring and one in a peripheral benzene ring, the effect of the nitrogen in the central benzene ring clearly dominates and those derivatives feature similar properties to **1NICz** and **2NICz**.

#### Electrochemical properties

The exact energetic alignment of the highest occupied molecular orbitals (HOMOs) and lowest unoccupied molecular orbitals (LUMOs) of organic materials is of tremendous importance regarding charge transport in and charge injection into electronic devices. Therefore, cyclic voltammetry measurements were conducted to explore the effects of nitrogen incorporation and the impact of the nitrogen position on the frontier molecular orbitals (Figures S67–S73, Supporting Information). All NICz derivatives exhibited irreversible oxidation, as typically observed for 9*H*‐carbazole and ICz derivatives, owing to the instability of the formed oxidation products.^25^, ^58^

**Figure 5:**
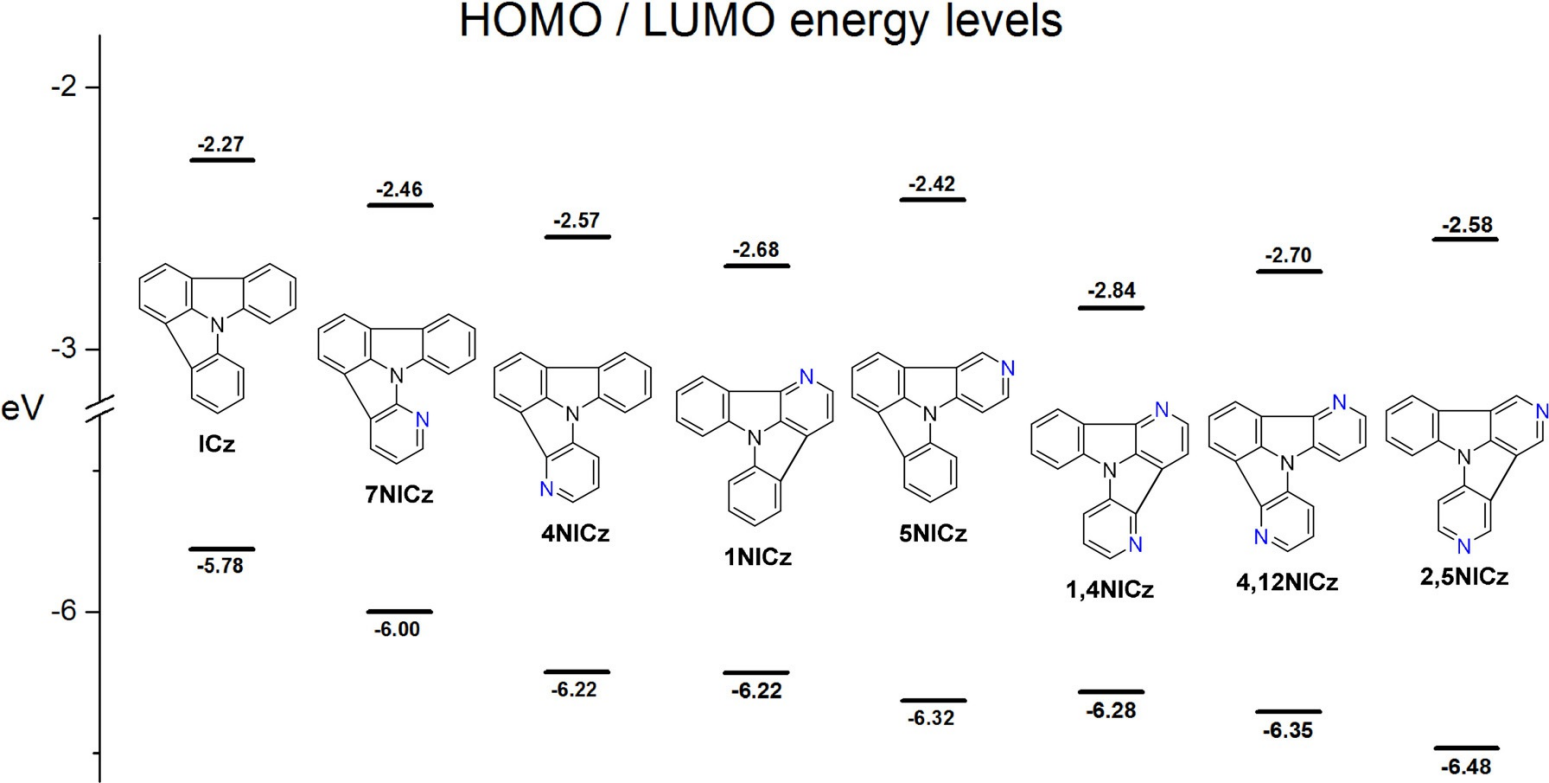
Schematic representation of the energy levels of HOMOs and LUMOs of selected materials.

The determined energy values of the HOMOs and LUMOs of the developed NICzs are summarized in Table 6 and selected energy levels are depicted in Figure 5. In general, nitrogen incorporation decreased the energy of both orbitals of all compounds compared with **ICz** (HOMO: −5.78 eV; LUMO: −2.27), the effect of which is slightly more pronounced for the HOMO levels. The HOMO levels of the mono‐substituted NICzs were decreased by 0.22–0.54 eV, whereas the LUMO energy levels are lowered by 0.15–0.41 eV. In the case of the doubly substituted NICzs, HOMO and LUMO levels are decreased by 0.50–0.70 and 0.27–0.58 eV, respectively. Notably, the magnitude of this effect is dependent on the exact position of the pyridine nitrogen in the **ICz** scaffold.

For the mono‐substituted NICz variants, the biggest impact on the HOMO levels is observed for compounds **2NICz** (−6.28 eV) and **5NICz** (−6.32 eV) with the pyridine nitrogen *para* to the central nitrogen atom. Notably, the effect on the LUMO levels of these two compounds is distinctly weaker and thus **2NICz** (−2.43 eV) and **5NICz** (−2.42 eV) feature the highest LUMO levels of the mono‐substituted series. In contrast, the overall effect of nitrogen incorporation is least pronounced for *ortho*‐substituted **7NICz**. Therefore, within this series, **7NICz** exhibits the highest HOMO energy of −6.00 eV, corresponding to a 0.22 eV decrease compared with **ICz**, and a LUMO level at −2.46 eV. Compounds **1NICz**, **4NICz**, and **6NICz** with a *meta*‐substitution pattern display similar electrochemical behavior. The LUMO levels of these three compounds are impacted the most by nitrogen incorporation and decrease to −2.68, −2.57, and −2.62 eV, respectively, whereas their HOMO levels are located in a narrow range of −6.22 to −6.23 eV between those of the *ortho*‐ and *para*‐substituted materials.

**Figure 6:**
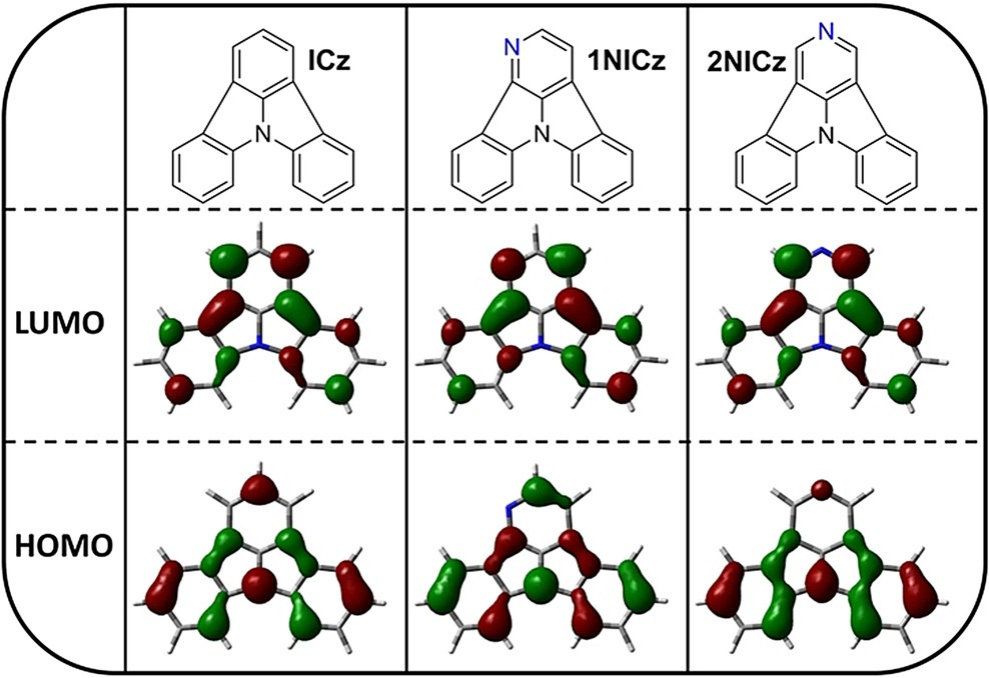
Contour plots of the spatial distribution of the HOMO and LUMO levels of **ICz**, **1NICz**, and **2NICz**.

The influence of the nitrogen position on the electrochemical properties was rationalized by the exploration of the spatial distribution of the HOMO and the LUMO of the investigated derivatives (Figures 6 and S74, S75, Supporting Information). HOMO and LUMO levels of the materials were calculated employing density functional theory. As depicted in Figure 6, significant electron density of the HOMO level of **ICz** is located on the central nitrogen, as well as C2, C5, and C11, which are located *para* to the central nitrogen. Analogously, hardly any electron density is located on C1, C3, C6, and C10, which are all located *meta* to the central nitrogen atom. The distribution of the electron density explains why *para*‐nitrogen substitution influences the energetic location of the HOMO levels the most. Indeed, the HOMO level of **2NICz** is strongly distorted compared with **ICz** (Figure 6). The concentration of the electron density on the two benzene rings and the reduction of the spatial extension of the molecular orbital decrease the orbital energy of the HOMO of **2NICz**. In contrast, the shape of the HOMO of **1NICz** is very similar to that of **ICz** and in the case of the *meta*‐substituted NICz, the decreased orbital energy can be explained by a general inductive effect of the pyridine nitrogen. The electron density of the LUMO of **ICz** is located virtually exclusively on carbon atoms that are *meta* to the central nitrogen and the annulation positions. This finding explains the significantly decreased energy of the LUMOs of **1NICz**, **4NICz**, and **6NICz**. Analogous to the HOMO of **2NICz**, a similar distortion of the shape of the LUMO of **1NICz** is observed. However, the restriction of the LUMO is less pronounced, which is in agreement with the finding that the LUMO energy levels are generally influenced to a slightly lower degree by the nitrogen incorporation. As expected, the energy levels of the HOMOs and LUMOs are further lowered by the introduction of a second pyridine ring. Compared with **ICz**, the HOMO levels of the doubly substituted NICzs are decreased by 0.50–0.70 eV and the LUMO energy levels are lowered by 0.27–0.58 eV. The same trends as for the mono‐substituted derivatives were observed. Accordingly, *para*‐substituted **2,5NICz** exhibits the lowest HOMO energy level among the compounds in this study at −6.48 eV whereas the HOMO level of **5,11NICz** is located slightly higher at −6.42 eV. Analogous to **2NICz** and **5NICz**, the LUMO levels of the doubly substituted derivatives **2,5NICz** and **5,11NICz** are located at −2.58 and −2.54 eV and thus are significantly higher compared with the LUMOs of the *meta*‐substituted compounds. The *meta*‐substituted derivatives can be divided into two groups; unsymmetrical **1,4NICz** and **1,10NICz** feature a smaller electrochemical band gap compared with symmetrical **4,12NICz** and **6,10NICz**. Accordingly, **1,4NICz** and **1,10NICz** exhibit the lowest LUMO energy levels among the developed materials (−2.84 and −2.85 eV, respectively).

#### Crystal packing

The arrangement and π–π interactions of individual molecules in the solid state can significantly impact the macroscopic photophysical and electronic properties of thin films of organic materials.^59^ In particular, C−H⋅⋅⋅N hydrogen bonds can be used to induce or control specific interactions between molecules and influence their alignment.^46^, ^47^ Hence, we were interested in the packing of the newly developed NICz molecules because the incorporation of the pyridine‐like nitrogen atom into the molecular scaffold can lead to the potential induction of C−H⋅⋅⋅N hydrogen bonding. Therefore, single crystals suitable for X‐ray structure determination were grown by recrystallization of **2NICz**, **5NICz**, **6NICz**, **7NICz**, **2,5NICz**, and **6,10NICz**. Crystals of **1NICz** were obtained only as the acetonitrile solvate. Compounds **2NICz**, **5NICz**, and **2,5NICz** featured temperature‐dependent polymorphism and twinning, which is beyond the scope of this contribution and will be discussed in detail elsewhere.^60^ Atoms were labeled as outlined in Figure 1. In the case of more than one crystallographically unique molecule (*Z*′=2), prime characters are added to the atom names.

The molecular packing in the solid state is generally determined by non‐classical C−H⋅⋅⋅N hydrogen bonding.^61^, ^62^ In these kind of non‐classical hydrogen bonds, electrostatic interactions are more prominent than covalent bonding.^61^ Their influence on the structure is not as pronounced as in the case of strong hydrogen bonds. Notably, some of the interactions reported here feature H⋅⋅⋅N distances longer than the sum of their van der Waals radii (2.75 Å). If possible, the lone pairs of the N atoms connect to the hydrogen atoms at the 7 and 9 positions (Table S2, Supporting Information). In a few exceptions, the H atom in the 2 position acts as donor. From a topological point of view, the hydrogen contacts form one‐dimensional chains, which are usually connected by π–π interactions to layers. The layers, in turn, are stacked to give the final crystal structure. A more detailed description of the molecular arrangements will be given first for the molecules with N‐substitution *para* to the central N8 atom (**2NICz**, **5NICz**, **2,5NICz**), then *meta* (**1NICz**, **1,10NICz**, **6NICz**, **6,10NICz**), and finally *ortho* (**7NICz**).

**Figure 7:**
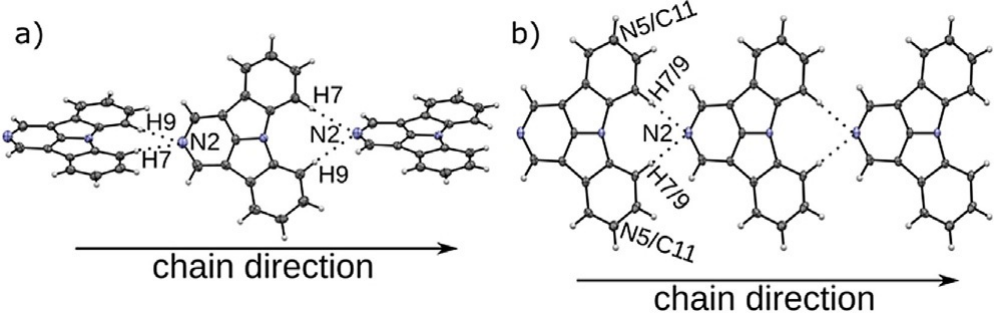
Chains of a) **2NICz** and b) **2**,**5NICz** molecules, second polymorph, connected by non‐classical C−H⋅⋅⋅N hydrogen bonding. C (grey) and N (blue) atoms are represented by ellipsoids drawn at the 50 % probability levels; H atoms by white spheres of arbitrary radius. The hydrogen bonds are indicated by dotted lines.

***para*** **‐N‐substitution**: The hydrogen bonding of **2NICz** and **2,5NICz** molecules forms straight chains as shown in Figure 7. In both cases, the molecules are located on a (pseudo‐)twofold rotation axis; this (pseudo)symmetry is the crucial feature of the phase transitions described elsewhere.^60^ The mutual inclination of adjacent molecules with respect to this axis is determined by the crystal packing: In **2NICz**, the least‐squares (LS) planes defined by the C and N atoms of the individual aromatic planes of two adjacent molecules are inclined by 63.4°. In contrast, **2,5NICz** exists as two polymorphs; the first is isostructural with **2NICz**, whereas in the second, adjacent molecules are perfectly coplanar (adjacent molecules related by a *b+c* lattice translation). In both **2,5NICz** polymorphs, the molecule is 1:1 disordered with respect to the N5 atom, which is not involved in hydrogen bonding.

**Figure 8:**
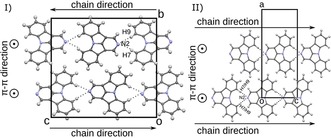
Packing of chains of a) **2NICz** and b) **2**,**5NICz** second polymorph. Color codes as in Figure 7.

The chains are in all cases connected to layers by π–π interactions. Adjacent chains within these layers are related by an *a* (**2NICz**, **2,5NICz** first polymorph) or a *b* (**2,5NICz**, second polymorph) lattice translation. The distances between the least‐squares planes of individual molecules of neighboring chains are 3.45 Å in all cases. Neighboring chains of two adjacent layers feature different propagation directions for the two structures (Figure 8). In the crystals of **2NICz** and the first polymorph of **2,5NICz**, the chains are parallel and extend in opposite directions. In contrast, in the second polymorph of **2,5NICz**, neighboring interlayer chains are inclined by 42.71° but propagate in the same direction. No notable interlayer C−H⋅⋅⋅π interactions are observed in either of the structures.

**Figure 9:**
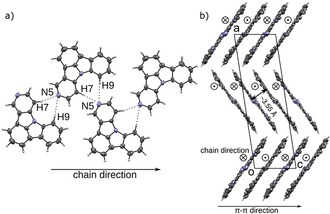
a) Chain of **5NICz** molecules in the solid state. b) Packing of **5NICz**. Color codes as in Figure 7.

For **5NICz**, analogous chains form in the solid state (Figure 9 a). Here, the hydrogen bond acceptor N5 is located off the molecular axis (C2−N8). Accordingly, the H7⋅⋅⋅N5 and H9⋅⋅⋅N5 contacts and the molecular axis form angles of approximately 90° and the formed molecular chains adopt a zig‐zag pattern. Adjacent molecules in these chains are related by a 2_1_ screw rotation axis and are nearly coplanar (angle between LS planes: 3.55°). As previously observed, the chains are connected by π–π interactions, in which adjacent chains are related by inversion symmetry (Figure 9 b). The distance between connected molecules is approximately 3.55 Å (a precise value cannot be given because the connected molecules are not perfectly coplanar). The formed layers are stacked along [100]. In principle, nitrogen atoms at the 2 and 5 positions can form similar C−H⋅⋅⋅N hydrogen bonds with the hydrogens at the 7 and 9 positions. Yet, only N2 is involved in hydrogen bonding in **2,5NICz**, indicating that this interaction is preferred.

***meta*** **‐N substitution**: Concerning the *meta*‐substituted **1NICz**, only acetonitrile solvate crystals were formed, in which the electron lone pair of N1 is directed towards the solvent filled voids of the structure (Figure S76, Supporting Information). This is a first indication that the formation of a hydrogen‐bond network is more difficult in these cases. The **1NICz** molecules are connected through π–π interactions to rods extending along the [100] direction (Adjacent molecules related by an *a*‐lattice translation; distance of LP planes: 3.41 Å); these rods surround the solvent filled voids.

**Figure 10:**
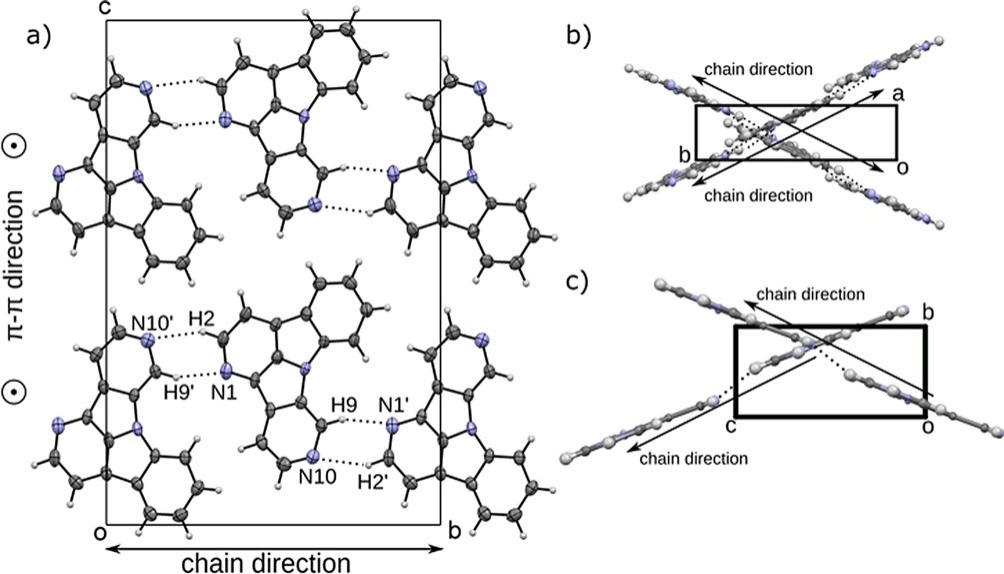
a) Packing of chains of **1**,**10NICz** and side view of chains of b) **1**,**10NICz** and c) **2**,**5NICz**. Color codes as in Figure 7.

**Figure 11:**
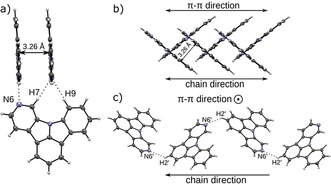
a) Three **6NICz** molecules (molecule 1) connected through C−H⋅⋅⋅N contacts to a chain fragment and b) resulting chain. c) Chain of **6NICz** molecules (molecule 2) connected through C−H⋅⋅⋅N contacts that are not structure‐directing. Color codes as in Figure 7.

Crystals of **1,10NICz**, in contrast, consist of two crystallographically independent molecules. These molecules are connected by H2⋅⋅⋅N10′ and H9′⋅⋅⋅N1 contacts, with each molecule of the pair acting as donor and acceptor (Figure 10 a). Adjacent molecules are related by a pseudo‐*b*_[001]_ glide reflection (the *P*2_1_ structure has pseudo‐*Pc*2_1_ *b* symmetry) forming chains extending in the [010] direction. The two crystallographically independent molecules are nearly coplanar (angle between LS planes: 3.65°). Analogous to **2NICz** and **2,5NICz**, the chains are connected to layers by π–π interactions (adjacent chains related by an *a*‐lattice translation; distance of LS planes 3.47 Å in both cases). Notably, the arrangement of the **1,10NICz** chains closely resembles the structure formed by **2,5NICz** (second polymorph). Chains in neighboring layers propagate in the same direction and the angle between the chains is 56.41° (Figure 10 b and 10 c). The hydrogen‐bonding networks in *meta*‐substituted **6NICz** is complex. Crystals of **6NICz** contain two crystallographically independent **6NICz** molecules (*Z′*=2). In contrast to **1,10NICz** these molecules are not related by pseudo‐symmetry. Molecules of the first kind are connected by C−H⋅⋅⋅N hydrogen bonds, in which each molecule connects to two others, acting as hydrogen‐bond donor and acceptor, respectively. Because the donor (H7, H9) and acceptor (N6) atoms are in close vicinity, a strong twisting of the two connecting molecules is required (angle between LS planes: 77.8°; Figure 11 a). The two molecules connected as donor and acceptor to one twisted molecule are coplanar (related by a *b*‐lattice translation) and their LS planes are spaced by only 3.26 Å, indicating a strong interaction of the π systems (Figure 11 b). These fragments form chains extending along the [010] direction. Notably, π–π stacking does not occur between adjacent chains but between two molecules of the same chain, which are connected by a third **6NICz** molecule that acts as a hydrogen donor and acceptor for the stacked molecules, respectively (Figure 11 a). Therefore, in contrast to the previous cases, the π–π interaction does not connect adjacent chains to two‐dimensional layers but forms one‐dimensional rods extending along [010] by intra‐chain π–π interactions. For the second kind of **6NICz** molecule in the unit cell, a rather short C−H⋅⋅⋅N contact to a H2 atom is observed. The observed C−H⋅⋅⋅N angle of 135° indicates a rather weak interaction that is probably not structure‐directing. These weak interactions form chains extending in the [001] direction (Figure 11 c). Analogous to **2NICz**, these chains are connected to layers by π–π interactions through a *b**‐***lattice translation with a distance between the LS planes of 3.31 Å. Thus, in the case of **6NICz**, the π–π interaction between the planar molecules results in different packing features for the two crystallographically independent molecules. Although intrachain stacking yields one‐dimensional rods, interchain stacking leads to two‐dimensional layers.

Finally, the entire **6NICz** crystal is built by stacking two alternating layers, the first of which corresponds to the layer formed by the π–π stacking of chains of molecule 2 and the second is made of neighboring rods of molecule 1 that are packed by van der Waals interactions (Figure S77, Supporting Information).

**Figure 12:**
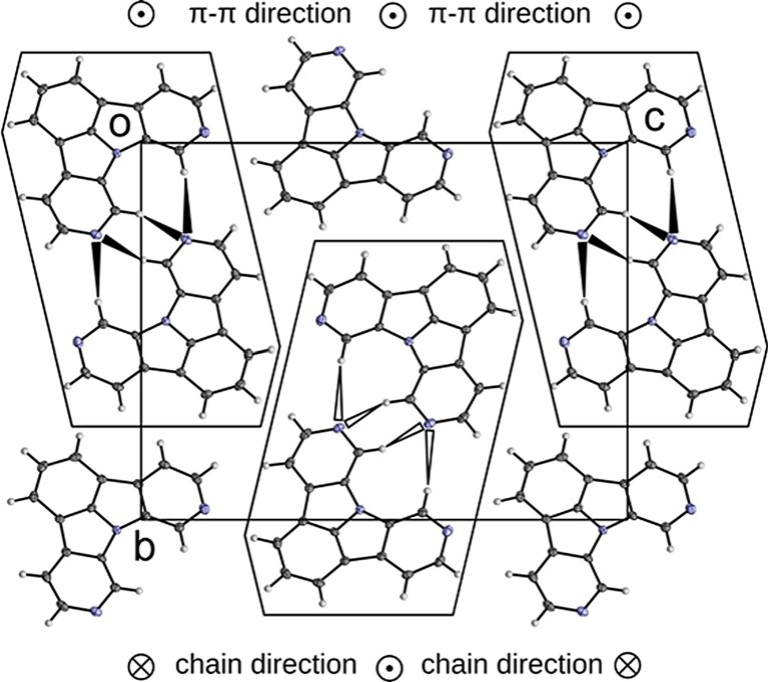
Packing of **6**,**10NICz**. Each **6**,**10NICz** molecule connects as hydrogen‐bond donor and hydrogen‐bond acceptor to two different adjacent **6**,**10NICz** molecules. Color codes as in Figure 7.

In **6,10NICz**, only one of the N atoms is involved in hydrogen bonding. Similar to **6NICz**, the connected molecules (related by a 2_1_ screw rotation) are distinctly non‐coplanar (angle between LS planes: 53.0°). In the resulting chains (Figure S78, Supporting Information), pairs of molecules are again connected by intrachain π–π interactions (distance between LS planes: 3.41 Å) and form rods along [100]. Thus, even though the chains are related topologically to one of the **6NICz** chains (considering hydrogen bonding and π–π interactions), the actual geometry of the chains is influenced distinctly by packing effects. The chains are packed in a checkerboard pattern connected only by van der Waals interactions (Figure 12).

***ortho*** **‐N substitution**: Finally, in **7NICz**, no notable intermolecular hydrogen‐bonding interactions are observed, owing to steric shielding of the nitrogen atom in the 7 position. Instead, molecules are connected by π–π interactions to rods (adjacent molecules related by an *a*‐lattice translation; distance of LS planes: 3.30 Å); the rods are packed by van der Waals interactions (Figure S79, Supporting Information).

In summary, the investigated NICzs exhibited a rich crystallization behavior, in which π–π stacking and non‐classical C−H⋅⋅⋅N hydrogen bonds proved to be structure‐determining factors. Although all compounds featured π–π stacking of the planar aromatic scaffolds, intermolecular hydrogen bonds were not formed in crystals of **1NICz** and **7NICz**. Notably, the sole pyridine‐like nitrogen of these two compounds is located next to the annulation position and thus is not effectively available for hydrogen bonding due to steric reasons. Although the interactions between isolated molecules of the developed compounds are similar, the position of the nitrogen has a decisive impact on the exact packing of the crystals. Thus, depending on the nitrogen position one‐ or two‐dimensional supramolecular arrangements are formed. Compounds lacking hydrogen bonds are organized in rods by π–π stacking; the rods are packed by van der Waals interactions (**1NICz**, **7NICz**). When hydrogen bonding is possible, chains are formed and connect to neighboring chains by π–π stacking to ultimately form layers (**2NICz**, **5NICz**, **2,5NICz**, **1,10NICz**). Notably, the propagation direction of chains in adjacent layers depends on the nitrogen position. If the geometrical requirements of the hydrogen bonds prevent linear propagation of the chains in the direction of the molecular axis (C2−N8), twisted chains form and π–π stacking occurs between molecules of the same chain (**6NICz**, **6,10NICz**). In such a way, the chains are arranged to one‐dimensional rods.

## Conclusion

We have described the convenient synthesis of all six possible mono‐substituted azaindolo[3,2,1‐*jk*]carbazoles as well as three symmetric doubly substituted derivatives and their unsymmetrical regioisomers. Notably, the presented C−H activation approach not only allows for the preparation of the azaindolo[3,2,1‐*jk*]carbazole congeners described in this work, but the developed oxidation–reduction strategy also enables one to control the regioselectivity of the ring‐closing C−H activation step. Therefore, many other doubly substituted regioisomers are now accessible employing this method and additional nitrogen incorporation can be achieved. We investigated the photophysical and electrochemical properties as well as the solid‐state interactions of the developed materials and revealed the impact of nitrogen incorporation in different positions of the indolo[3,2,1‐*jk*]carbazole scaffold. Ultimately, we have provided synthetic chemists with a toolbox full of NICz building blocks. The established structure–property relationships and the possibility to tune their molecular properties and control the supramolecular arrangement of individual molecules predestines the class of azaindolo[3,2,1‐*jk*]carbazoles as a novel molecular platform and could guide material scientists in the design of functional organic materials with tailored properties.

## Experimental Section

All reagents and solvents were obtained commercially and used without further purification. Anhydrous solvents were prepared by filtration through drying columns. The water content of purchased DMA was determined by Karl Fischer titration using a Mitsubishi CA‐21 Moisture Meter and corrected to 3000 ppm for C−H activation screening reactions. Given that further experiments indicated no significant impact of the water content on the reaction outcome, large scale experiments were conducted using unmodified DMA with roughly 100 ppm H_2_O. Screening reactions were performed in glass vials under argon atmosphere in a controlled heating block using argon‐saturated substrate solutions including 1‐methylnaphthalene as internal standard as well as a catalyst solution. All screening experiments were performed three times independently. The depicted results represent the mean of these experiments. Yields of the screening reactions were determined by GC using a Thermo Scientific TRACE 1310 gas chromatograph with dual configuration consisting of two AS 1310 autosamplers, Thermo Scientific TR‐5MS columns and FID detectors. Absorption and photoluminescence measurements were conducted using a PerkinElmer Lambda *750* spectrometer and a PerkinElmer LS 55 fluorescence spectrometer, respectively. CH_2_Cl_2_ solutions (5 μm) were employed for solution measurements whereas phosphorescence spectra were recorded at 77 K using solid solutions of the materials in toluene/EtOH (9/1; 0.5 mg mL^−1^) with a delay of 1 ms. Cyclic voltammetry was measured using a three‐electrode configuration consisting of a Pt working electrode and counter electrode, and an Ag/AgCl reference electrode and a PGSTAT128N potentiostat provided by Metrohm Autolab B.V. The measurements were carried out in a 0.5 mm solution in HPLC‐grade acetonitrile employing *n*Bu_4_NBF_4_ (0.1 m) as supporting electrolyte. Prior to the measurements, the solutions were purged with nitrogen for approximately 15 minutes. The HOMO and LUMO energy levels were calculated from the onset of the oxidation and reduction peaks, respectively. The onset potential was determined by the intersection of two tangents drawn at the background and the rising of the oxidation and reduction peaks. Synthetic details are described in the Supporting Information.

**General procedure for C−H activation**: A glass vial was charged with the corresponding halogenated precursor (1 equiv.), K_2_CO_3_ (2 equiv.), and (NHC)Pd(allyl)Cl (5 mol %) and flushed with argon. After addition of 10 mL mmol^−1^ degassed DMA, the reaction was stirred under argon atmosphere at 130 °C until full conversion was reached (4–8 h). After cooling, the reaction mixture was poured into water and repeatedly extracted with CH_2_Cl_2_. The organic phases were dried over anhydrous Na_2_SO_4_ and concentrated under reduced pressure. The crude product was purified by column chromatography.

**Computational details**: DFT calculations were performed using the Gaussian 09 package^63^ applying the Becke three‐parameter hybrid functional with Lee–Yang–Perdew correlation (B3LYP)^64^, ^65^ in combination with Pople basis sets 6‐311G(d,p).^66^ Geometry optimizations were performed in the gas phase and without symmetry constraints. Orbital plots were generated using GaussView.^67^

**Single‐crystal diffraction**: X‐ray diffraction data of **1NICz**⋅*x*CH_3_CN, **2NICz**, **5NICz**, **6NICz**, **7NICz**, **1,10NICz**, **2,5NICz**, and **6,10NICz** were collected at *T=*100–270 K in a dry stream of nitrogen on a Bruker Kappa APEX II diffractometer system using graphite‐monochromatized MoK_α_ radiation (*λ*=0.71073 Å) and fine sliced *ϕ*‐ and ω‐scans. Data was reduced to intensity values with SAINT and an absorption correction was applied with the multi‐scan approach implemented in SADABS or TWINABS.^68^ The structures were solved by the dual‐space approach implemented in SHELXT^69^ and refined against *F* ^*2*^ with JANA2006.^70^ Non‐hydrogen atoms were refined anisotropically. The H atoms connected to C atoms were placed in calculated positions and thereafter refined as riding on the parent atoms. Contributions of disordered solvent molecules to the intensity data were removed for **1NICz** using the SQUEEZE routine of the PLATON^71^ software suite. Compounds **2NICz**, **5NICz**, and **1,10NICz** crystallize as twins by pseudo‐merohedry. Compound **7NICz** forms twins with non‐overlapping reflections and was refined against HKLF5 data with information on reflection overlap. The absolute structure of non‐centrosymmetric crystals (**1NICz**⋅*x*CH_3_CN, **1,10NICz**, **2,5NICz**, and **6,10NICz**) was not determined owing to a lack of resonant scatterers. Molecular graphics were generated with the program MERCURY.^72^ CCDC 1864321 (**1,10NICz**⋅*x*CH_3_CN), 1864322 (**1NICz**⋅*x*CH_3_CN), 1864323 (2,**5NICz**), 1864324 (**2NICz**), 1864325 (**5NICz**), 1864326 (**6,10NICz**), 1864327 (**6NICz**) and 1864328 (**7NICz**) contain the supplementary crystallographic data. These data can be obtained free of charge by The Cambridge Crystallographic Data Centre.

## Conflict of interest

The authors declare no conflict of interest.

## Supporting information

As a service to our authors and readers, this journal provides supporting information supplied by the authors. Such materials are peer reviewed and may be re‐organized for online delivery, but are not copy‐edited or typeset. Technical support issues arising from supporting information (other than missing files) should be addressed to the authors.

Supplementary
