# Supplementary material for: Azaindolo[3,2,1‐jk]carbazoles: New Building Blocks for Functional Organic Materials
Source: Chemistry. 2019 Feb 27;25(17):4412–25. doi: 10.1002/chem.201805578 (PMC6491992; doi:10.1002/chem.201805578)
Supplement: Supplementary file 1 — Supplementary [file CHEM-25-4412-s001.pdf]

CHEMISTRY
A European Journal

# Supporting Information

## Azaindolo[3,2,1-*jk*]carbazoles: New Building Blocks for Functional Organic Materials

Thomas Kader,[a] Berthold Stöger,[b] Johannes Fröhlich,[a] and Paul Kautny*[a]

chem_201805578_sm_miscellaneous_information.pdf

**Table of contents**


1 General information ........ 2

2 Experimental procedures ........ 2

2.1 Synthesis of the precursors ........ 2

General procedure for the nucleophilic aromatic substitution reactions towards carbazole precursors (GP1-A). ........ 2

General procedure for the condensation reactions (GP2). ........ 2

General procedure for the Nozaki type Buchwald Hartwig amination (GP3). ........ 3

General procedure for the nucleophilic aromatic substitution reactions towards carboline precursors (GP1-B). ........ 8

General procedure for the N-oxide preparation (GP4). ........ 11

2.2 Synthesis of mono substituted NICzs ........ 15

General procedure for the ring closing C-H activation reactions (GP5). ........ 15

General procedure for the reduction of the N-oxides (GP6). ........ 18

2.3 Synthesis of twofold substituted NICzs ........ 22

3 NMR Spectra ........ 25

4 Additional Screening results ........ 56

5 Molar attenuation coefficient ........ 58

6 Cyclic Voltammetry ........ 58

7 HOMO / LUMO energy levels ........ 61

8 Crystal packing ........ 63

9 References ........ 66

# 1 General information

Column chromatography was performed on silica 60 (Merck, 40-63 μm). For preparative HPLC a Buchi Reveleris Prep Purification System with a Phenomenex Luna Prep silica (2) column (10 μm) was used. NMR spectra were recorded on a Bruker Avance DRX-400 Spectrometer. An Agilent 6230 LC TOFMS mass spectrometer equipped with an Agilent Dual AJS ESI-Source was used for high resolution mass spectrometry.

# 2 Experimental procedures

9*H*-Pyrido[2,3-*b*]indole,[1] 9*H*-pyrido[3,4-*b*]indole,[2] 5*H*-pyrido[4,3-*b*]indole,[3] 5*H*-pyrido[3,2-*b*]indole,[4] 2,2'-diiodo-1,1'-biphenyl[5] and allyl[1,3-bis(2,6-diisopropylphenyl)imidazol-2-ylidene]chloropalladium(II) ((NHC)Pd(allyl)Cl)[6] were synthesized according to literature.

## 2.1 Synthesis of the precursors

**General procedure for the nucleophilic aromatic substitution reactions towards carbazole precursors (GP1-A).** Carbazole (1 eq.) and $Cs_2CO_3$ (1.1 eq.) were placed in a glass vial and flushed with argon. DMF (2 ml/mmol) and the corresponding pyridine (1 eq.) were added and the reaction was stirred at 130 °C until full conversion (16 h – 20 h). After cooling, the reaction mixture was poured into water and repeatedly extracted with $CH_2Cl_2$. The organic phases were dried over anhydrous $Na_2SO_4$ and concentrated under reduced pressure. The crude product was purified by column chromatography.

**General procedure for the condensation reactions (GP2).** 2,5-Dimethoxytetrahydrofuran (4 eq.) was added to a solution of the corresponding brominated aminopyridine (1 eq.) in acetic acid (15 ml/mmol). The reaction mixture was refluxed under argon atmosphere until full conversion according to GC-MS (18 h - 120 h). Depending on the progress of the reaction further 2,5-dimethoxytetrahydrofuran was added. After cooling, the reaction mixture was

poured into cold 1N HCl and repeatedly extracted with $CH_2Cl_2$. The combined organic layers were washed with water and 2N NaOH, dried over anhydrous $Na_2SO_4$ and concentrated under reduced pressure.

**General procedure for the Nozaki type Buchwald Hartwig amination (GP3).** $NaO^tBu$ (6 eq.) was added to a solution of the corresponding brominated aminopyridine (1 eq.), 2,2′-diiodo-1,1′-biphenyl (1 eq.), $Pd_2(dba)_3$ (2 mol%) and 1,1′-bis(diphenylphosphino)ferrocene (4 mol%) in degassed anhydrous toluene (4 ml/mmol) under argon atmosphere in a three-necked flask. The reaction mixture was refluxed overnight. After cooling, the reaction mixture was filtered through a celite pad and washed with $CH_2Cl_2$. The filtrate was concentrated under reduced pressure and purified by column chromatography.

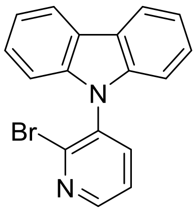

bromo-**4PCz**

*9-(2-Bromopyridin-3-yl)-9H-carbazole (bromo-**4PCz**).* Compound bromo-**4PCz** was prepared according to GP2 starting from 3-amino-2-bromopyridine (1.73 g, 10.0 mmol) and 2,5-dimethoxytetrahydrofuran (5.29 g, 40.0 mmol). The crude product was flashed over a silica pad ($CH_2Cl_2$). Recrystallization from EtOH gave bromo-**4PCz** (1.69 g, 5.23 mmol, 52%) as orange crystals. $^1H$ NMR (400 MHz, $CDCl_3$) δ 8.60 (dd, J = 4.7, 1.8 Hz, 1H), 8.17 (d, J = 7.8 Hz, 2H), 7.83 (dd, J = 7.7, 1.8 Hz, 1H), 7.53 (dd, J = 7.7, 4.7 Hz, 1H), 7.43 (t, J = 8.2 Hz, 2H), 7.34 (t, J = 7.9 Hz, 2H), 7.06 (d, J = 8.1 Hz, 2H). $^{13}C$ NMR (101 MHz, $CDCl_3$) δ 150.1, 143.9, 140.7, 139.6, 134.8, 126.3, 123.9, 123.8, 120.7, 120.7, 109.9. HRMS (ESI): m/z calcd for $C_{17}H_{12}BrN_2^+$ $[M+H]^+$ 323.0178, found 323.0187.

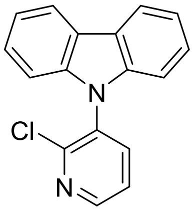

chloro-**4PCz**

*9-(2-Chloropyridin-3-yl)-9H-carbazole (chloro-**4PCz**).* Compound chloro-**4PCz** was prepared according to GP1-A starting from carbazole (502 mg, 3.00 mmol) and 2-chloro-3-fluoropyridine (399 mg, 3.03 mmol) with $Cs_2CO_3$ (1.08 g, 3.30 mmol). Purification by column chromatography (light petroleum/$CH_2Cl_2$ 40% - 60%) gave chloro-**4PCz** (580 mg, 2.08 mmol, 69%) as white solid. $^1$H NMR (400 MHz, $CDCl_3$) δ 8.61 (dd, J = 4.8, 1.8 Hz, 1H), 8.17 (d, J = 7.7 Hz, 2H), 7.89 (dd, J = 7.7, 1.8 Hz, 1H), 7.50 (dd, J = 7.7, 4.8 Hz, 1H), 7.44 (t, J = 8.3 Hz, 2H), 7.34 (t, J = 7.9 Hz, 2H), 7.08 (d, J = 8.1 Hz, 2H). $^{13}$C NMR (101 MHz, $CDCl_3$) δ 151.1, 149.6, 140.6, 139.7, 132.4, 126.3, 123.8, 123.6, 120.7, 120.6, 109.9. HRMS (ESI): m/z calcd for $C_{17}H_{12}ClN_2^+$ $[M+H]^+$ 279.0684, found 279.0697.

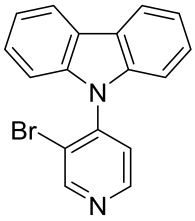

bromo-**5PCz**

*9-(3-Bromopyridin-4-yl)-9H-carbazole (bromo-**5PCz**).* Compound bromo-**5PCz** was prepared according to GP1-A starting from carbazole (502 mg, 3.00 mmol) and 3-bromo-4-chloropyridine (579 mg, 3.01 mmol) with $Cs_2CO_3$ (1.08 g, 3.30 mmol). Purification by column chromatography (light petroleum/$CH_2Cl_2$ 40% - 75%) gave bromo-**5PCz** (669 mg, 2.07 mmol, 69%) as off-white solid. $^1$H NMR (400 MHz, $CDCl_3$) δ 9.07 (s, 1H), 8.75 (d, J = 5.1 Hz, 1H), 8.16 (d, J = 7.4 Hz, 2H), 7.48 (d, J = 5.1 Hz, 1H), 7.44 (t, J = 7.8 Hz, 2H), 7.35 (t, J = 7.9 Hz, 2H), 7.13 (d, J = 8.1 Hz, 2H). $^{13}$C NMR (101 MHz, $CDCl_3$) δ 154.5, 149.9, 145.0, 139.8, 126.4, 125.3, 124.0, 121.0, 121.0, 120.7, 110.3. HRMS (ESI): m/z calcd for $C_{17}H_{12}BrN_2^+$ $[M+H]^+$ 323.0178, found 323.0179.

Compound bromo-**5PCz** was prepared according to GP2 starting from 4-amino-3-bromopyridine (2.60 g, 15.0 mmol) and 2,5-dimethoxytetrahydrofuran (7.93 g, 60.0 mmol). During the reaction additional 2,5-dimethoxytetrahydrofuran (11.89 g, 90.0 mmol) was added stepwise and the reaction was refluxed for 120 h. Purification by column chromatography (light petroleum/$CH_2Cl_2$ 40% - 60%) and recrystallization from EtOH gave bromo-**5PCz** (1.53 g, 4.73 mmol, 32%) as white crystals. $^1$H NMR according to the preparation following GP1-A.

Compound bromo-**5PCz** was prepared according to GP3 starting from 4-amino-3-bromopyridine (865 mg, 5.00 mmol) and 2,2′-dibromo-1,1′-biphenyl (2.03 g, 5.00 mmol) with NaO$^t$Bu (2.88 g, 30.00 mmol), $Pd_2(dba)_3$ (92 mg, 0.10 mmol) and 1,1′-bis(diphenylphosphino)ferrocene (111 mg, 0.20 mmol). Purification by column chromatography (light petroleum/$CH_2Cl_2$ 1:1) gave bromo-**5PCz** (1.32 g, 4.08 mmol, 81%) as white solid. $^1$H NMR according to the preparation following GP1-A.

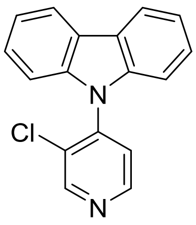

chloro-**5PCz**

*9-(3-Chloropyridin-4-yl)-9H-carbazole (chloro-**5PCz**).* Compound chloro-**5PCz** was prepared according to GP1-A starting from carbazole (502 mg, 3.00 mmol) and 3-chloro-4-fluoropyridine (395 mg, 3.00 mmol) with $Cs_2CO_3$ (1.08 g, 3.30 mmol). Purification by column chromatography (light petroleum/$CH_2Cl_2$ 50% - 60%) gave chloro-**5PCz** (795 mg, 2.85 mmol, 95%) as white solid. $^1$H NMR (400 MHz, $CDCl_3$) δ 8.93 (s, 1H), 8.72 (d, J = 5.1 Hz, 1H), 8.16 (d, J = 7.7 Hz, 2H), 7.51 (d, J = 5.1 Hz, 1H), 7.44 (t, J = 8.3 Hz, 2H), 7.35 (t, J = 7.9 Hz, 2H), 7.16 (d, J = 8.2 Hz, 2H). $^{13}$C NMR (101 MHz, $CDCl_3$) δ 152.2, 149.4, 143.1, 139.8, 130.4, 126.4, 124.6, 124.1, 121.1, 120.7, 110.3. HRMS (ESI): m/z calcd for $C_{17}H_{12}ClN_2^+$ $[M+H]^+$ 279.0684, found 279.0687.

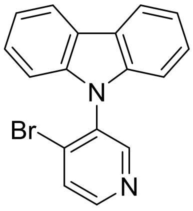

bromo-**6PCz**

*9-(4-Bromopyridin-3-yl)-9H-carbazole (bromo-**6PCz**).* Compound bromo-**6PCz** was prepared according to GP3 starting from 3-amino-4-bromopyridine (87 mg, 0.50 mmol) and 2,2′-diiodo-1,1′-biphenyl (203 mg, 0.50 mmol) with NaO$^t$Bu (288 mg, 3.00 mmol), $Pd_2(dba)_3$ (9 mg, 0.01 mmol) and 1,1′-bis(diphenylphosphino)ferrocene (11 mg, 0.02 mmol). Purification by column chromatography (light petroleum/$CH_2Cl_2$ 50%) gave bromo-**6PCz** (108 mg, 0.33 mmol, 67%) as brown solid. $^1$H NMR (400 MHz, $CDCl_3$) δ 8.74 (s, 1H), 8.61 (d, J = 4.4 Hz, 1H), 8.17 (d, J = 8.1 Hz, 2H), 7.85 (d, J = 5.0 Hz, 1H), 7.43 (t, J = 8.3 Hz, 2H), 7.34 (t, J = 7.9 Hz, 2H), 7.07 (d, J = 8.1 Hz, 2H). $^{13}$C NMR (101 MHz, $CDCl_3$) δ 151.9, 150.3, 140.8, 134.4, 134.4, 129.1, 126.4, 123.7, 120.7, 120.7, 109.9. HRMS (ESI): m/z calcd for $C_{17}H_{12}BrN_2^+$ $[M+H]^+$ 323.0178, found 323.0181.

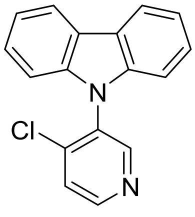

chloro-**6PCz**

*9-(4-Chloropyridin-3-yl)-9H-carbazole (chloro-**6PCz**).* Compound chloro-**6PCz** was prepared according to GP1-A starting from carbazole (502 mg, 3.00 mmol) and 4-chloro-3-fluoropyridine (397 mg, 3.02 mmol) with $Cs_2CO_3$ (1.08 g, 3.30 mmol). Purification by column chromatography (light petroleum/$CH_2Cl_2$ 50%) gave chloro-**6PCz** (476 mg, 1.71 mmol, 57%) as colorless oil, which crystallized after several weeks. $^1$H NMR (400 MHz, $CDCl_3$) δ 8.78 (s, 1H), 8.70 (d, J = 5.3 Hz, 1H), 8.17 (d, J = 7.7 Hz, 2H), 7.66 (d, J = 5.3 Hz, 1H), 7.44 (t, J = 7.7 Hz, 2H), 7.34 (t, J = 7.9 Hz, 2H), 7.09 (d, J = 8.1 Hz, 2H). $^{13}$C NMR (101

MHz, $CDCl_3$) δ 152.0, 150.4, 143.7, 140.8, 132.5, 126.4, 125.8, 123.8, 120.8, 120.7, 109.8. HRMS (ESI): m/z calcd for $C_{17}H_{12}ClN_2^+$ $[M+H]^+$ 279.0684, found 279.0687.

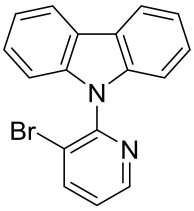

bromo-**7PCz**

*9-(3-Bromopyridin-2-yl)-9H-carbazole (bromo-**7PCz**).* Compound bromo-**7PCz** was prepared according to GP1-A starting from carbazole (836 mg, 5.00 mmol) and 3-bromo-2-chloropyridine (968 mg, 5.03 mmol) with $Cs_2CO_3$ (1.79 g, 5.50 mmol). Purification by column chromatography (light petroleum/$CH_2Cl_2$ 40%) gave bromo-**7PCz** (593 mg, 1.83 mmol, 37%) as colorless oil. $^1$H NMR (400 MHz, $CD_2Cl_2$) δ 8.69 (dd, J = 4.6, 1.6 Hz, 1H), 8.24 (dd, J = 8.0, 1.6 Hz, 1H), 8.16 (d, J = 7.4 Hz, 2H), 7.43 (ddd, J = 8.3, 7.3, 1.2 Hz, 2H), 7.39 (dd, J = 8.0, 4.6 Hz, 1H), 7.33 (t, J = 8.0 Hz, 2H), 7.19 (d, J = 8.2 Hz, 2H). $^{13}$C NMR (101 MHz, $CD_2Cl_2$) δ 149.9, 149.4, 143.9, 140.5, 126.5, 125.4, 124.2, 121.1, 120.9, 119.9, 111.3. HRMS (ESI): m/z calcd for $C_{17}H_{12}BrN_2^+$ $[M+H]^+$ 323.0178, found 323.0178.

Compound bromo-**7PCz** was prepared according to GP2 starting from 2-amino-3-bromopyridine (1.73 g, 10.0 mmol) and 2,5-dimethoxytetrahydrofuran (5.29 g, 40.0 mmol). During the reaction additional 2,5-dimethoxytetrahydrofuran (7.93 g, 60.0 mmol) was added and the reaction was stopped after refluxing for 68 h. Purification by column chromatography (light petroleum/$CH_2Cl_2$ 40% - 60%) gave bromo-**7PCz** (1.25 g, 3.87 mmol, 39%; including minor impurities) as brown oil. $^1$H NMR according to the preparation following GP1-A.

Compound bromo-**7PCz** was prepared according to GP3 starting from 2-amino-3-bromopyridine (554 mg, 3.20 mmol) and 2,2′-diiodo-1,1′-biphenyl (1.30 g, 3.20 mmol) with NaO$^t$Bu (1.83 g, 19.0 mmol), $Pd_2(dba)_3$ (59 mg, 0.06 mmol) and 1,1′-bis(diphenylphosphino)ferrocene (71 mg, 0.12 mmol). Purification by column

chromatography (light petroleum/$CH_2Cl_2$ 40% - 60%) gave bromo-**7PCz** (37 mg, 0.11 mmol, 4%) as colorless oil. $^1$H NMR according to the preparation following GP1-A.

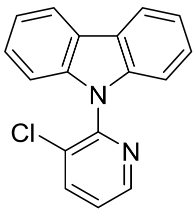

chloro-**7PCz**

*9-(3-Chloropyridin-2-yl)-9H-carbazole (chloro-**7PCz**).* Compound chloro-**7PCz** was prepared according to GP1-A starting from carbazole (836 mg, 5.00 mmol) and 3-chloro-2-fluoropyridine (660 mg, 5.02 mmol) with $Cs_2CO_3$ (1.79 g, 5.50 mmol). Purification by column chromatography (light petroleum/$CH_2Cl_2$ 40%) gave chloro-**7PCz** (1.31 g, 4.70 mmol, 94%) as colorless oil. $^1$H NMR (400 MHz, $CD_2Cl_2$) δ 8.65 (dd, J = 4.6, 1.5 Hz, 1H), 8.17 (d, J = 7.7 Hz, 2H), 8.06 (dd, J = 8.1, 1.5 Hz, 1H), 7.48 – 7.41 (m, 3H), 7.34 (t, J = 7.5 Hz, 2H), 7.23 (d, J = 8.2 Hz, 2H). $^{13}$C NMR (101 MHz, $CD_2Cl_2$) δ 148.7, 148.6, 140.6, 140.5, 130.0, 126.5, 125.1, 124.3, 121.2, 120.8, 111.4. HRMS (ESI): m/z calcd for $C_{17}H_{12}ClN_2^+$ $[M+H]^+$ 279.0684, found 279.0686.

**General procedure for the nucleophilic aromatic substitution reactions towards carboline precursors (GP1-B).** The corresponding carboline (1 eq.) and $Cs_2CO_3$ (2 eq.) were placed in a glass vial and flushed with argon. DMF (2 ml/mmol) and 1-bromo-2-fluorobenzene (2 eq.) were added and the reaction was stirred at 130 °C for 16 h. After cooling, the reaction mixture was poured into water and repeatedly extracted with $CH_2Cl_2$. The organic phases were dried over anhydrous $Na_2SO_4$ and concentrated under reduced pressure. The crude product was purified by column chromatography.

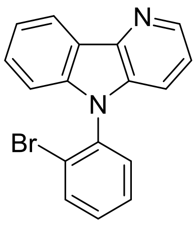

**4PCb**

*5-(2-Bromophenyl)-5H-pyrido[3,2-b]indole (**4PCb**).* Compound **4PCb** was prepared according to GP1-B starting from 5*H*-pyrido[3,2-*b*]indole (841 mg, 5.00 mmol) and 1-bromo-2-fluorobenzene (1.75 g, 10.0 mmol) with $Cs_2CO_3$ (3.26 g, 10.0 mmol). Purification by column chromatography ($CH_2Cl_2$/MeOH 0% - 1%) gave **4PCb** (1.39 g, 4.30 mmol, 86%) as light brown oil. $^1$H NMR (400 MHz, $CDCl_3$) δ 8.62 (d, J = 4.6 Hz, 1H), 8.47 (d, J = 7.8 Hz, 1H), 7.87 (d, J = 7.8 Hz, 1H), 7.58 – 7.35 (m, 6H), 7.32 (dd, J = 8.2, 4.7 Hz, 1H), 7.13 (d, J = 8.2 Hz, 1H). $^{13}$C NMR (101 MHz, $CDCl_3$) δ 142.6, 142.2, 141.8, 136.0, 134.5, 134.5, 131.0, 130.7, 129.1, 128.2, 123.7, 122.4, 121.1, 121.1, 120.3, 117.4, 110.4. HRMS (ESI): m/z calcd for $C_{17}H_{12}BrN_2^+$ $[M+H]^+$ 323.0178, found 323.0179.

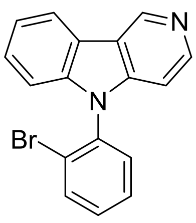

**5PCb**

*5-(2-Bromophenyl)-5H-pyrido[4,3-b]indole (**5PCb**).* Compound **5PCb** was prepared according to GP1-B starting from 5*H*-pyrido[4,3-*b*]indole (842 mg, 5.01 mmol) and 1-bromo-2-fluorobenzene (1.75 g, 10.0 mmol) with $Cs_2CO_3$ (3.26 g, 10.0 mmol). Purification by column chromatography ($CH_2Cl_2$/MeOH 1% - 3%) gave **5PCb** (1.33 g, 4.11 mmol, 82%) as brown oil. $^1$H NMR (400 MHz, $CDCl_3$) δ 9.39 (s, 1H), 8.51 (d, J = 5.8 Hz, 1H), 8.21 (d, J = 7.8 Hz, 1H), 7.87 (dd, J = 8.4, 1.4 Hz, 1H), 7.56 (ddd, J = 8.0, 7.1, 1.5 Hz, 1H), 7.51 – 7.43 (m, 3H), 7.39 (t, J = 8.0 Hz, 1H), 7.11 (d, J = 8.1 Hz, 1H), 7.00 (dd, J = 5.8, 0.9 Hz, 1H). $^{13}$C NMR (101 MHz, $CDCl_3$) δ 145.1, 145.1, 142.7, 141.0, 135.5, 134.5, 131.0, 130.8, 129.1,

127.3, 123.5, 121.7, 121.6, 120.9, 120.2, 110.6, 105.6. HRMS (ESI): m/z calcd for $C_{17}H_{12}BrN_2^+$ [M+H]$^+$ 323.0178, found 323.0179.

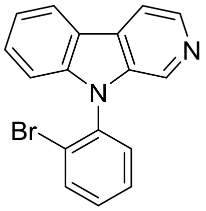

**6PCb**

*9-(2-Bromophenyl)-9H-pyrido[3,4-b]indole (**6PCb**).* Compound **6PCb** was prepared according to GP1-B starting from 9*H*-pyrido[3,4-*b*]indole (505 mg, 3.00 mmol) and 1-bromo-2-fluorobenzene (1.05 g, 6.00 mmol) with $Cs_2CO_3$ (1.96 g, 6.00 mmol). Purification by column chromatography ($CH_2Cl_2$/$Et_2O$ 1% - 3%) gave **6PCb** (894 mg, 2.77 mmol, 92%) as orange oil which crystallized after several days. $^1H$ NMR (400 MHz, $CDCl_3$) δ 8.56 – 8.52 (m, 2H), 8.21 (d, J = 7.9 Hz, 1H), 8.03 (dd, J = 5.3, 1.0 Hz, 1H), 7.88 (dd, J = 8.0, 1.3 Hz, 1H), 7.59 – 7.49 (m, 3H), 7.46 (ddd, J = 8.0, 7.2, 1.9 Hz, 1H), 7.36 (ddd, J = 8.0, 7.2, 0.9 Hz, 1H), 7.15 (d, J = 8.3 Hz, 1H). $^{13}C$ NMR (101 MHz, $CDCl_3$) δ 141.7, 140.2, 137.0, 135.9, 134.6, 133.5, 130.9, 130.8, 129.1, 129.0, 128.8, 123.6, 122.0, 121.6, 120.8, 114.7, 110.9. HRMS (ESI): m/z calcd for $C_{17}H_{12}BrN_2^+$ [M+H]$^+$ 323.0178, found 323.0182.

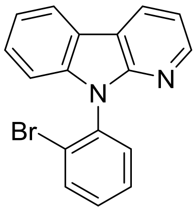

**7PCb**

*9-(2-Bromophenyl)-9H-pyrido[2,3-b]indole (**7PCb**).* Compound **7PCb** was prepared according to GP1-B starting from 9*H*-pyrido[2,3-*b*]indole (506 mg, 3.01 mmol) and 1-bromo-2-fluorobenzene (1.05 g, 6.00 mmol) with $Cs_2CO_3$ (1.96 g, 6.00 mmol). After 16 h additional 1-bromo-2-fluorobenzene (1.05 g, 6.00 mmol) was added and the reaction was stopped after 48 h. Purification by column chromatography (light petroleum/$CH_2Cl_2$ 80% - 100%) gave **7PCb** (331 mg, 1.02 mmol, 34%) as colorless oil which crystallized after several days. $^1H$

NMR (400 MHz, $CD_2Cl_2$) δ 8.46 – 8.41 (m, 2H), 8.17 (d, J = 8.1 Hz, 1H), 7.88 (dd, J = 8.1, 1.1 Hz, 1H), 7.62 – 7.52 (m, 2H), 7.51 – 7.44 (m, 2H), 7.36 (t, J = 8.0 Hz, 1H), 7.26 (dd, J = 7.3, 5.2 Hz, 1H), 7.12 (d, J = 8.2 Hz, 1H). $^{13}C$ NMR (101 MHz, $CD_2Cl_2$) δ 152.7, 147.1, 140.6, 136.3, 134.5, 132.1, 131.0, 129.3, 128.9, 127.5, 124.5, 121.6, 121.3, 121.3, 116.7, 116.7, 111.0. HRMS (ESI): m/z calcd for $C_{17}H_{12}BrN_2^+$ $[M+H]^+$ 323.0178, found 323.0178.

**General procedure for the N-oxide preparation (GP4).** The corresponding PCb (1 eq.) was dissolved in $CH_2Cl_2$ (2 ml/mmol) and cooled to 0 °C. mCPBA (4 eq.) was added portionwise and the reaction was slowly warmed to room temperature and stirred overnight. The reaction mixture was diluted with $CH_2Cl_2$ and washed with 2N NaOH. The organic phase was dried over anhydrous $Na_2SO_4$ and concentrated under reduced pressure. The crude product was purified by column chromatography.

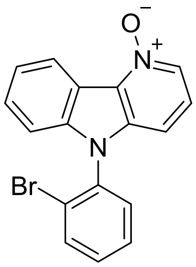


**4PCb-Ox**

*5-(2-Bromophenyl)-5H-pyrido[3,2-b]indole 1-oxide (**4PCb-Ox**).* Compound **4PCb-Ox** was prepared according to GP4 starting from **4PCb** (729 mg, 2.26 mmol) with mCPBA (1.55 g, 8.98 mmol). Purification by column chromatography ($CH_2Cl_2$/MeOH 1% - 2%) gave **4PCb-Ox** (663 mg, 1.95 mmol, 87%) as light brown solid. $^1H$ NMR (400 MHz, $CD_2Cl_2$) δ 8.88 (d, J = 7.9 Hz, 1H), 8.19 (d, J = 6.3 Hz, 1H), 7.90 (dd, J = 8.0, 1.1 Hz, 1H), 7.63 – 7.48 (m, 4H), 7.41 (t, J = 8.0 Hz, 1H), 7.24 (dd, J = 8.3, 6.4 Hz, 1H), 7.10 (d, J = 8.3 Hz, 1H), 6.97 (d, J = 8.3 Hz, 1H). $^{13}C$ NMR (101 MHz, $CD_2Cl_2$) δ 140.9, 138.2, 135.5, 135.0, 132.6, 131.8, 131.7, 131.5, 129.8, 129.1, 124.0, 123.9, 122.0, 121.8, 118.1, 110.3, 108.0. HRMS (ESI): m/z calcd for $C_{17}H_{12}BrN_2O^+$ $[M+H]^+$ 339.0128, found 339.0129.

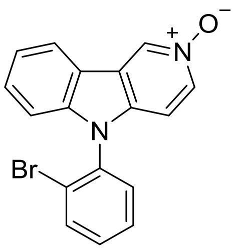

**5PCb-Ox**

*5-(2-Bromophenyl)-5H-pyrido[4,3-b]indole 2-oxide (**5PCb-Ox**).* Compound **5PCb-Ox** was prepared according to GP4 starting from **5PCb** (802 mg, 2.48 mmol) with mCPBA (1.71 g, 9.91 mmol). Purification by column chromatography ($CH_2Cl_2$/MeOH 1% - 4%) gave **5PCb-Ox** (512 mg, 1.51 mmol, 61%) as light brown solid. $^1$H NMR (400 MHz, $CDCl_3$) δ 9.02 (s, 1H), 8.23 (dd, J = 7.0, 1.3 Hz, 1H), 8.06 (d, J = 7.9 Hz, 1H), 7.86 (d, J = 7.8 Hz, 1H), 7.57 (t, J = 7.6 Hz, 1H), 7.53 – 7.45 (m, 3H), 7.37 (t, J = 7.5 Hz, 1H), 7.08 (d, J = 8.3 Hz, 1H), 6.94 (d, J = 7.1 Hz, 1H). $^{13}$C NMR (101 MHz, $CDCl_3$) δ 142.4, 137.7, 136.9, 134.8, 134.7, 132.1, 131.4, 130.7, 129.3, 128.7, 123.2, 122.1, 121.4, 121.3, 119.9, 111.1, 107.3. HRMS (ESI): m/z calcd for $C_{17}H_{12}BrN_2O^+$ $[M+H]^+$ 339.0128, found 339.0132.

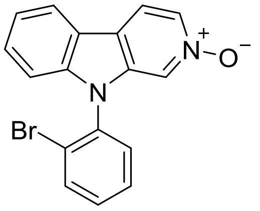

**6PCb-Ox**

*9-(2-Bromophenyl)-9H-pyrido[3,4-b]indole 2-oxide (**6PCb-Ox**).* Compound **6PCb-Ox** was prepared according to GP4 starting from **6PCb** (488 mg, 1.51 mmol) with mCPBA (1.04 g, 6.03 mmol). Purification by column chromatography ($CH_2Cl_2$/MeOH 1% - 3%) gave **6PCb-Ox** (463 mg, 1.37 mmol, 90%) as light brown foam. $^1$H NMR (400 MHz, $CDCl_3$) δ 8.18 (dd, J = 6.7, 1.5 Hz, 1H), 8.14 (d, J = 0.9 Hz, 1H), 8.07 (d, J = 7.9 Hz, 1H), 7.91 (d, J = 6.7 Hz, 1H), 7.86 (dd, J = 7.5, 2.3 Hz, 1H), 7.56 (ddd, J = 8.1, 7.1, 1.7 Hz, 1H), 7.52 – 7.44 (m, 3H), 7.36 (t, J = 7.5 Hz, 1H), 7.09 (d, J = 8.2 Hz, 1H). $^{13}$C NMR (101 MHz, $CDCl_3$) δ 142.8, 138.2, 134.7, 134.7, 132.5, 131.5, 130.7, 129.3, 128.4, 123.7, 123.3, 121.9, 121.5, 121.3, 121.2, 116.5, 111.0. HRMS (ESI): m/z calcd for $C_{17}H_{12}BrN_2O^+$ $[M+H]^+$ 339.0128, found 339.0130.

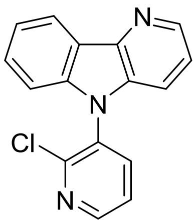

**4,12PyCb**

*5-(2-Chloropyridin-3-yl)-5H-pyrido[3,2-b]indole (**4,12PyCb**).* Sodium hydride (144 mg, 6.00 mmol, 2 eq.) was added in portions to a stirred solution of 5*H*-pyrido[3,2-*b*]indole (505 mg, 3.00 mmol, 1 eq.) in 6 ml DMF under argon atmosphere. The solution was heated to 40 °C and stirred for 1 h. 2-Chloro-3-fluoropyridine (592 mg, 4.50 mmol, 1.5 eq.) was added and the reaction was stirred at 50 °C for 16 h. After cooling, the reaction mixture was quenched with water and repeatedly extracted with $CH_2Cl_2$. The organic phases were dried over anhydrous $Na_2SO_4$ and concentrated under reduced pressure. Column chromatography ($CH_2Cl_2$/MeOH 1%) gave **4,12PyCb** (749 mg, 2.68 mmol, 89%) as white solid. $^1$H NMR (400 MHz, $CDCl_3$) δ 8.67 – 8.60 (m, 2H), 8.46 (d, J = 7.7 Hz, 1H), 7.90 (dd, J = 7.8, 1.8 Hz, 1H), 7.56 – 7.49 (m, 2H), 7.45 – 7.37 (m, 2H), 7.34 (dd, J = 8.3, 4.6 Hz, 1H), 7.13 (d, J = 8.2 Hz, 1H). $^{13}$C NMR (101 MHz, $CDCl_3$) δ 150.8, 150.0, 143.2, 142.7, 141.4, 139.5, 134.1, 131.5, 128.4, 123.7, 122.9, 121.7, 121.2, 120.5, 117.1, 110.1. HRMS (ESI): m/z calcd for $C_{16}H_{11}ClN_3^+$ $[M+H]^+$ 280.0636, found 280.0637.

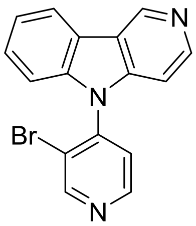

**5,11PyCb**

*5-(3-Bromopyridin-4-yl)-5H-pyrido[4,3-b]indole (**5,11PyCb**).* Compound **5,11PyCb** was prepared according to GP1-A starting from 5*H*-pyrido[4,3-*b*]indole (336 mg, 2.00 mmol) and 3-bromo-4-chloropyridine (385 mg, 2.00 mmol) with $Cs_2CO_3$ (717 mg, 2.20 mmol). Purification by column chromatography ($CH_2Cl_2$/MeOH 1% - 3%) gave **5,11PyCb** (246 mg, 0.76 mmol, 38%) as red solid. $^1$H NMR (400 MHz, $CDCl_3$) δ 9.40 (s, 1H), 9.08 (s, 1H), 8.79

(d, J = 5.1 Hz, 1H), 8.55 (d, J = 5.7 Hz, 1H), 8.22 (d, J = 7.8 Hz, 1H), 7.53 – 7.45 (m, 2H), 7.42 (t, J = 7.6 Hz, 1H), 7.15 (d, J = 8.1 Hz, 1H), 7.03 (dd, J = 5.7, 0.8 Hz, 1H). $^{13}$C NMR (101 MHz, $CDCl_3$) δ 154.8, 150.3, 145.8, 144.1, 143.6, 143.3, 139.8, 127.5, 124.9, 122.4, 122.1, 121.1, 120.7, 120.6, 110.6, 105.6. HRMS (ESI): m/z calcd for $C_{16}H_{11}BrN_3^+$ $[M+H]^+$ 324.0131, found 324.0132.

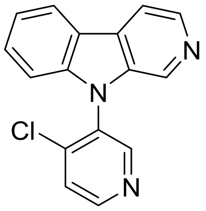

**6,10PyCb**

*9-(4-Chloropyridin-3-yl)-9H-pyrido[3,4-b]indole (**6,10PyCb**).* Compound **6,10PyCb** was prepared according to GP1-A starting from 9*H*-pyrido[3,4-*b*]indole (288 mg, 1.71 mmol) and 4-chloro-3-fluoropyridine hydrochloride (287 mg, 1.71 mmol) with $Cs_2CO_3$ (1.11 g, 3.42 mmol). Purification by column chromatography ($CH_2Cl_2$/MeOH 1% - 2%) gave **6,10PyCb** (231 mg, 0.83 mmol, 49%) as light brown solid. $^1$H NMR (400 MHz, $CDCl_3$) δ 8.79 (s, 1H), 8.73 (d, J = 5.3 Hz, 1H), 8.57 (d, J = 5.3 Hz, 1H), 8.54 (d, J = 0.8 Hz, 1H), 8.21 (d, J = 7.6 Hz, 1H), 8.03 (dd, J = 5.3, 1.0 Hz, 1H), 7.68 (d, J = 5.3 Hz, 1H), 7.56 (ddd, J = 8.4, 7.2, 1.2 Hz, 1H), 7.39 (t, J = 8.0 Hz, 1H), 7.15 (d, J = 8.3 Hz, 1H). $^{13}$C NMR (101 MHz, $CDCl_3$) δ 151.6, 151.0, 143.4, 141.6, 140.8, 136.9, 133.0, 131.6, 129.6, 129.1, 125.9, 122.1, 122.0, 121.5, 114.9, 110.5. HRMS (ESI): m/z calcd for $C_{16}H_{11}ClN_3^+$ $[M+H]^+$ 280.0636, found 280.0638.

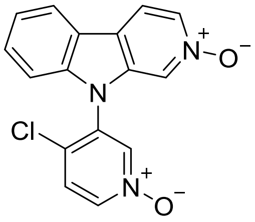

**6,10PyCb-Ox**

*9-(4-Chloro-1-oxidopyridin-3-yl)-9H-pyrido[3,4-b]indole 2-oxide (**6,10PyCb-Ox**).* Compound **6,10PyCb-Ox** was prepared according to GP4 with double amount of oxidant starting from **6,10PyCb** (356 mg, 1.27 mmol) with mCPBA (1.73 g, 10.02 mmol). After 20 h

additional mCPBA (431 mg, 2.50 mmol) was added and the reaction was stirred overnight again. After standard work up purification by column chromatography ($CH_2Cl_2$/MeOH 5% - 10%) gave **6,10PyCb-Ox** (202 mg, 0.65 mmol, 51%) as light brown solid. $^1$H NMR (400 MHz, DMSO) δ 8.84 (d, J = 2.0 Hz, 1H), 8.66 (d, J = 0.9 Hz, 1H), 8.47 (dd, J = 7.1, 2.1 Hz, 1H), 8.26 (dd, J = 9.2, 7.4 Hz, 2H), 8.17 (dd, J = 6.7, 1.5 Hz, 1H), 7.89 (d, J = 7.1 Hz, 1H), 7.52 (t, J = 7.7 Hz, 1H), 7.39 (t, J = 7.5 Hz, 1H), 7.32 (d, J = 8.2 Hz, 1H). $^{13}$C NMR (101 MHz, DMSO) δ 141.7, 141.3, 141.1, 138.0, 132.8, 132.2, 129.4, 128.1, 127.8, 123.4, 122.0, 121.5, 121.3, 119.9, 117.2, 110.7. HRMS (ESI): m/z calcd for $C_{16}H_{11}ClN_3O_2^+$ [M+H]$^+$ 312.0534, found 312.0538.

## 2.2 Synthesis of mono substituted NICzs

**General procedure for the ring closing C-H activation reactions (GP5).** A glass vial was charged with the corresponding halogenated precursor (1 eq.), $K_2CO_3$ (2 eq.) and (NHC)Pd(allyl)Cl (5 mol%) and flushed with argon. After addition of 10 ml/mmol degassed DMA, the reaction was stirred under argon atmosphere until full conversion at 130 °C (4 h – 8 h). After cooling, the reaction mixture was poured into water and repeatedly extracted with $CH_2Cl_2$. The organic phases were dried over anhydrous $Na_2SO_4$ and concentrated under reduced pressure. The crude product was purified by column chromatography.

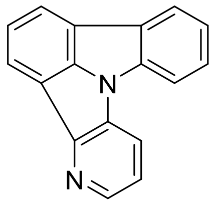

**4NICz**

*Pyrido[2',3':4,5]pyrrolo[3,2,1-jk]carbazole (**4NICz**).* Compound **4NICz** was prepared according to GP5 starting from bromo-**4PCz** (323 mg, 1.00 mmol) with $K_2CO_3$ (278 mg, 2.01 mmol) and (NHC)Pd(allyl)Cl (29 mg, 0.05 mmol) in 10 ml DMA. Purification by column chromatography ($CH_2Cl_2$/MeOH 0% - 2%) gave **4NICz** (224 mg, 0.93 mmol, 93%) as off-white solid. $^1$H NMR (400 MHz, $CDCl_3$) δ 8.60 (dd, J = 4.9, 1.2 Hz, 1H), 8.28 (d, J = 7.5 Hz,

1H), 8.14 – 8.06 (m, 3H), 7.80 (d, J = 8.0 Hz, 1H), 7.65 (t, J = 7.4 Hz, 1H), 7.54 (t, J = 8.2 Hz, 1H), 7.41 (dd, J = 8.3, 4.9 Hz, 1H), 7.37 (t, J = 7.6 Hz, 1H). $^{13}$C NMR (101 MHz, $CDCl_3$) δ 148.3, 144.0, 143.1, 138.8, 133.1, 129.7, 127.2, 123.8, 123.4, 122.5, 121.3, 120.5, 120.4, 119.2, 118.7, 117.6, 112.3. HRMS (ESI): m/z calcd for $C_{17}H_{11}N_2^+$ $[M+H]^+$ 243.0917, found 243.0921.

Compound **4NICz** was prepared according to GP5 starting from chloro-**4PCz** (279 mg, 1.00 mmol) with $K_2CO_3$ (279 mg, 2.02 mmol) and (NHC)Pd(allyl)Cl (29 mg, 0.05 mmol) in 10 ml DMA. Purification by column chromatography ($CH_2Cl_2$/MeOH 0% - 2%) gave **4NICz** (238 mg, 0.98 mmol, 98%) as off-white solid. $^1$H NMR according to the preparation starting from bromo-**4PCz**.

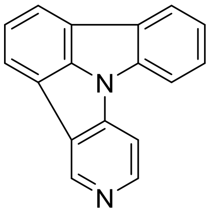

**5NICz**

*Pyrido[3',4':4,5]pyrrolo[3,2,1-jk]carbazole (**5NICz**).* Compound **5NICz** was prepared according to GP5 starting from bromo-**5PCz** (324 mg, 1.00 mmol) with $K_2CO_3$ (277 mg, 2.00 mmol) and (NHC)Pd(allyl)Cl (29 mg, 0.05 mmol) in 10 ml DMA. Purification by column chromatography ($CH_2Cl_2$/MeOH 1%) gave **5NICz** (233 mg, 0.96 mmol, 96%) as white solid. $^1$H NMR (400 MHz, $CDCl_3$) δ 9.26 (s, 1H), 8.65 (d, J = 5.6 Hz, 1H), 8.03 (d, J = 7.8 Hz, 1H), 7.95 (t, J = 7.9 Hz, 2H), 7.74 (d, J = 8.0 Hz, 1H), 7.63 (d, J = 5.6 Hz, 1H), 7.58 – 7.48 (m, 2H), 7.36 (t, J = 8.0 Hz, 1H). $^{13}$C NMR (101 MHz, $CDCl_3$) δ 146.7, 144.7, 143.6, 142.4, 138.2, 130.7, 127.2, 126.1, 124.1, 123.4, 123.0, 120.3, 120.0, 118.8, 116.2, 112.8, 107.5. HRMS (ESI): m/z calcd for $C_{17}H_{11}N_2^+$ $[M+H]^+$ 243.0917, found 243.0922.

Compound **5NICz** was prepared according to GP5 starting from chloro-**5PCz** (280 mg, 1.00 mmol) with $K_2CO_3$ (282 mg, 2.04 mmol) and (NHC)Pd(allyl)Cl (29 mg, 0.05 mmol) in 10 ml

DMA. Purification by column chromatography ($CH_2Cl_2$/MeOH 1%) gave **5NICz** (235 mg, 0.97 mmol, 97%) as white solid. $^1$H NMR according to the preparation starting from bromo-**5PCz**.

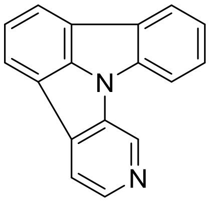

**6NICz**

*Pyrido[4',3':4,5]pyrrolo[3,2,1-jk]carbazole (6NICz).* Compound **6NICz** was prepared according to GP5 starting from bromo-**6PCz** (129 mg, 0.40 mmol) with $K_2CO_3$ (2 eq., 112 mg, 0.81 mmol) and (NHC)Pd(allyl)Cl (5 mol%, 11 mg, 0.02 mmol) in 4 ml DMA. Purification by column chromatography ($CH_2Cl_2$/MeOH 1%) gave **6NICz** (77 mg, 0.32 mmol, 80%) as yellow solid. $^1$H NMR (400 MHz, $CDCl_3$) δ 9.19 (d, J = 0.9 Hz, 1H), 8.57 (d, J = 5.2 Hz, 1H), 8.07 – 8.02 (m, 2H), 7.99 (d, J = 7.5 Hz, 1H), 7.93 (dd, J = 5.2, 1.0 Hz, 1H), 7.79 (d, J = 8.1 Hz, 1H), 7.58 – 7.51 (m, 2H), 7.35 (td, J = 7.7, 1.0 Hz, 1H). $^{13}$C NMR (101 MHz, $CDCl_3$) δ 144.2, 142.3, 138.4, 135.9, 134.8, 134.3, 129.9, 127.4, 123.5, 123.4, 122.5, 121.8, 120.8, 119.4, 117.6, 116.3, 112.6. HRMS (ESI): m/z calcd for $C_{17}H_{11}N_2^+$ $[M+H]^+$ 243.0917, found 243.0920.

Compound **6NICz** was prepared according to GP5 starting from chloro-**6PCz** (281 mg, 1.01 mmol) with $K_2CO_3$ (281 mg, 2.03 mmol) and (NHC)Pd(allyl)Cl (29 mg, 0.05 mmol) in 10 ml DMA. Purification by column chromatography ($CH_2Cl_2$/MeOH 1%) gave **6NICz** (225 mg, 0.93 mmol, 92%) as yellow solid. $^1$H NMR according to the preparation starting from bromo-**6PCz**.

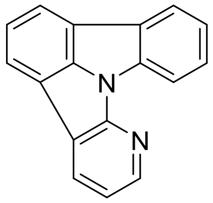

**7NICz**

*Pyrido[3',2':4,5]pyrrolo[3,2,1-jk]carbazole (7NICz).* Compound **7NICz** was prepared according to GP5 starting from bromo-**7PCz** (326 mg, 1.01 mmol) with $K_2CO_3$ (282 mg, 2.04 mmol) and (NHC)Pd(allyl)Cl (29 mg, 0.05 mmol) in 10 ml DMA. Purification by column chromatography (light petroleum/$CH_2Cl_2$ 60%) gave **7NICz** (205 mg, 0.85 mmol, 84%) as white solid. $^1$H NMR (400 MHz, $CD_2Cl_2$) δ 8.52 (dd, J = 5.0, 1.6 Hz, 1H), 8.37 (dd, J = 7.7, 1.6 Hz, 1H), 8.23 (d, J = 8.1 Hz, 1H), 8.13 (d, J = 7.8 Hz, 1H), 8.07 (d, J = 7.4 Hz, 1H), 8.02 (d, J = 7.5 Hz, 1H), 7.63 – 7.56 (m, 2H), 7.41 (td, J = 7.7, 1.0 Hz, 1H), 7.28 (dd, J = 7.7, 5.0 Hz, 1H). $^{13}$C NMR (101 MHz, $CD_2Cl_2$) δ 151.4, 146.5, 143.3, 138.6, 131.2, 130.6, 127.7, 124.0, 123.9, 123.5, 123.2, 120.8, 120.5, 119.5, 117.7, 116.5, 114.3. HRMS (ESI): m/z calcd for $C_{17}H_{11}N_2^+$ $[M+H]^+$ 243.0917, found 243.0921.

Compound **7NICz** was prepared according to GP5 starting from chloro-**7PCz** (281 mg, 1.01 mmol) with $K_2CO_3$ (280 mg, 2.03 mmol) and (NHC)Pd(allyl)Cl (29 mg, 0.05 mmol) in 10 ml DMA. Purification by column chromatography (light petroleum/$CH_2Cl_2$ 60%) gave **7NICz** (235 mg, 0.97 mmol, 96%) as white solid. $^1$H NMR according to the preparation starting from bromo-**7PCz**.

**General procedure for the reduction of the N-oxides (GP6).** The mixture of the N-oxide isomers (1 eq.) and iron powder (2 eq.) were dissolved in AcOH (22 ml/mmol) and stirred at 60°C under argon atmosphere for 2 hours. After cooling, the reaction mixture was poured into cold 2N NaOH and repeatedly extracted with $CH_2Cl_2$. The organic phases were dried over anhydrous $Na_2SO_4$ and concentrated under reduced pressure. The crude product was purified by column chromatography.

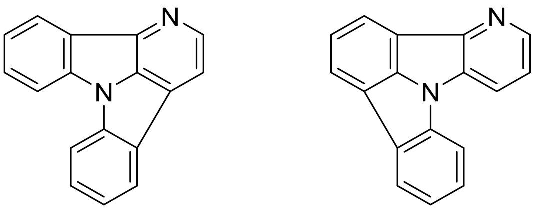

**1NICz** **4NICz**

*Dibenzo[b,e]pyrido[2,3,4-gh]pyrrolizine* ***(1NICz)*** and *pyrido[2',3':4,5]pyrrolo[3,2,1-jk]carbazole* ***(4NICz).*** Compounds **1NICz** and **4NICz** were prepared according to GP5 starting from **4PCb** (323 mg, 1.00 mmol) with $K_2CO_3$ (280 mg, 2.03 mmol) and (NHC)Pd(allyl)Cl (29 mg, 0.05 mmol) in 10 ml DMA. Purification by repeated column chromatography ($CH_2Cl_2$/$Et_2O$ 1%) gave pure **1NICz** (108 mg, 0.45 mmol, 45%) and **4NICz** (37 mg, 0.15 mmol, 15%) as off-white solids. Additional mixed fractions included dehalogenated side product which could not be separated. **1NICz**: $^1$H NMR (400 MHz, $CDCl_3$) δ 8.83 (d, J = 5.1 Hz, 1H), 8.32 (d, J = 7.5 Hz, 1H), 8.06 (d, J = 7.8 Hz, 1H), 7.80 – 7.76 (m, 3H), 7.61 – 7.55 (m, 2H), 7.38 (t, J = 8.1 Hz, 1H), 7.32 (t, J = 8.1 Hz, 1H). $^{13}$C NMR (101 MHz, $CDCl_3$) δ 144.9, 140.4, 140.2, 140.2, 138.4, 129.6, 129.3, 129.2, 128.7, 124.9, 124.6, 123.0, 122.8, 122.4, 113.9, 112.9, 112.7. HRMS (ESI): m/z calcd for $C_{17}H_{11}N_2^+$ $[M+H]^+$ 243.0917, found 243.0919. $^1$H NMR of **4NICz** according to the preparation starting from bromo-**4PCz**.

Compounds **1NICz** and **4NICz** were prepared according to GP5 starting from **4PCb-Ox** (339 mg, 1.00 mmol) with $K_2CO_3$ (281 mg, 2.03 mmol) and (NHC)Pd(allyl)Cl (29 mg, 0.05 mmol) in 10 ml DMA. The crude product was flashed over silica to give a mixture of the formed N-oxide isomers (238 mg, 0.92 mmol, 92%). The N-oxides were then reduced according to GP6 with iron-powder (101 mg, 1.81 mmol) in 20 ml AcOH. Purification by repeated column chromatography ($CH_2Cl_2$/$Et_2O$ 1%) gave pure **1NICz** (141 mg, 0.58 mmol, 58%) and **4NICz** (8 mg, 0.03 mmol, 3%) as off-white solids. According to $^1$H-NMR the formed isomeric NICz mixture (167 mg, 0.69 mmol) contained **1NICz** (0.61 mmol) and **4NICz** (0.07 mmol). $^1$H NMR according to the preparation starting from **4PCb** and bromo-**4PCz**, respectively.

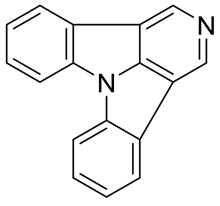

**2NICz**

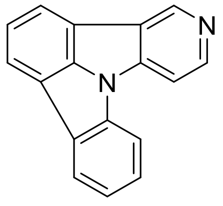

**5NICz**

*Dibenzo[b,e]pyrido[3,4,5-gh]pyrrolizine (2NICz)* and *pyrido[3',4':4,5]pyrrolo[3,2,1-jk]carbazole (5NICz).* Compounds **2NICz** and **5NICz** were prepared according to GP5 starting from **5PCb** (322 mg, 1.00 mmol) with $K_2CO_3$ (279 mg, 2.02 mmol) and (NHC)Pd(allyl)Cl (29 mg, 0.05 mmol) in 10 ml DMA. Purification by repeated column chromatography ($CH_2Cl_2$/MeOH 1% - 2%) gave pure **2NICz** (111 mg, 0.46 mmol, 46%) as off-white solid. According to $^1$H-NMR the formed isomeric NICz mixture (211 mg, 0.87 mmol) contained **2NICz** (0.76 mmol) and **5NICz** (0.11 mmol). **2NICz**: $^1$H NMR (400 MHz, $CDCl_3$) δ 9.15 (s, 2H), 8.09 (d, J = 7.8 Hz, 2H), 7.77 (d, J = 8.1 Hz, 2H), 7.55 (t, J = 7.8 Hz, 2H), 7.37 (t, J = 7.7 Hz, 2H). $^{13}$C NMR (101 MHz, $CDCl_3$) δ 148.1, 139.0, 138.8, 128.2, 127.8, 124.0, 122.9, 115.9, 112.6. HRMS (ESI): m/z calcd for $C_{17}H_{11}N_2^+$ $[M+H]^+$ 243.0917, found 243.0920.

Compounds **2NICz** and **5NICz** were prepared according to GP5 starting from **5PCb-Ox** (339 mg, 1.00 mmol) with $K_2CO_3$ (277 mg, 2.00 mmol) and (NHC)Pd(allyl)Cl (29 mg, 0.05 mmol) in 10 ml DMA. The crude product was flashed over silica to give a mixture of the formed N-oxide isomers (231 mg, 0.89 mmol, 89%). The N-oxides were then reduced according to GP6 with iron-powder (98 mg, 1.76 mmol) in 20 ml AcOH. Purification by repeated column chromatography ($CH_2Cl_2$/MeOH 1% - 2%) gave pure **2NICz** (106 mg, 0.44 mmol, 44%) and **5NICz** (10 mg, 0.04 mmol, 4%) as off-white and white solid, respectively. According to $^1$H-NMR the formed isomeric NICz mixture (184 mg, 0.76 mmol) contained **2NICz** (0.62 mmol) and **5NICz** (0.14 mmol). $^1$H NMR according to the preparation starting from **5PCb** and bromo-**5PCz**, respectively.

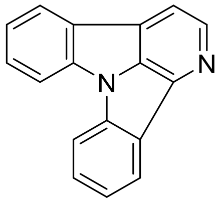

**1NICz**

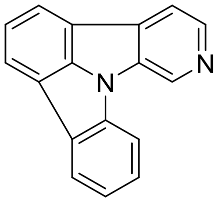

**6NICz**

*Dibenzo[b,e]pyrido[2,3,4-gh]pyrrolizine (**1NICz**) and pyrido[4',3':4,5]pyrrolo[3,2,1-jk]carbazole (**6NICz**).* Compounds **1NICz** and **6NICz** were prepared according to GP5 starting from **6PCb** (324 mg, 1.00 mmol) with $K_2CO_3$ (281 mg, 2.03 mmol) and (NHC)Pd(allyl)Cl (29 mg, 0.05 mmol) in 10 ml DMA. Purification by repeated column chromatography ($CH_2Cl_2/Et_2O$ 1% - 5%) gave pure **1NICz** (38 mg, 0.16 mmol, 16%) and **6NICz** (148 mg, 0.61 mmol, 61%) as off-white and yellow solid, respectively. According to $^1$H-NMR the formed isomeric NICz mixture (225 mg, 0.93 mmol) contained **1NICz** (0.19 mmol) and **6NICz** (0.74 mmol). $^1$H NMR according to the preparation starting from **4PCb** and bromo-**6PCz**, respectively.

Compounds **1NICz** and **6NICz** were prepared according to GP5 starting from **6PCb-Ox** (339 mg, 1.00 mmol) with $K_2CO_3$ (281 mg, 2.03 mmol) and (NHC)Pd(allyl)Cl (29 mg, 0.05 mmol) in 10 ml DMA. The crude product was flashed over silica to give a mixture of the formed N-oxide isomers (232 mg, 0.90 mmol, 90%). The N-oxides were then reduced according to GP6 with iron-powder (98 mg, 1.76 mmol) in 20 ml AcOH. Purification by repeated column chromatography ($CH_2Cl_2/Et_2O$ 1% - 5%) gave pure **1NICz** (138 mg, 0.57 mmol, 57%) and **6NICz** (28 mg, 0.12 mmol, 12%) as off-white and white solids, respectively. According to $^1$H-NMR the formed isomeric NICz mixture (210 mg, 0.87 mmol) contained **1NICz** (0.71 mmol) and **6NICz** (0.16 mmol). $^1$H NMR according to the preparation starting from **4PCb** and bromo-**6PCz**, respectively.

## 2.3 Synthesis of twofold substituted NICzs

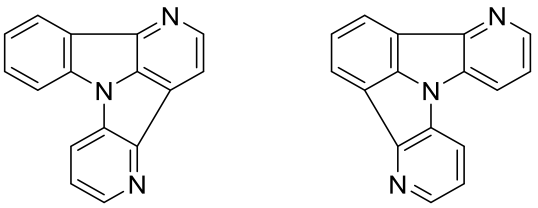


**1,4NICz** **4,12NICz**

*Benzo[b]dipyrido[3,2-e:4',3',2'-gh]pyrrolizine* ***(1,4NICz)*** and *pyrido[3,2-b]pyrido[2',3':4,5]pyrrolo[3,2,1-hi]indole* ***(4,12NICz).*** Compounds **1,4NICz** and **4,12NICz** were prepared according GP5 starting from **4,12PyCb** (280 mg, 1.00 mmol) with $K_2CO_3$ (280 mg, 2.03 mmol) and (NHC)Pd(allyl)Cl (29 mg, 0.05 mmol) in 10 ml DMA. Purification by column chromatography and preparative HPLC ($CH_2Cl_2$/MeOH 1% - 3%) gave **1,4NICz** (98 mg, 0.40 mmol, 40%) and **4,12NICz** (50 mg, 0.21 mmol, 21%) as white solids. According to $^{1}$H-NMR the formed isomeric NICz mixture (228 mg, 0.94 mmol) contained **1,4NICz** (0.65 mmol) and **4,12NICz** (0.28 mmol). **1,4NICz**: $^{1}$H NMR (400 MHz, $CDCl_3$) δ 8.89 (d, J = 5.2 Hz, 1H), 8.58 (dd, J = 4.8, 1.3 Hz, 1H), 8.26 (d, J = 8.2 Hz, 1H), 8.03 – 7.95 (m, 2H), 7.64 (d, J = 8.1 Hz, 1H), 7.53 (t, J = 8.3 Hz, 1H), 7.44 (dd, J = 8.3, 4.8 Hz, 1H), 7.36 (t, J = 8.1 Hz, 1H). $^{13}$C NMR (101 MHz, $CDCl_3$) δ 147.4, 145.6, 144.0, 141.6, 140.3, 138.1, 134.9, 129.2, 129.2, 123.5, 123.5, 123.2, 122.7, 119.7, 114.2, 112.7. HRMS (ESI): m/z calcd for $C_{16}H_{10}N_3^+$ $[M+H]^+$ 244.0869, found 244.0871. **4,12NICz**: $^{1}$H NMR (400 MHz, $CDCl_3$) δ 8.59 (dd, J = 4.9, 1.3 Hz, 2H), 8.30 (d, J = 7.5 Hz, 2H), 7.96 (dd, J = 8.2, 1.3 Hz, 2H), 7.70 (t, J = 7.5 Hz, 1H), 7.38 (dd, J = 8.2, 4.9 Hz, 2H). $^{13}$C NMR (101 MHz, $CDCl_3$) δ 147.9, 144.0, 143.8, 133.1, 124.6, 122.2, 120.8, 118.7, 118.1. HRMS (ESI): m/z calcd for $C_{16}H_{10}N_3^+$ $[M+H]^+$ 244.0869, found 244.0870.

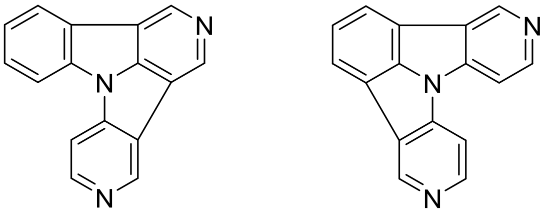


**2,5NICz** **5,11NICz**

*Benzo[b]dipyrido[4,3-e:3',4',5'-gh]pyrrolizine* ***(2,5NICz)*** *and pyrido[4,3-b]pyrido[3',4':4,5]pyrrolo[3,2,1-hi]indole* ***(5,11NICz)****.* Compounds **2,5NICz** and **5,11NICz** were prepared according GP5 starting from **5,11PyCb** (324 mg, 1.00 mmol) with $K_2CO_3$ (278 mg, 2.01 mmol) and (NHC)Pd(allyl)Cl (29 mg, 0.05 mmol) in 10 ml DMA. Purification by column chromatography and preparative HPLC ($CH_2Cl_2$/MeOH 2% - 4%) gave **2,5NICz** (130 mg, 0.53 mmol, 53%) and **5,11NICz** (37 mg, 0.15 mmol, 15%) as white solids. According to $^{1}$H-NMR the formed isomeric NICz mixture (222 mg, 0.91 mmol) contained **2,5NICz** (0.72 mmol) and **5,11NICz** (0.19 mmol). **2,5NICz**: $^{1}$H NMR (400 MHz, $CDCl_3$) δ 9.36 (s, 1H), 9.24 (s, 1H), 9.20 (s, 1H), 8.76 (d, J = 5.6 Hz, 1H), 8.13 (d, J = 7.8 Hz, 1H), 7.83 (d, J = 8.1 Hz, 1H), 7.74 (d, J = 5.6 Hz, 1H), 7.62 (t, J = 7.8 Hz, 1H), 7.46 (t, J = 7.7 Hz, 1H). $^{13}$C NMR (101 MHz, $CDCl_3$) δ 148.2, 148.0, 145.6, 142.9, 139.9, 139.9, 138.4, 128.9, 128.4, 124.5, 124.4, 124.2, 116.2, 113.8, 113.2, 107.9. HRMS (ESI): m/z calcd for $C_{16}H_{10}N_3^+$ $[M+H]^+$ 244.0869, found 244.0871. **5,11NICz**: $^{1}$H NMR (400 MHz, $CDCl_3$) δ 9.33 (s, 2H), 8.74 (d, J = 5.5 Hz, 2H), 8.05 (d, J = 7.5 Hz, 2H), 7.73 (d, J = 5.5 Hz, 2H), 7.66 (t, J = 7.5 Hz, 1H). $^{13}$C NMR (101 MHz, $CDCl_3$) δ 147.4, 145.1, 143.5, 142.4, 126.7, 125.3, 121.1, 116.6, 108.1. HRMS (ESI): m/z calcd for $C_{16}H_{10}N_3^+$ $[M+H]^+$ 244.0869, found 244.0873.

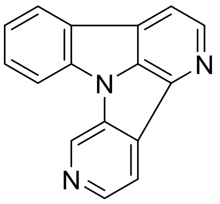


**1,10NICz**

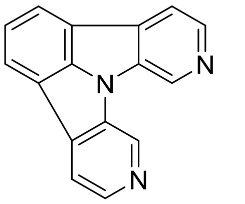


**6,10NICz**

*Benzo[b]dipyrido[3,4-e:2',3',4'-gh]pyrrolizine* ***(1,10NICz)*** and *pyrido[3,4-b]pyrido[4',3':4,5]pyrrolo[3,2,1-hi]indole* ***(6,10NICz)****.* Compounds **1,10NICz** and **6,10NICz** were prepared according GP5 starting from **6,10PyCb** (280 mg, 1.00 mmol) with $K_2CO_3$ (279 mg, 2.02 mmol) and (NHC)Pd(allyl)Cl (29 mg, 0.05 mmol) in 10 ml DMA. Purification by column chromatography ($CH_2Cl_2$/MeOH 1% - 4%) gave **1,10NICz** (11 mg, 0.5 mmol, 5%) and **6,10NICz** (186 mg, 0.76 mmol, 76%) as yellow and light brown solid, respectively.

**1,10NICz**: $^1$H NMR (400 MHz, $CDCl_3$) δ 9.15 (s, 1H), 8.88 (d, J = 5.0 Hz, 1H), 8.63 (d, J = 5.1 Hz, 1H), 8.15 (d, J = 5.1 Hz, 1H), 8.04 (d, J = 7.8 Hz, 1H), 7.84 (d, J = 5.0 Hz, 1H), 7.75 (d, J = 8.1 Hz, 1H), 7.62 (t, J = 7.8 Hz, 1H), 7.35 (t, J = 7.6 Hz, 1H). $^{13}$C NMR (101 MHz, $CDCl_3$) δ 146.3, 143.4, 140.0, 139.2, 138.1, 135.9, 135.7, 134.9, 130.2, 129.1, 125.9, 125.3, 123.2, 117.2, 115.9, 113.4. HRMS (ESI): m/z calcd for $C_{16}H_{10}N_3^+$ $[M+H]^+$ 244.0869, found 244.0872. **6,10NICz**: $^1$H NMR (400 MHz, $CDCl_3$) δ 9.14 (s, 2H), 8.60 (d, J = 5.2 Hz, 2H), 8.09 (d, J = 7.5 Hz, 2H), 7.91 (dd, J = 5.2, 0.9 Hz, 2H), 7.61 (t, J = 7.5 Hz, 1H). $^{13}$C NMR (101 MHz, $CDCl_3$) δ 144.4, 143.1, 135.8, 134.6, 134.5, 124.2, 123.4, 117.7, 117.2. HRMS (ESI): m/z calcd for $C_{16}H_{10}N_3^+$ $[M+H]^+$ 244.0869, found 244.0871.

Compounds **1,10NICz** and **6,10NICz** were prepared according GP5 starting from **6,10PyCb-Ox** (188 mg, 0.60 mmol) with $K_2CO_3$ (2 eq., 168 mg, 1.22 mmol) and (NHC)Pd(allyl)Cl (5 mol%, 17 mg, 0.03 mmol) in 10 ml DMA. The crude product was flashed over silica to give a mixture of the formed N-oxide isomers (96 mg, 0.35 mmol, 58%). The N-oxides were then reduced according to GP6 with iron-powder (4 eq., 78 mg, 1.4 mmol) in 8 ml AcOH. Purification with preparative HPLC ($CH_2Cl_2$/MeOH 2% - 4%) gave **1,10NICz** (57 mg, 0.23 mmol, 38%) and **6,10NICz** (4 mg, 0.02 mmol, 3%) as yellow and light brown solid, respectively. $^1$H NMR according to the preparation starting from **6,10PyCb**.

# 3 NMR Spectra

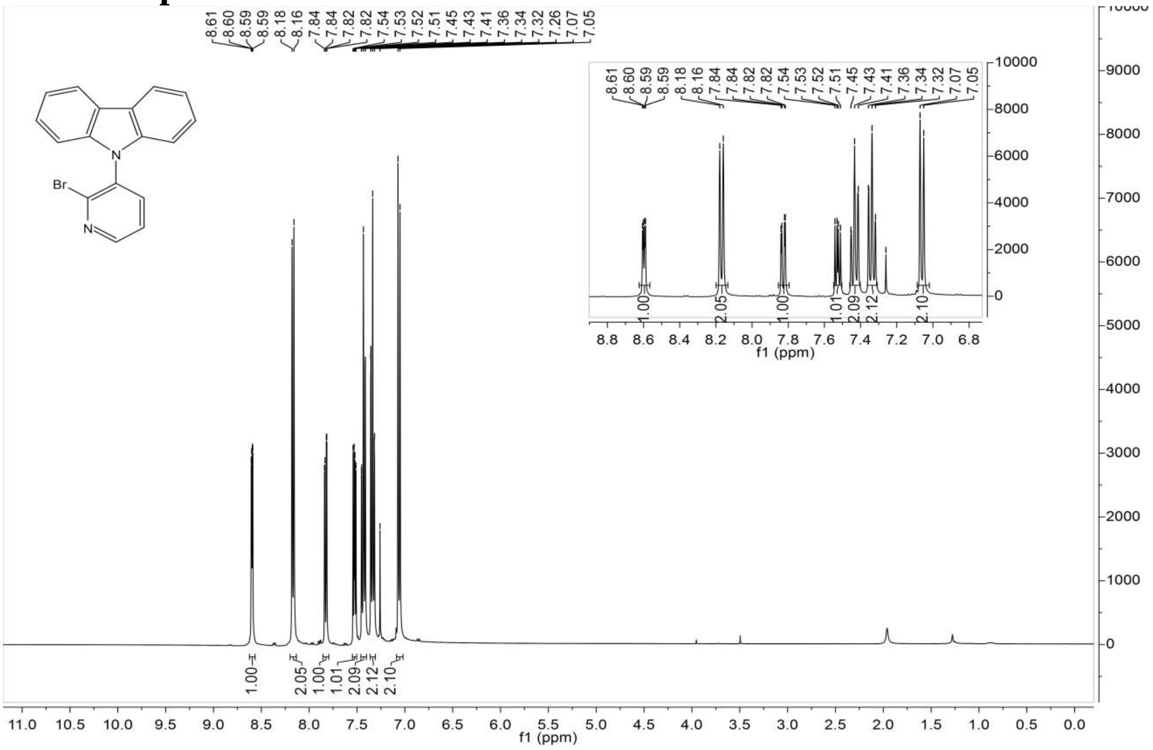

**Figure S1.** $^{1}H$ NMR spectrum of bromo-**4PCz** in $CDCl_3$.

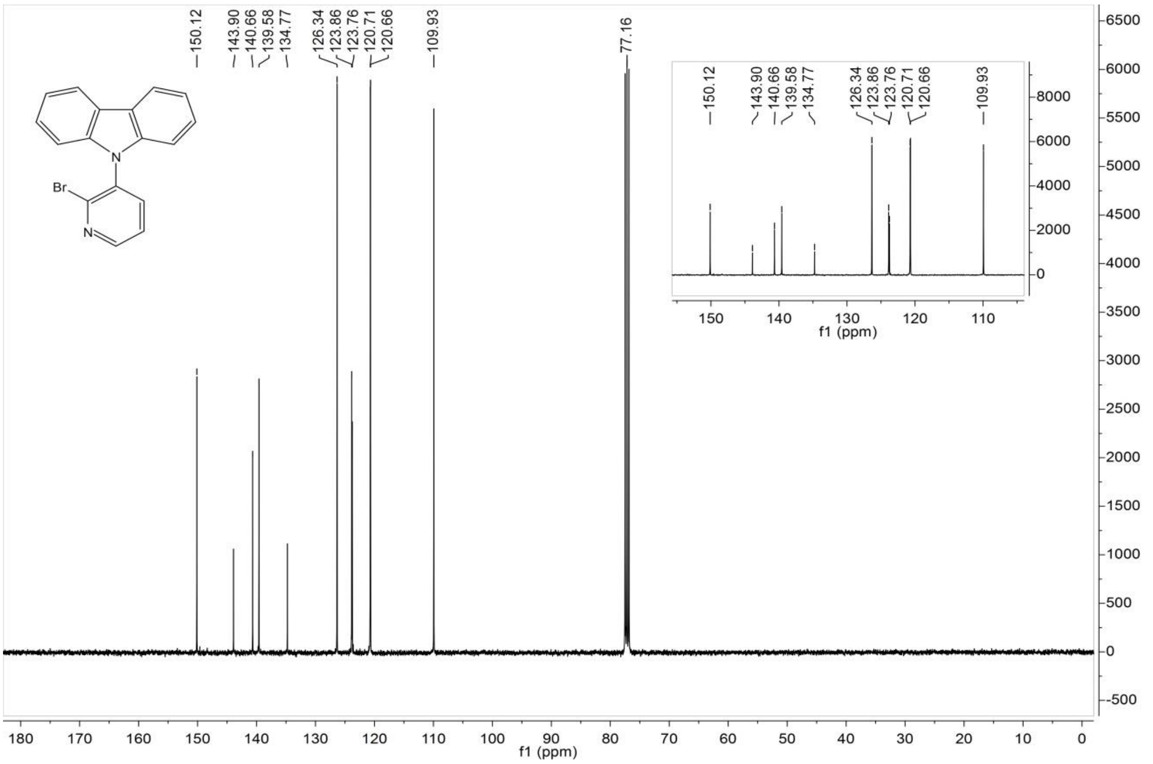

**Figure S2.** $^{13}C$ NMR spectrum of bromo-**4PCz** in $CDCl_3$.

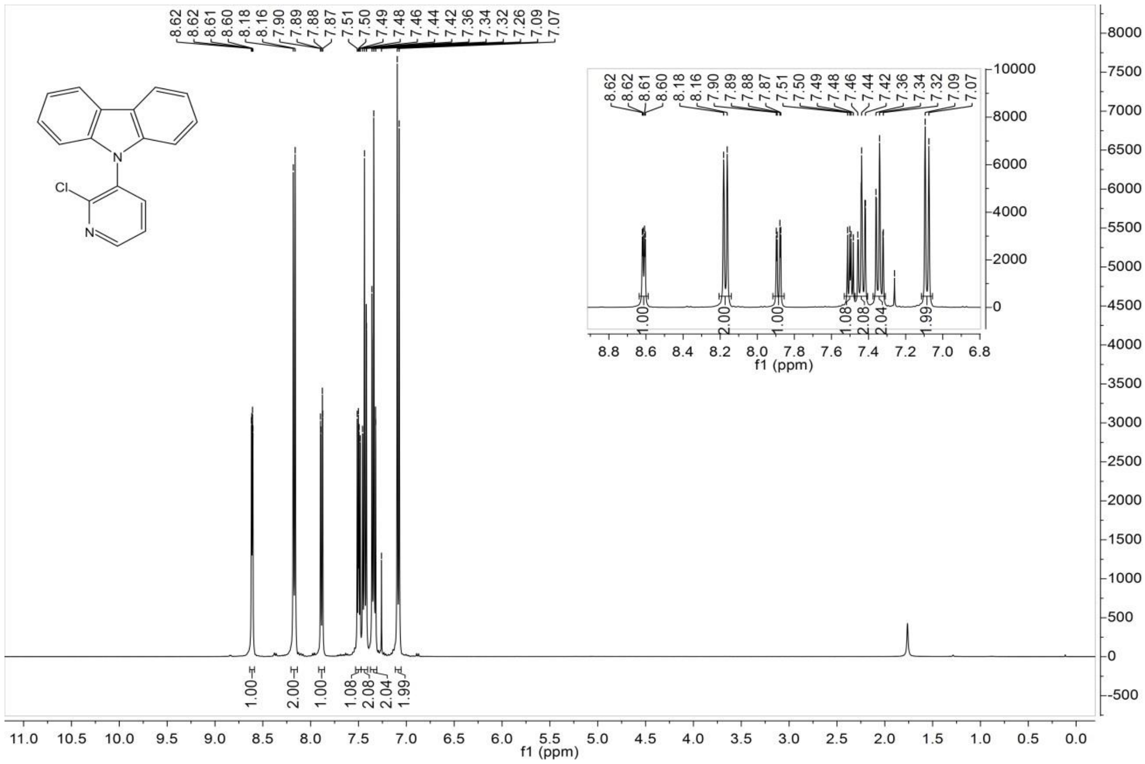

**Figure S3.** $^{1}$H NMR spectrum of chloro-**4PCz** in $CDCl_3$.

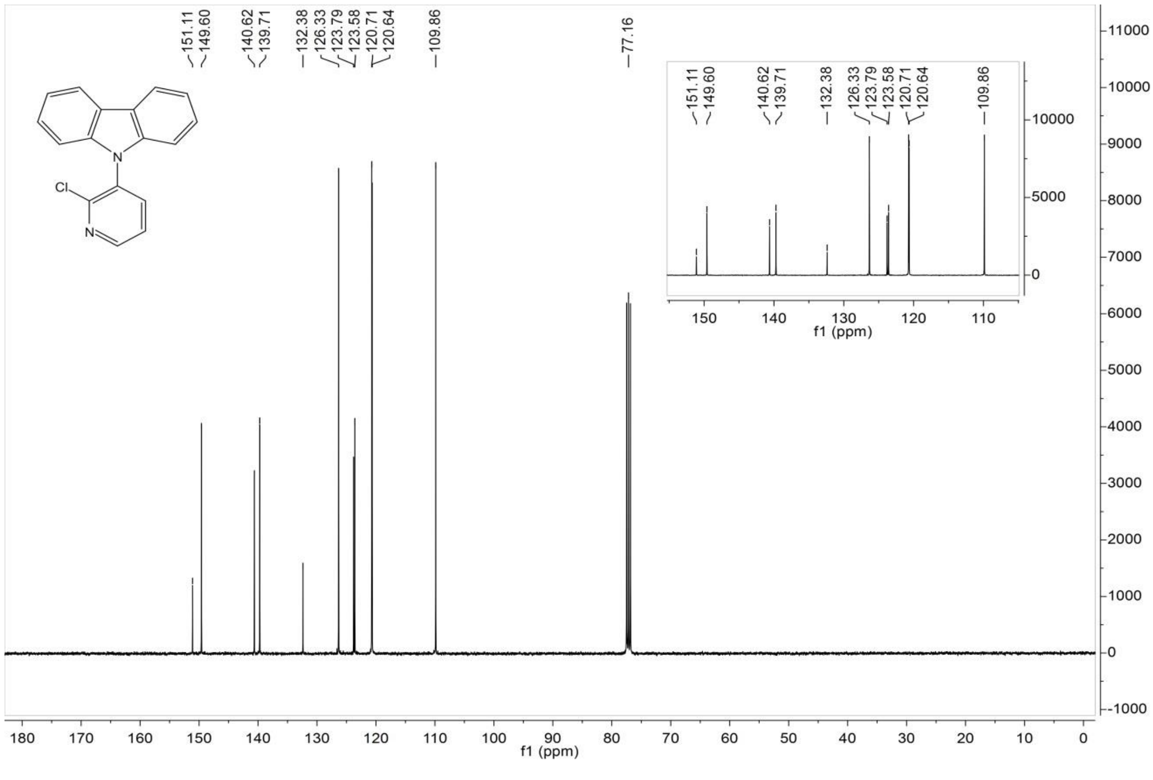

**Figure S4.** $^{13}$C NMR spectrum of chloro-**4PCz** in $CDCl_3$.

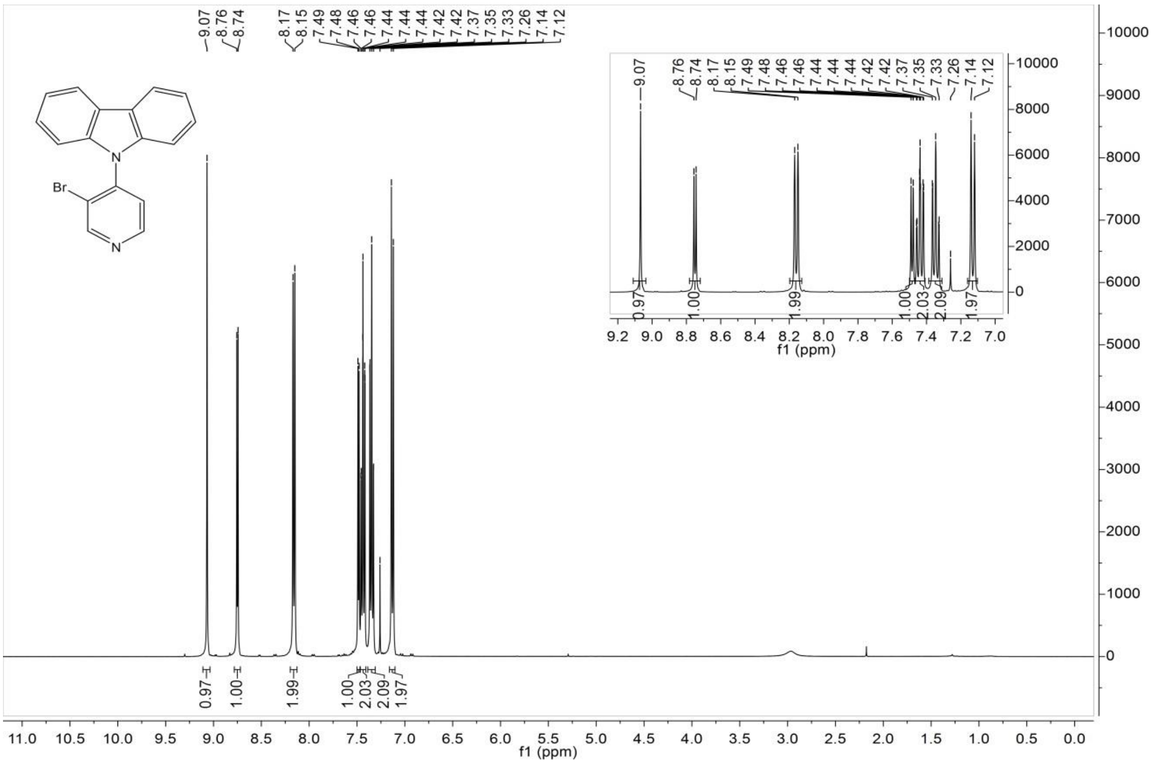

**Figure S5.** $^{1}H$ NMR spectrum of bromo-**5PCz** in $CDCl_3$.

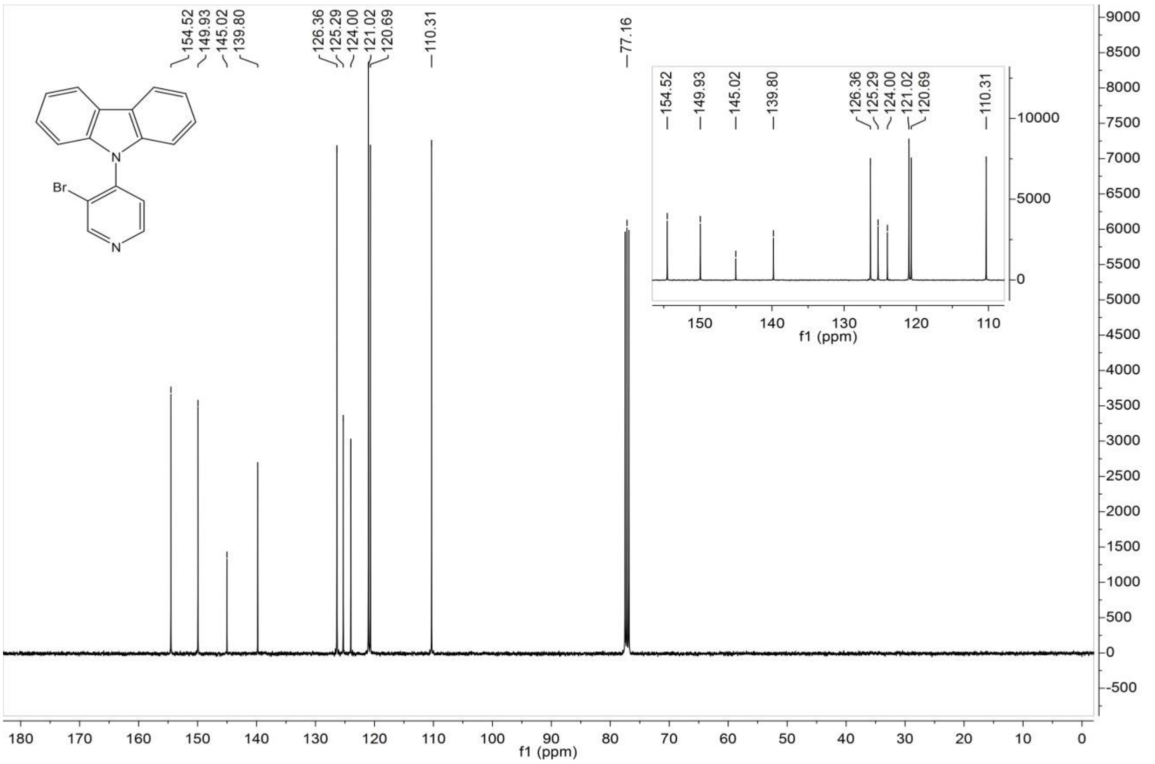

**Figure S6.** $^{13}C$ NMR spectrum of bromo-**5PCz** in $CDCl_3$.

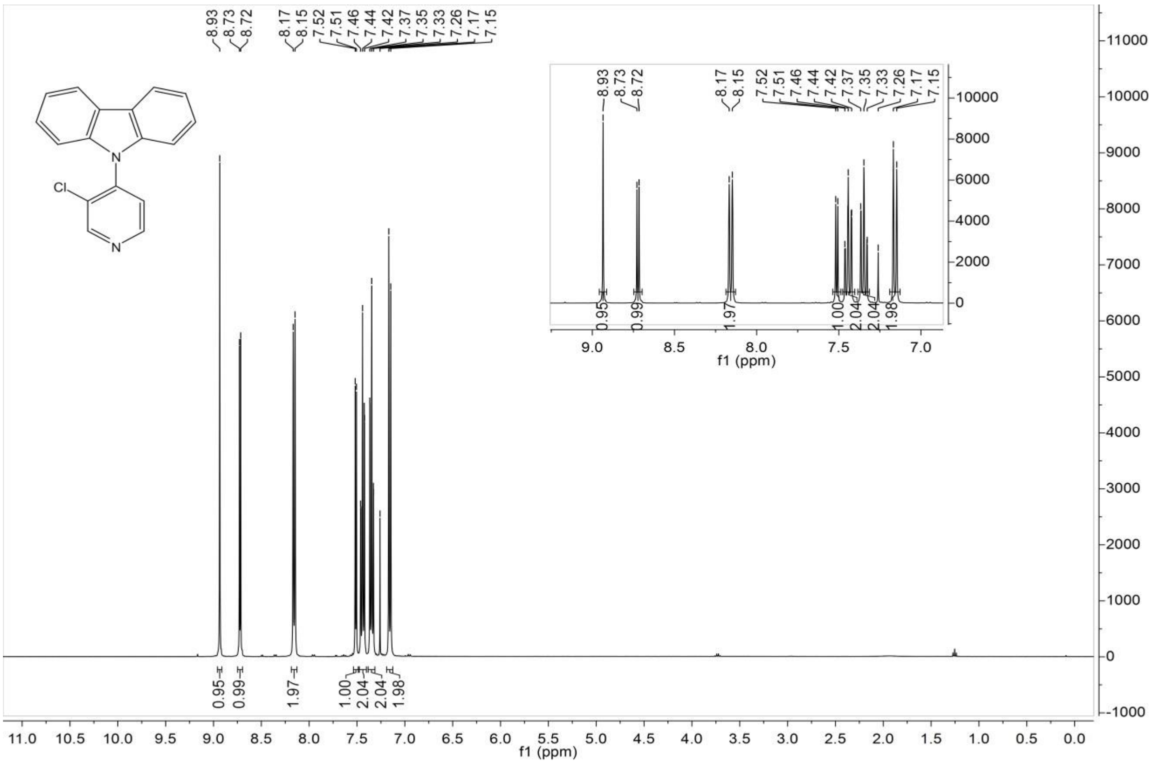


**Figure S7.** $^{1}$H NMR spectrum of chloro-**5PCz** in $CDCl_3$.

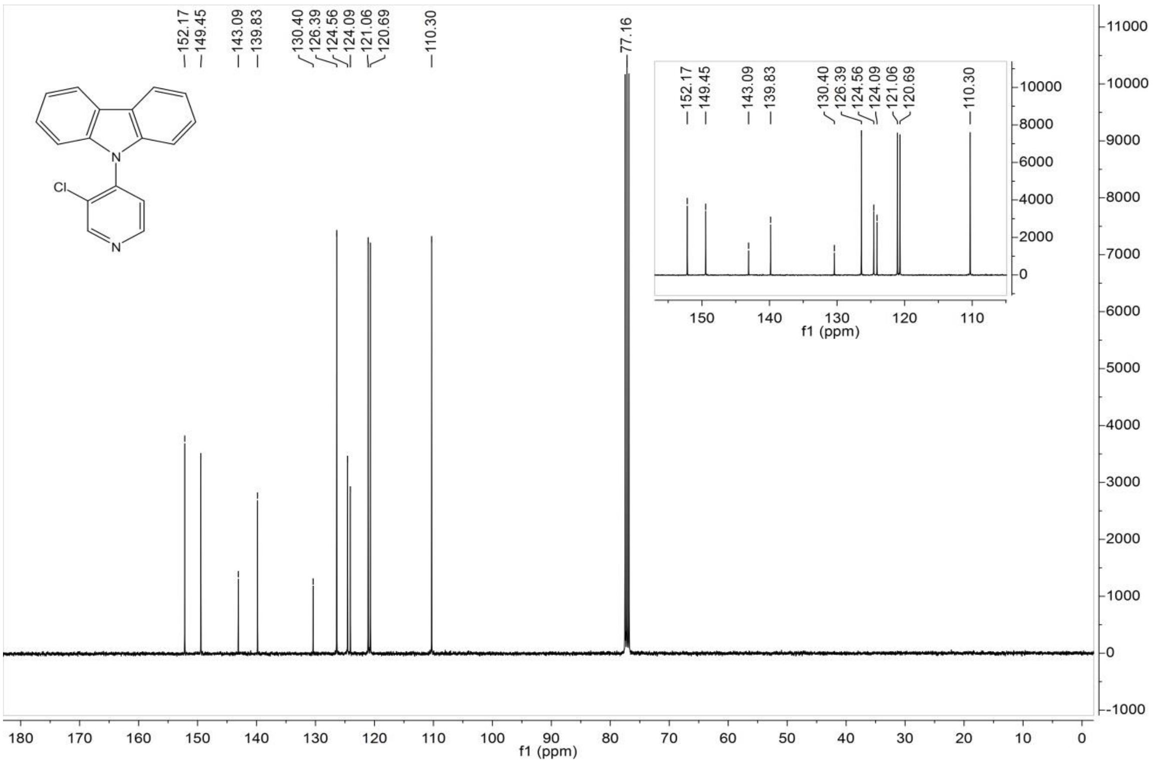


**Figure S8.** $^{13}$C NMR spectrum of chloro-**5PCz** in $CDCl_3$.

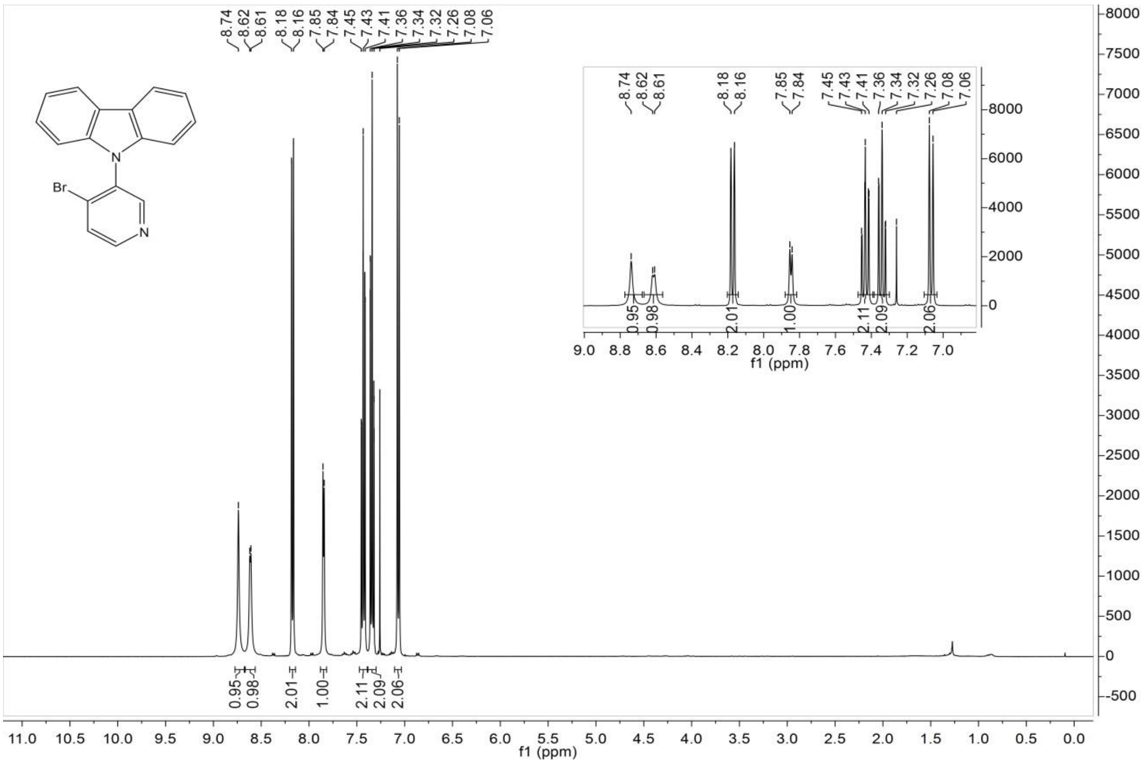

**Figure S9.** $^{1}$H NMR spectrum of bromo-**6PCz** in $CDCl_3$.

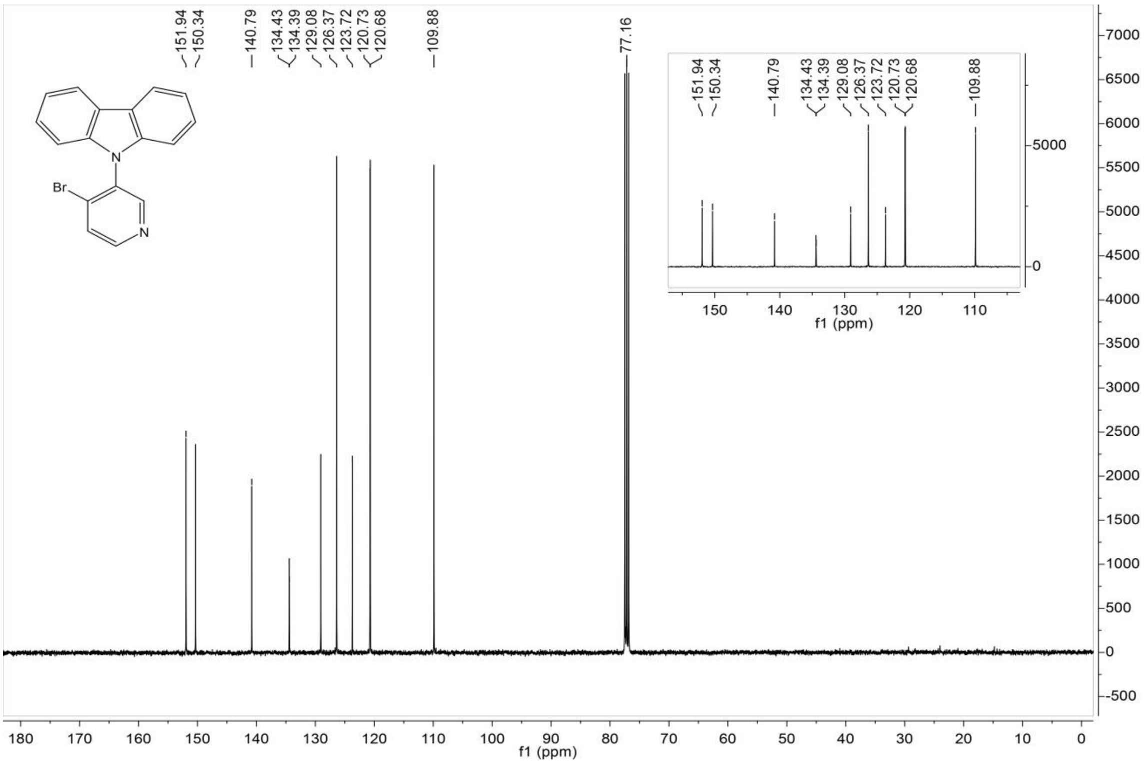

**Figure S10.** $^{13}$C NMR spectrum of bromo-**6PCz** in $CDCl_3$.

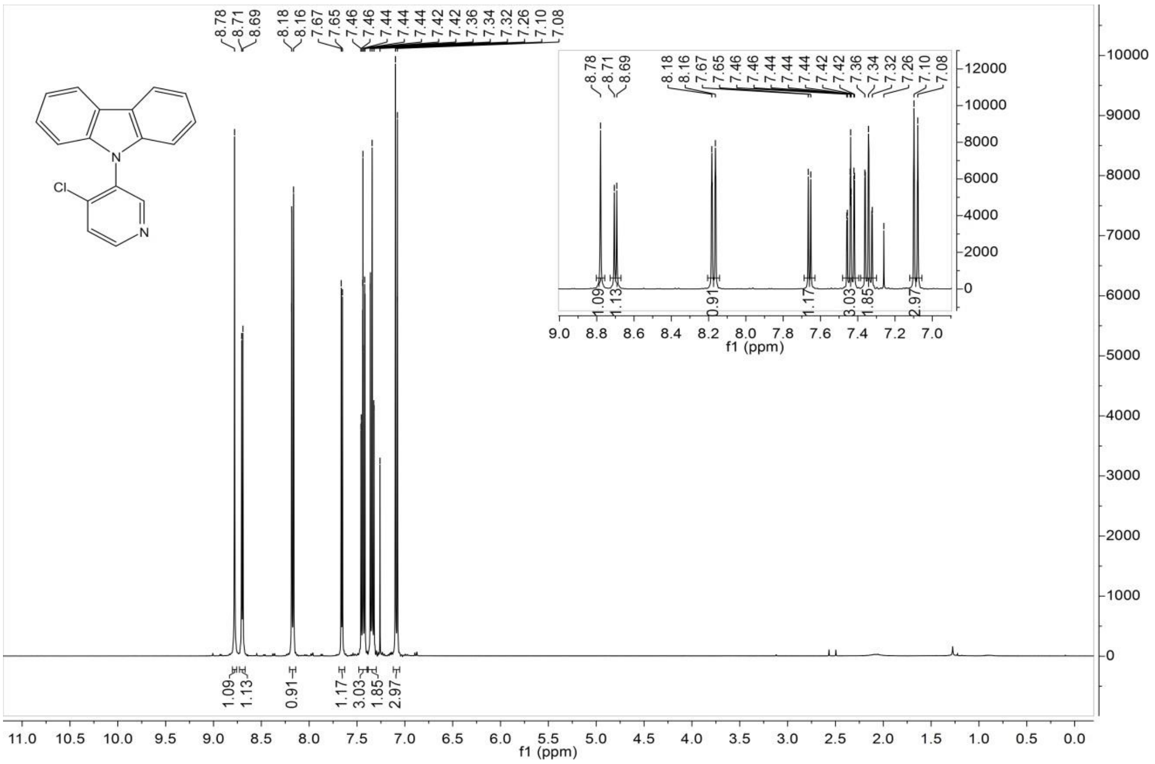

**Figure S11.** $^{1}$H NMR spectrum of chloro-**6PCz** in $CDCl_3$.

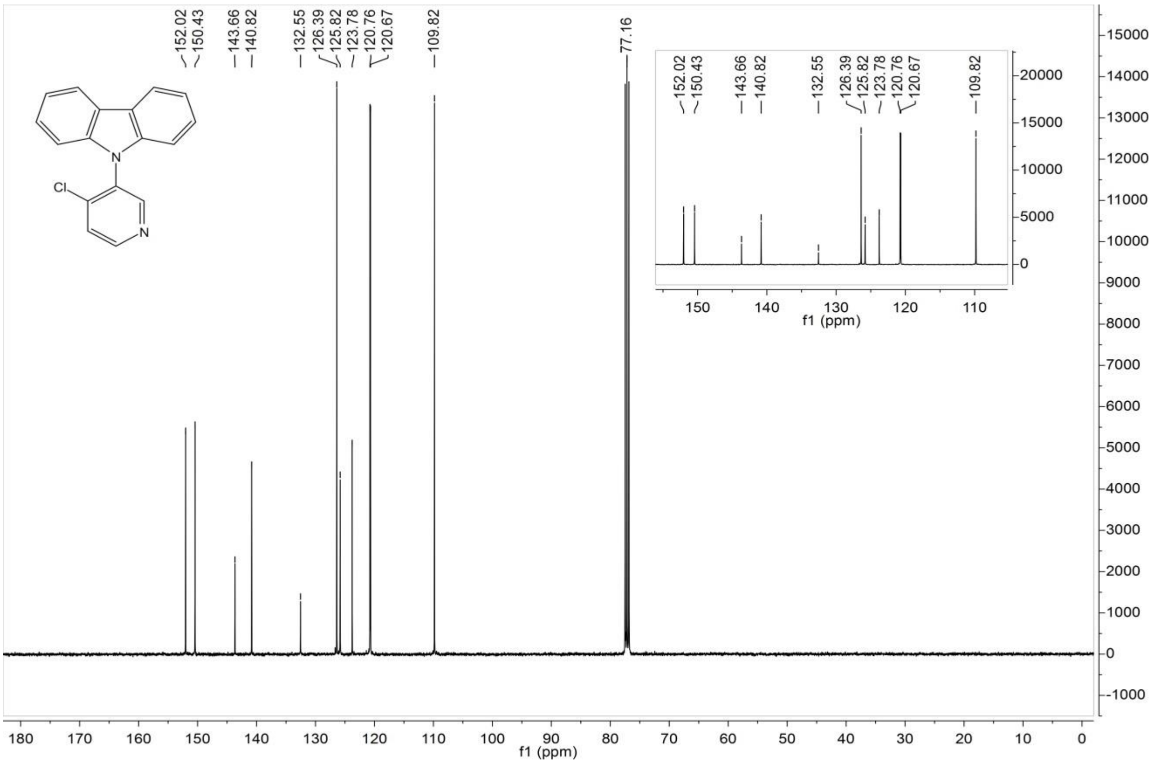

**Figure S12.** $^{13}$C NMR spectrum of chloro-**6PCz** in $CDCl_3$.

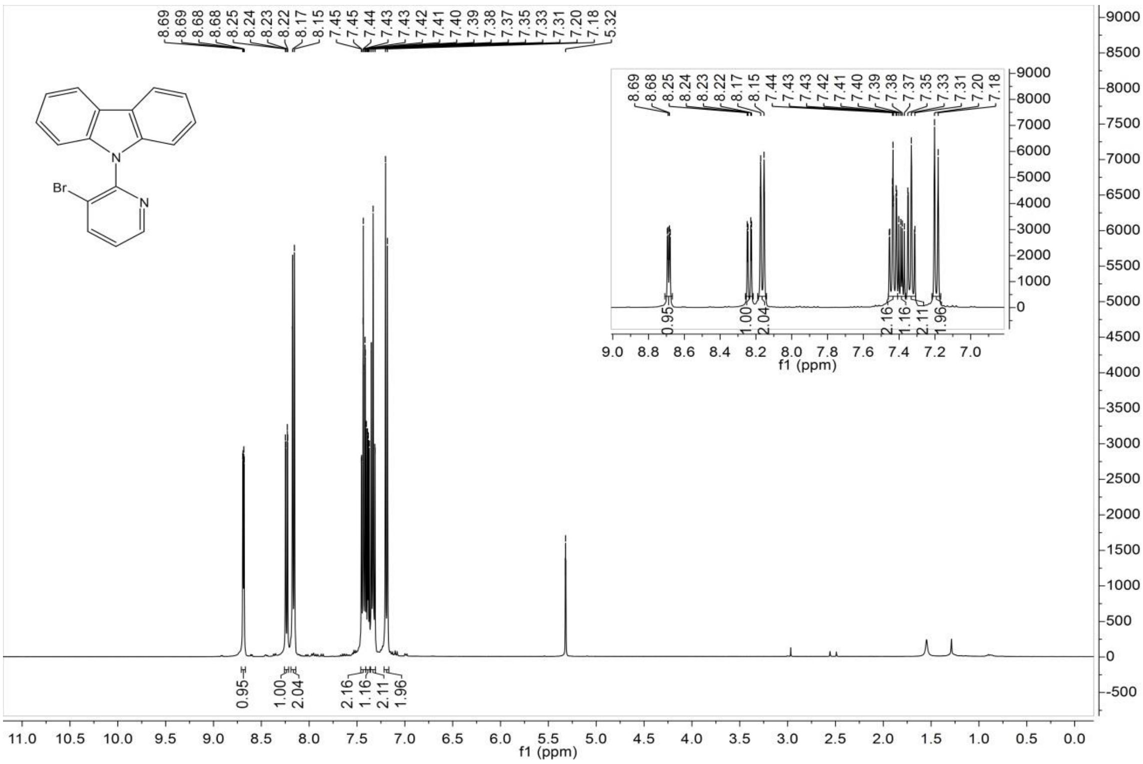


**Figure S13.** $^{1}$H NMR spectrum of bromo-**7PCz** in $CD_2Cl_2$.

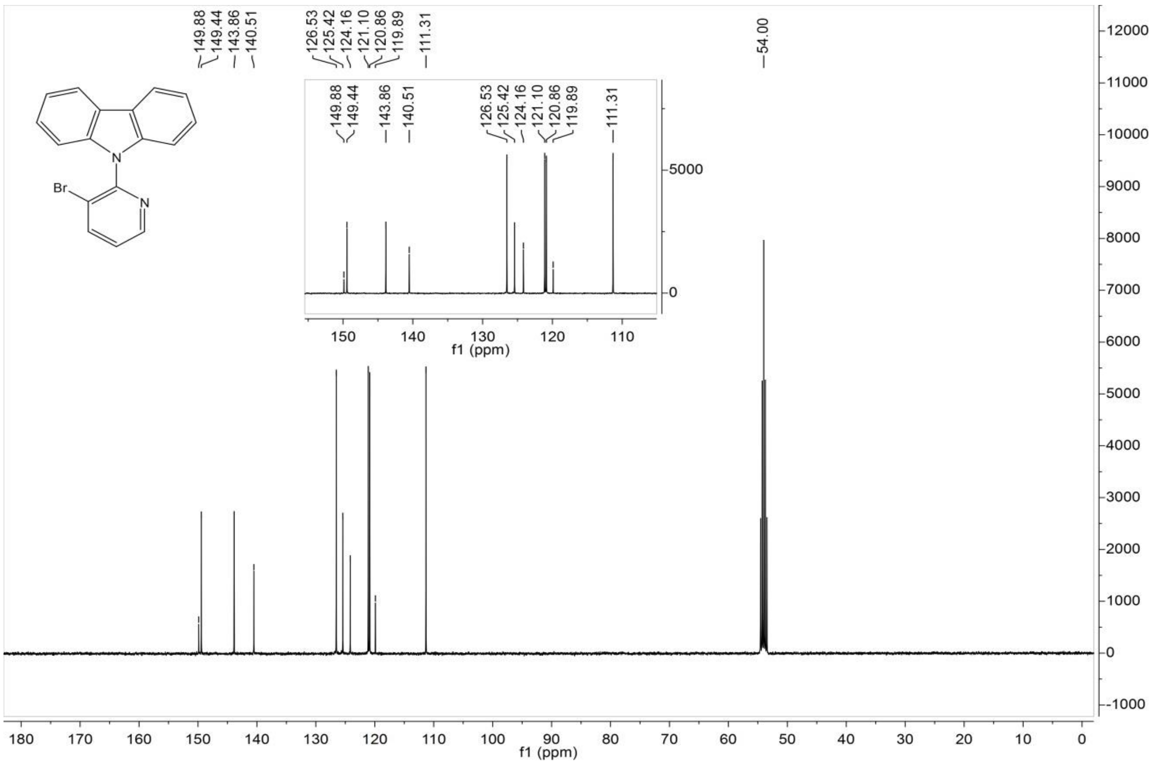


**Figure S14.** $^{13}$C NMR spectrum of bromo-**7PCz** in $CD_2Cl_2$.

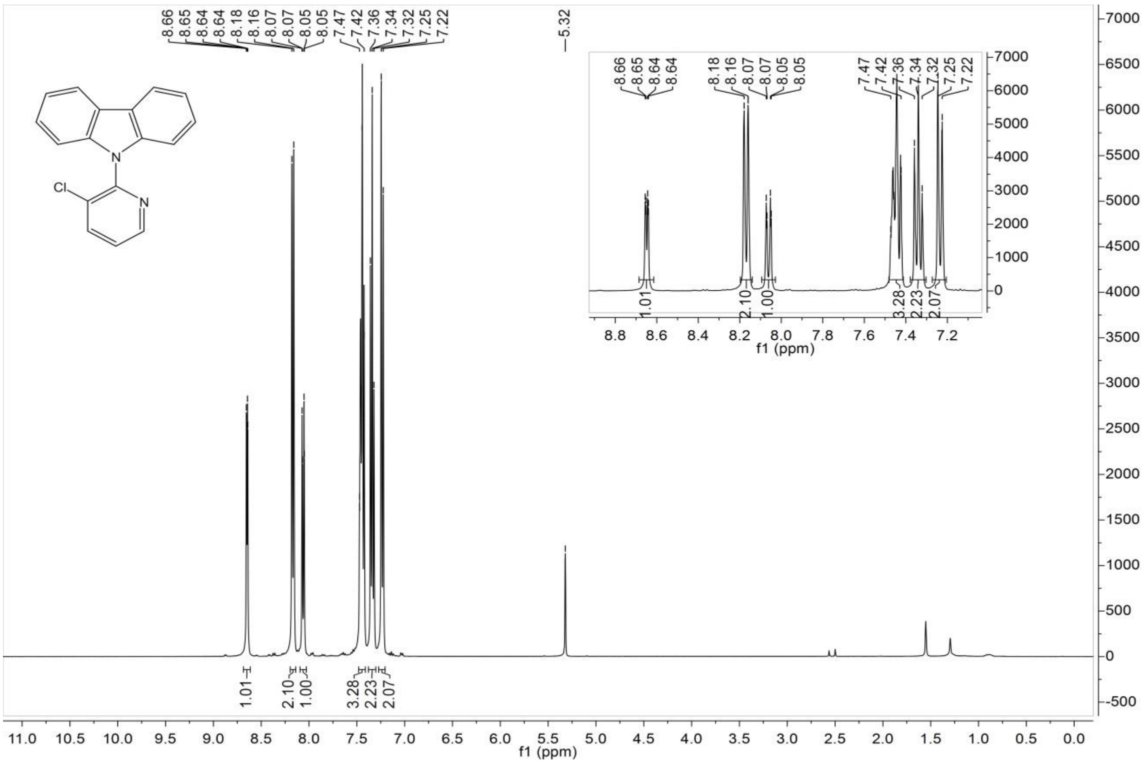


**Figure S15.** $^{1}H$ NMR spectrum of chloro-**7PCz** in $CD_2Cl_2$.

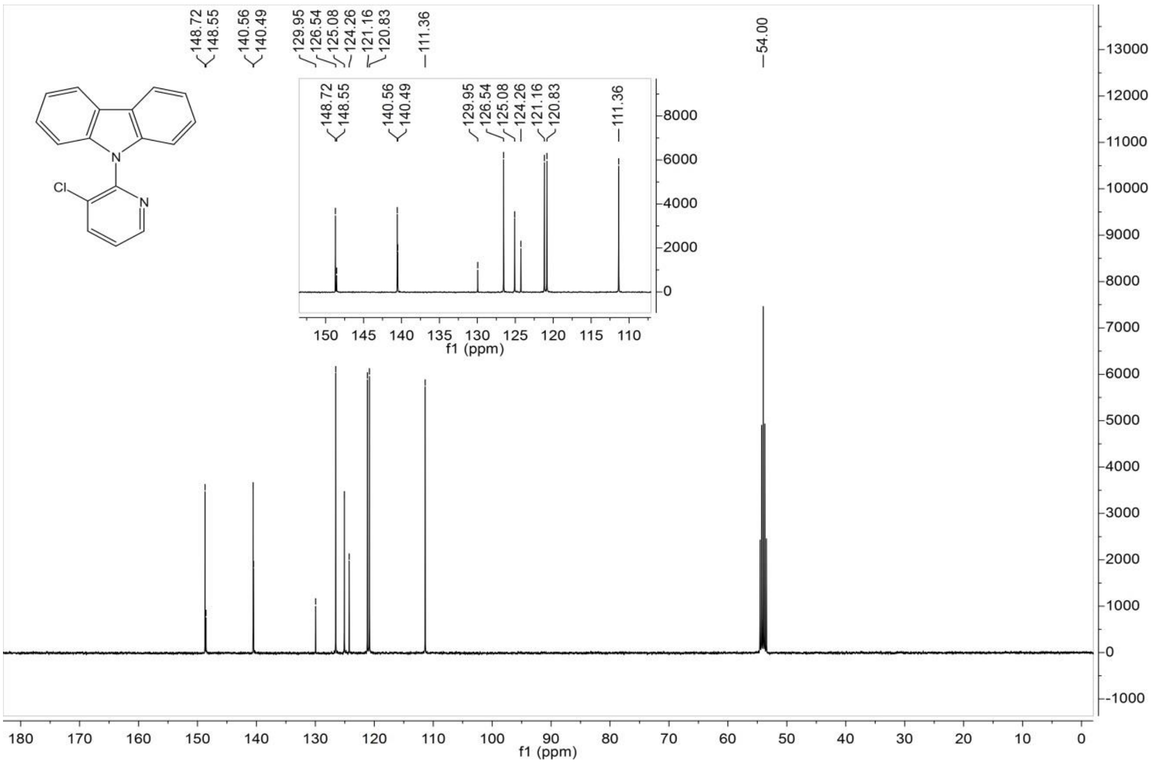


**Figure S16.** $^{13}C$ NMR spectrum of chloro-**7PCz** in $CD_2Cl_2$.

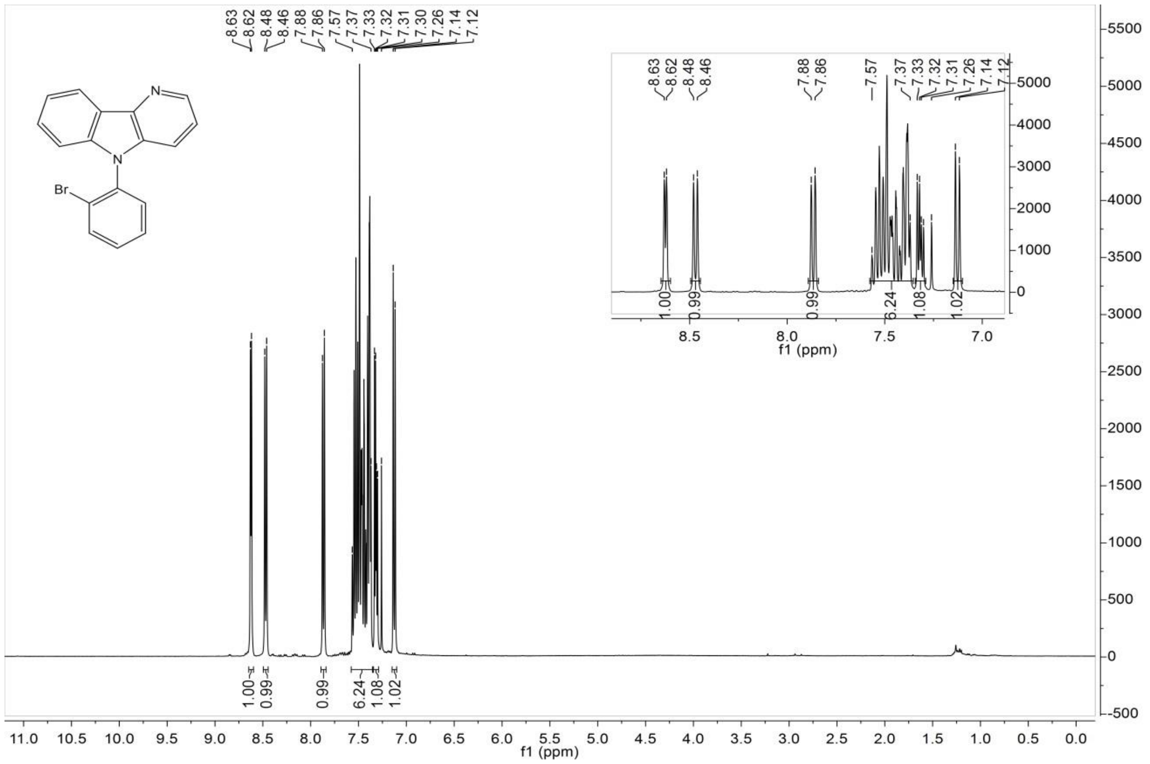

**Figure S17.** $^{1}$H NMR spectrum of **4PCb** in $CDCl_3$.

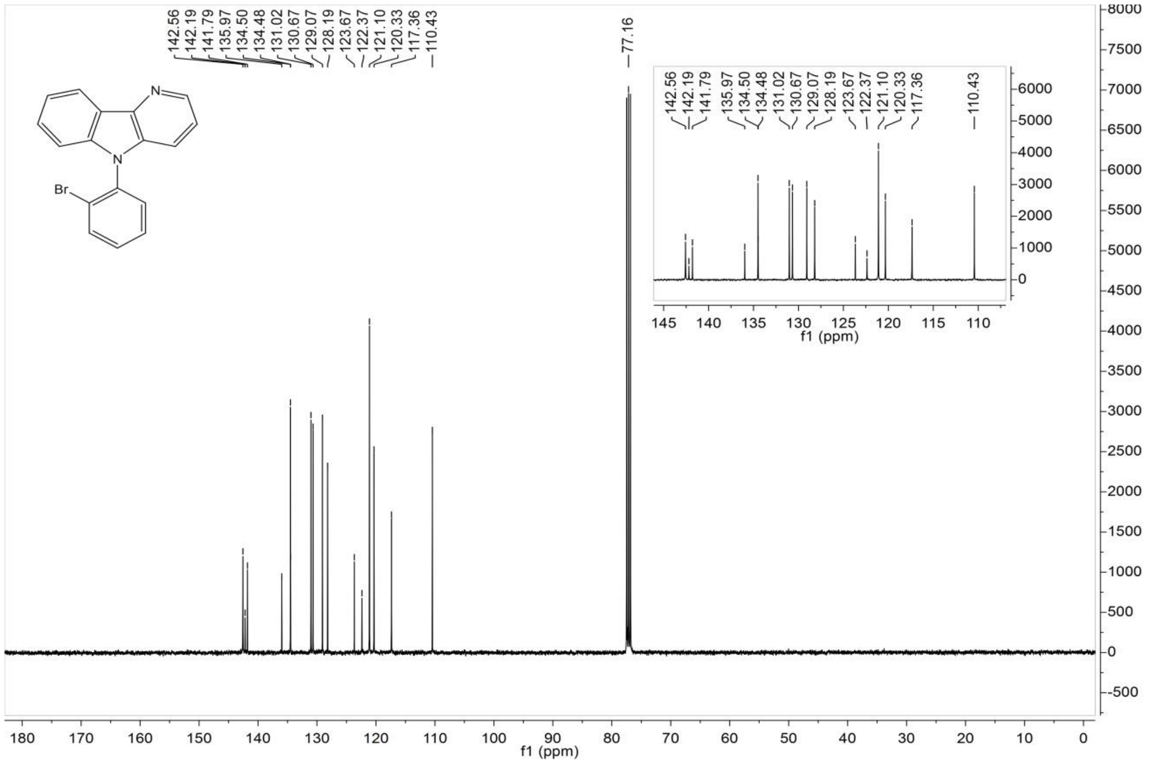

**Figure S18.** $^{13}$C NMR spectrum of **4PCb** in $CDCl_3$.

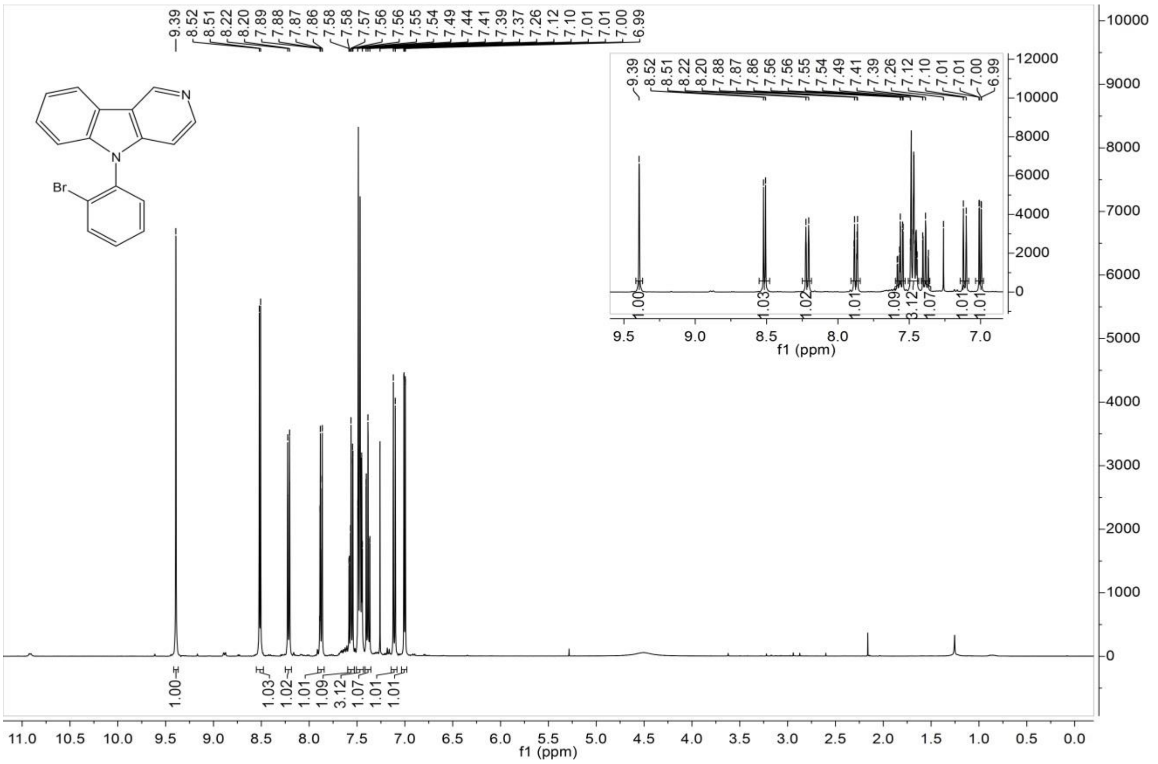

**Figure S19.** $^{1}$H NMR spectrum of **5PCb** in $CDCl_3$.

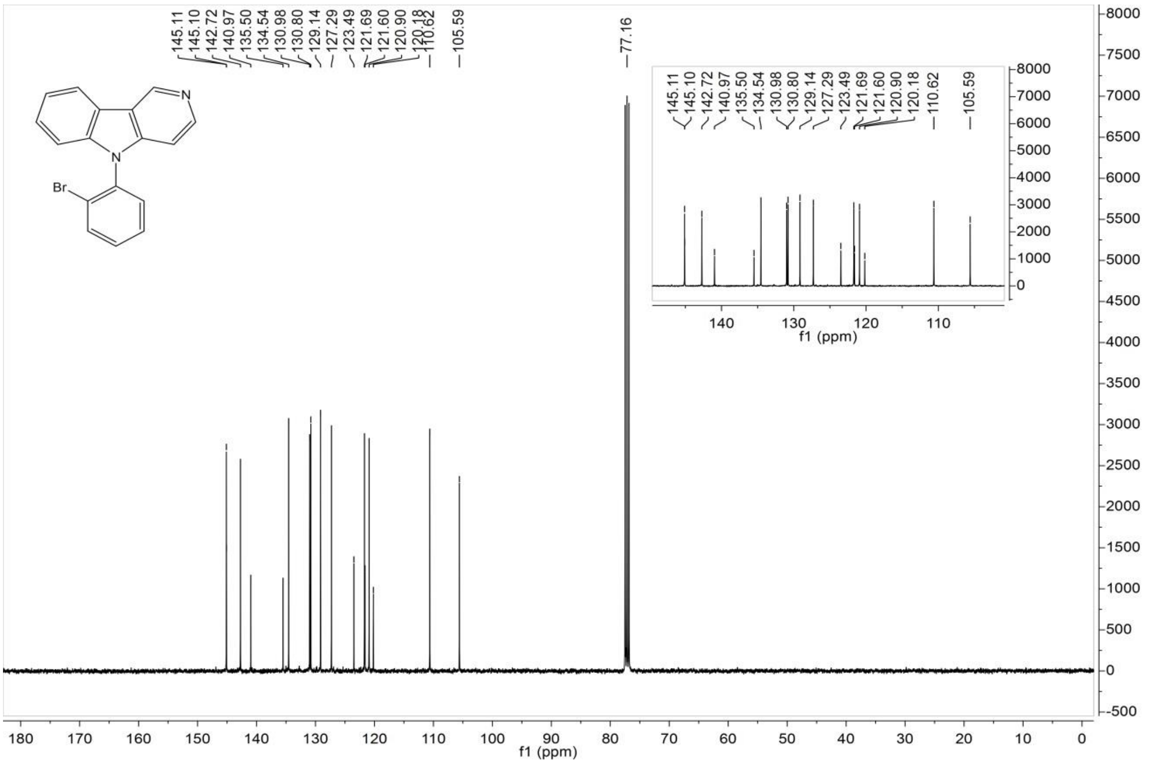

**Figure S20.** $^{13}$C NMR spectrum of **5PCb** in $CDCl_3$.

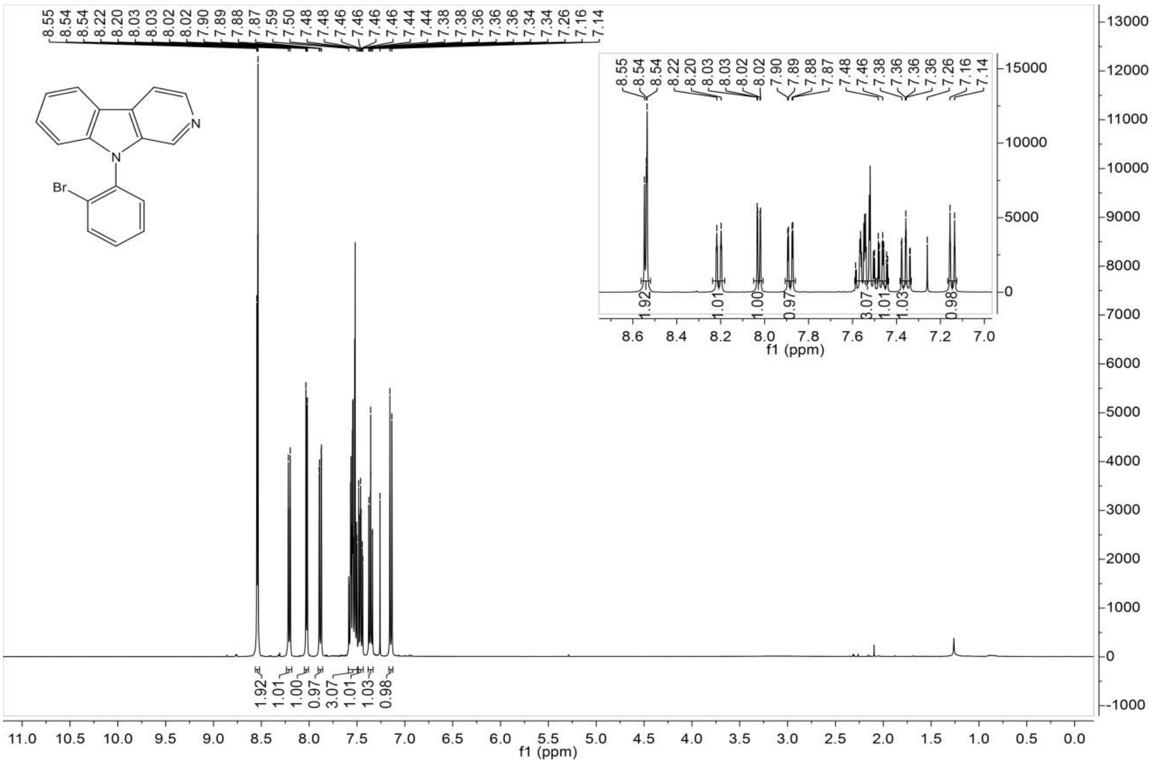

**Figure S21.** $^{1}$H NMR spectrum of **6PCb** in $CDCl_3$.

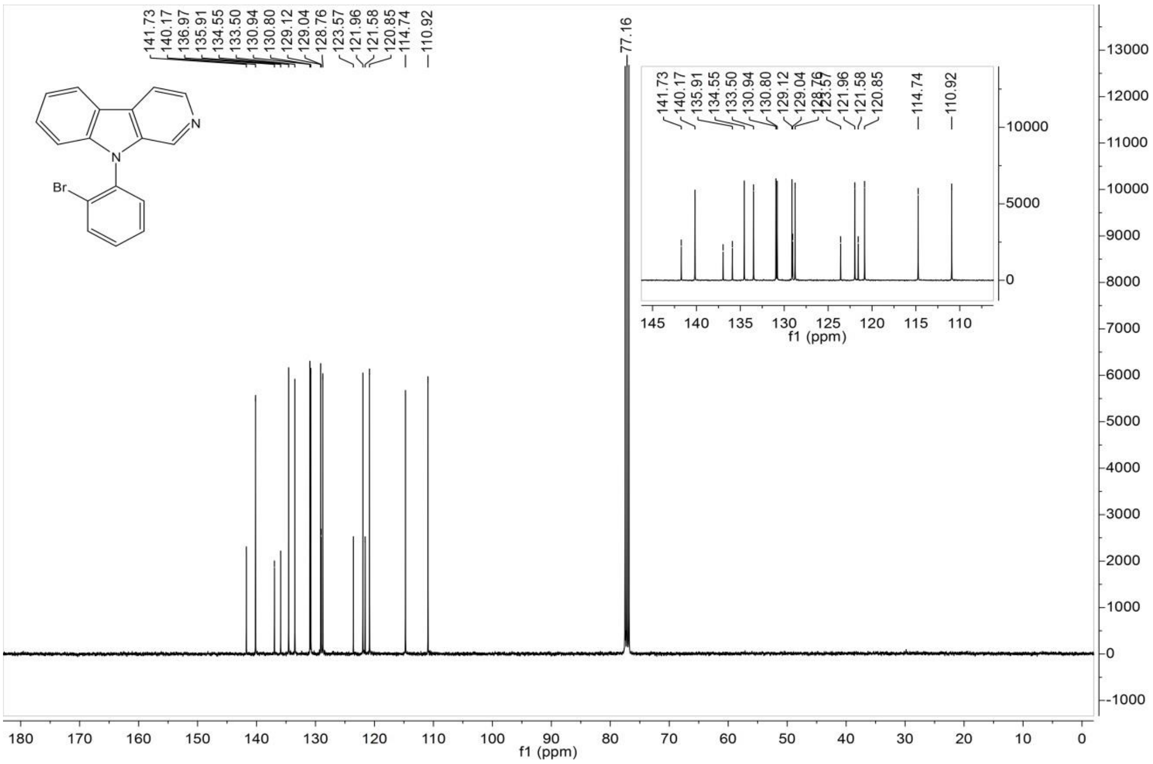

**Figure S22.** $^{13}$C NMR spectrum of **6PCb** in $CDCl_3$.

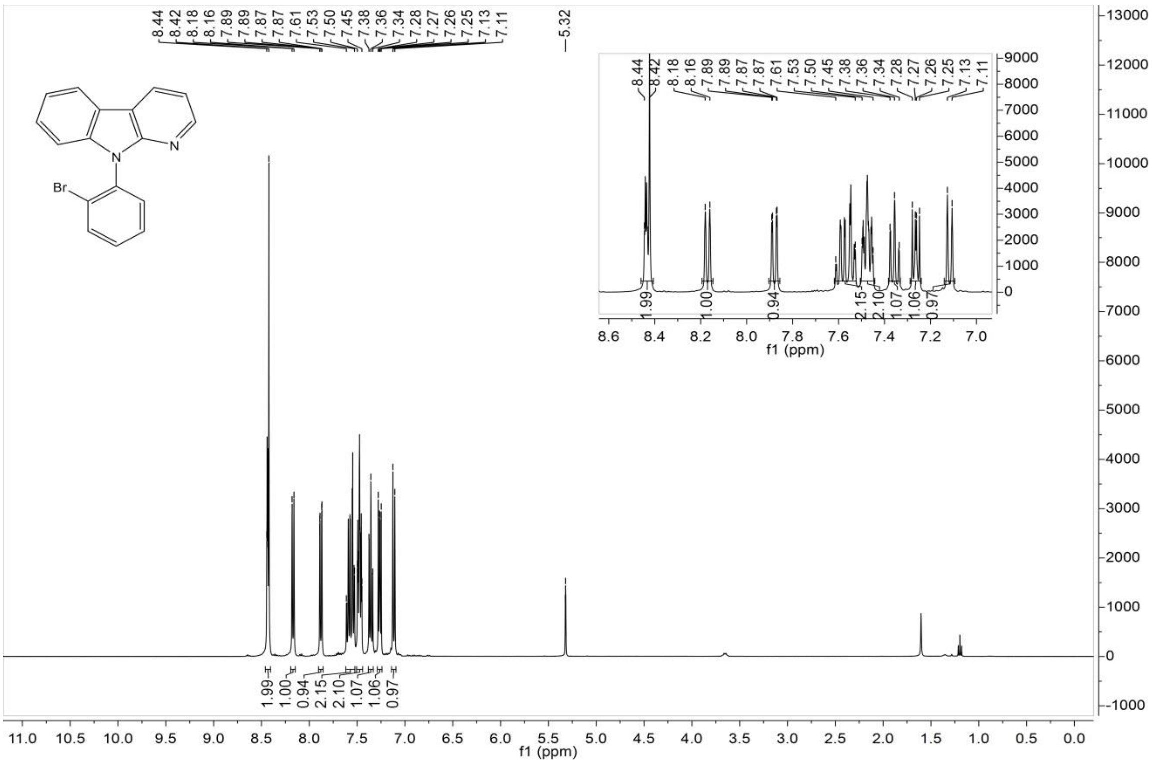

**Figure S23.** $^{1}$H NMR spectrum of **7PCb** in $CD_2Cl_2$.

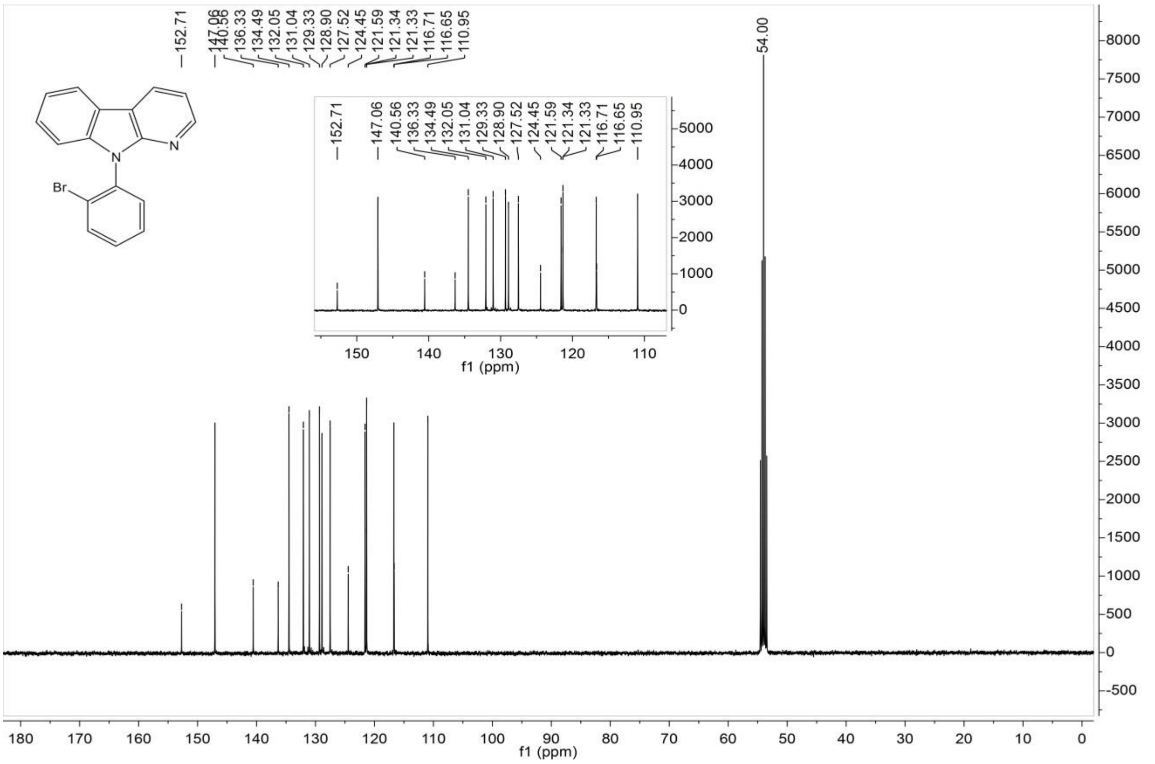

**Figure S24.** $^{13}$C NMR spectrum of **7PCb** in $CD_2Cl_2$.

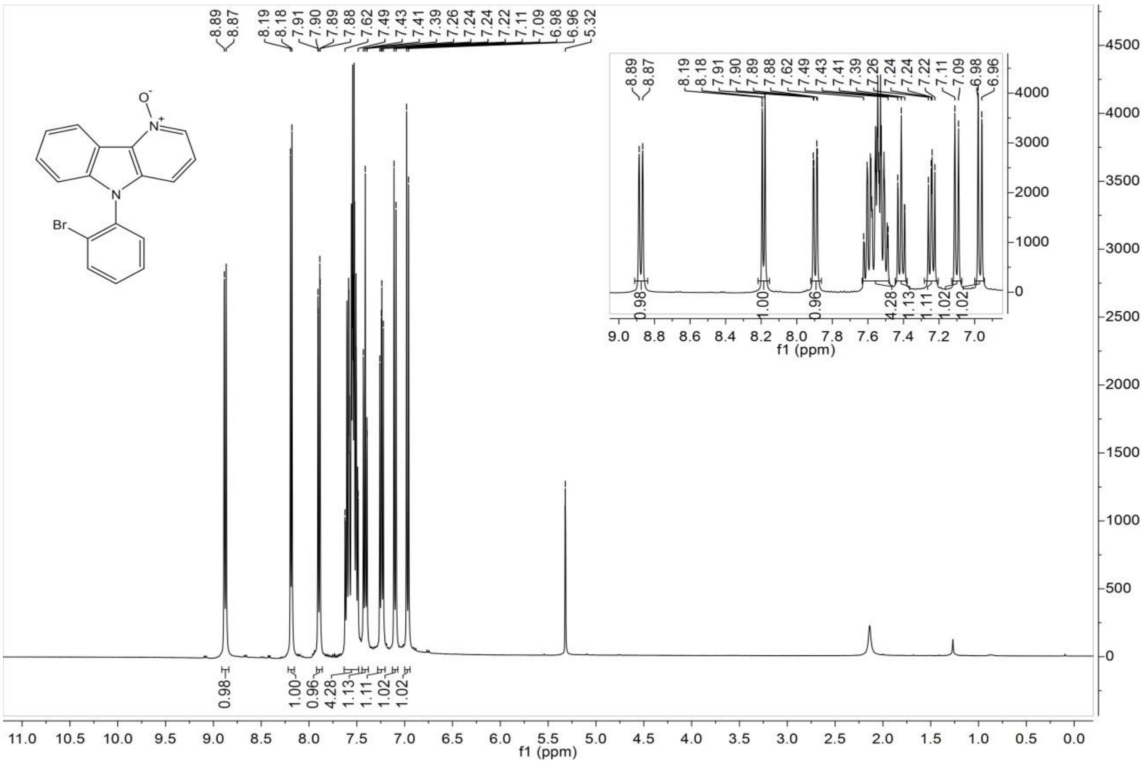


**Figure S25.** $^{1}$H NMR spectrum of **4PCb-Ox** in $CD_2Cl_2$.

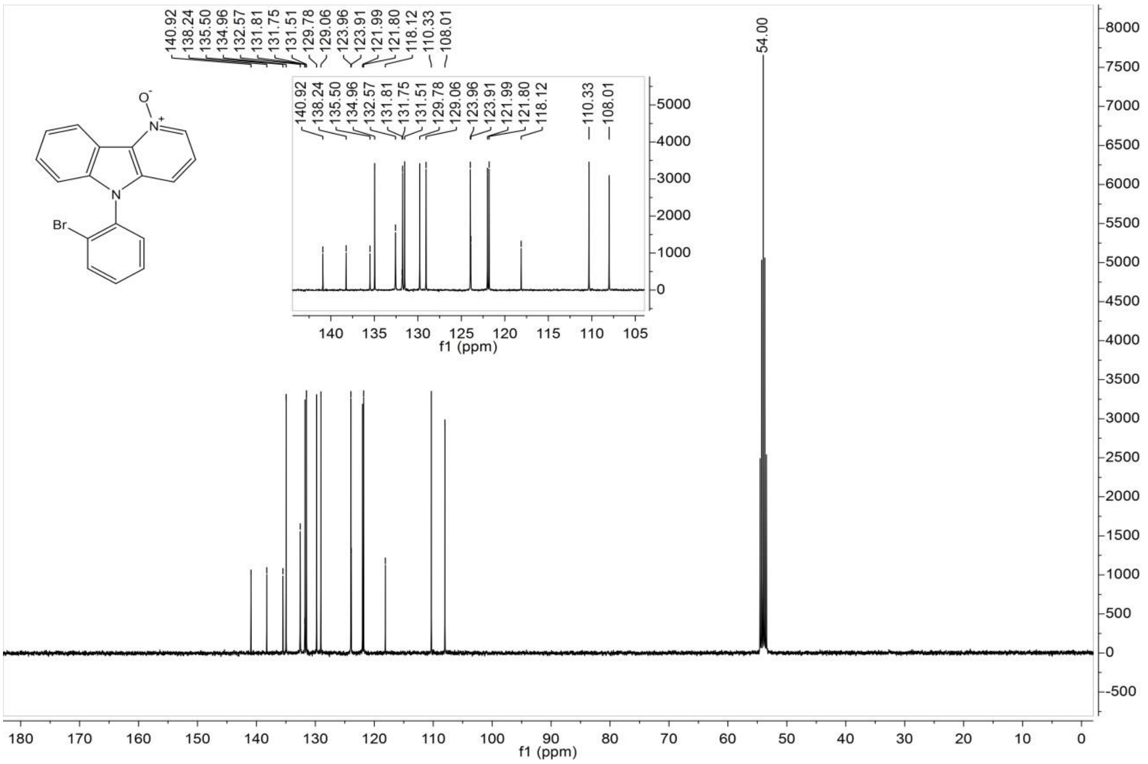


**Figure S26.** $^{13}$C NMR spectrum of **4PCb-Ox** in $CD_2Cl_2$.

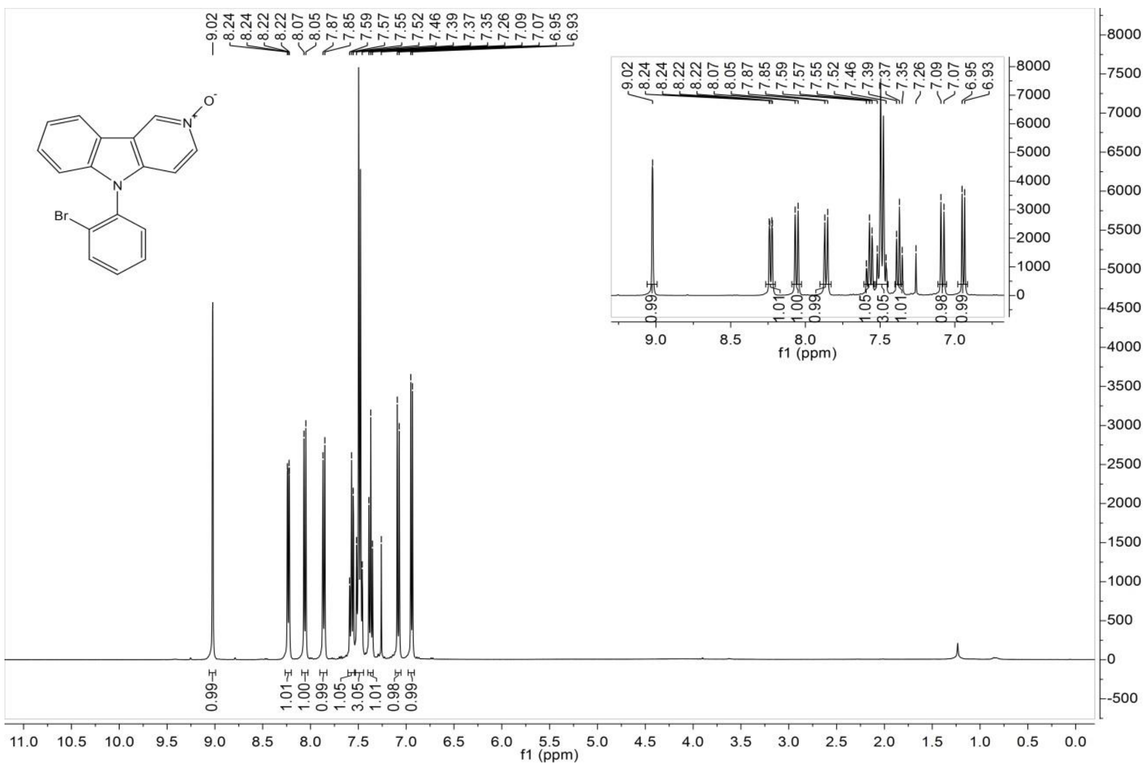

**Figure S27.** $^{1}H$ NMR spectrum of **5PCb-Ox** in $CDCl_3$.

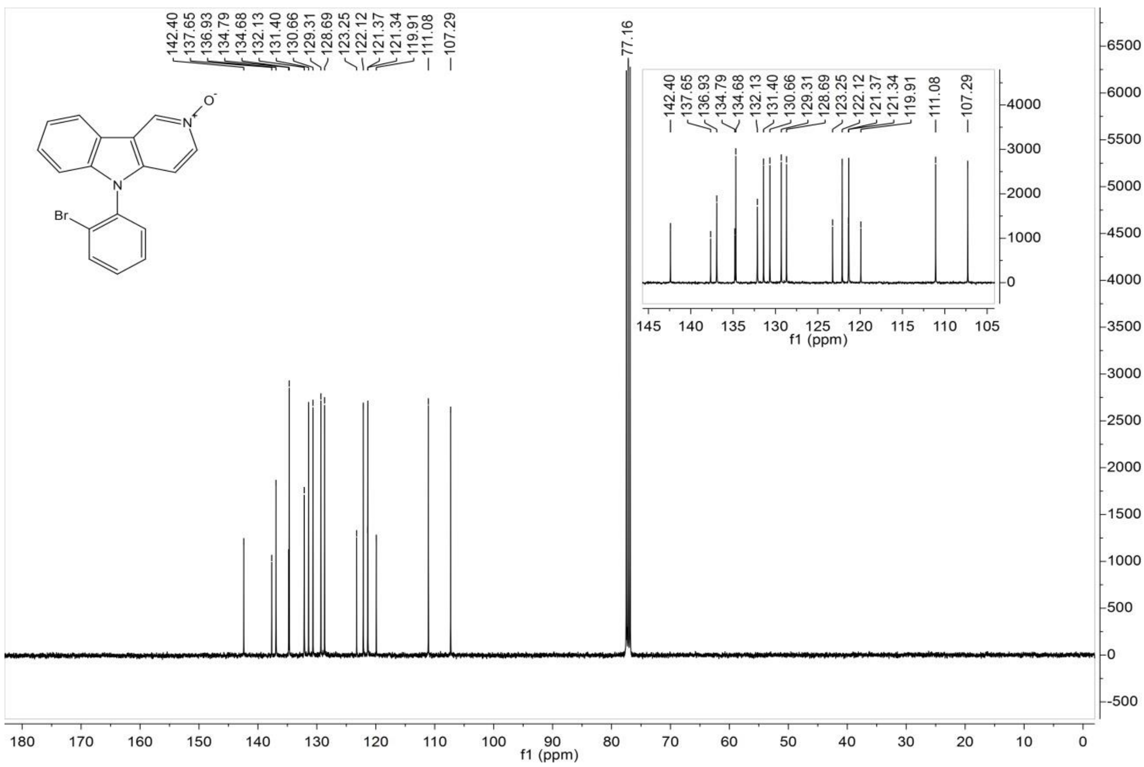

**Figure S28.** $^{13}C$ NMR spectrum of **5PCb-Ox** in $CDCl_3$.

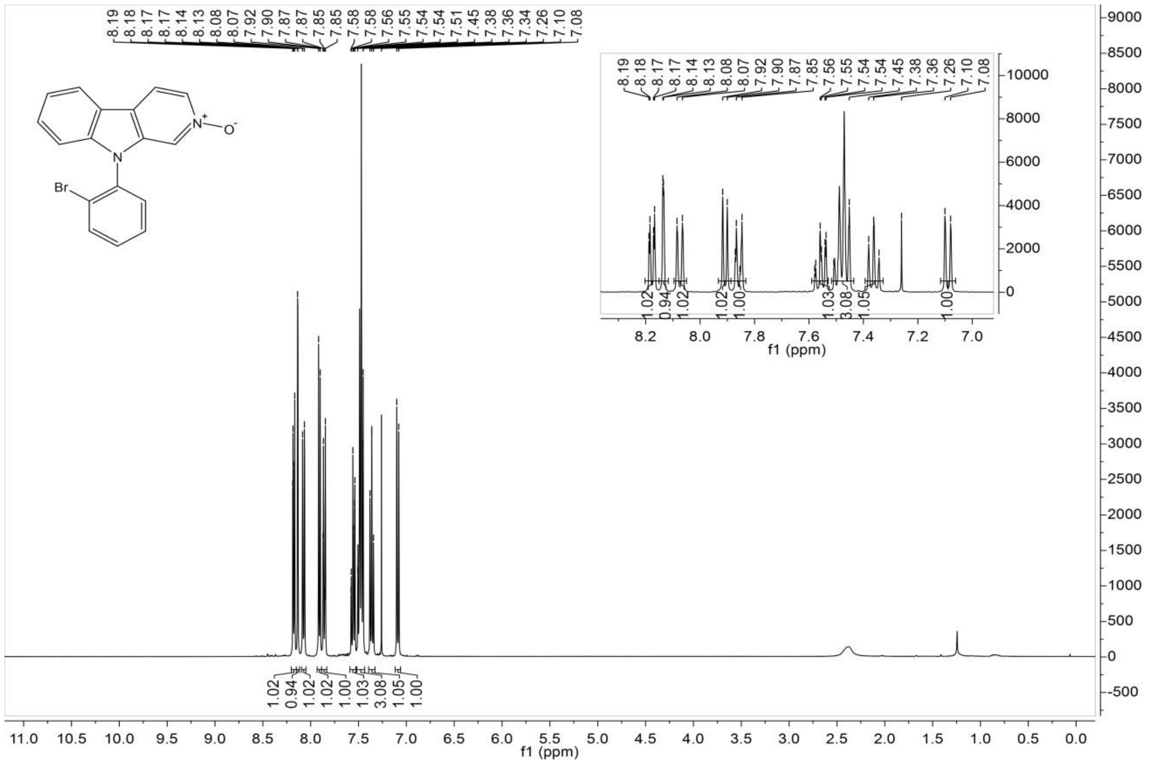

**Figure S29.** $^{1}$H NMR spectrum of **6PCb-Ox** in $CDCl_3$.

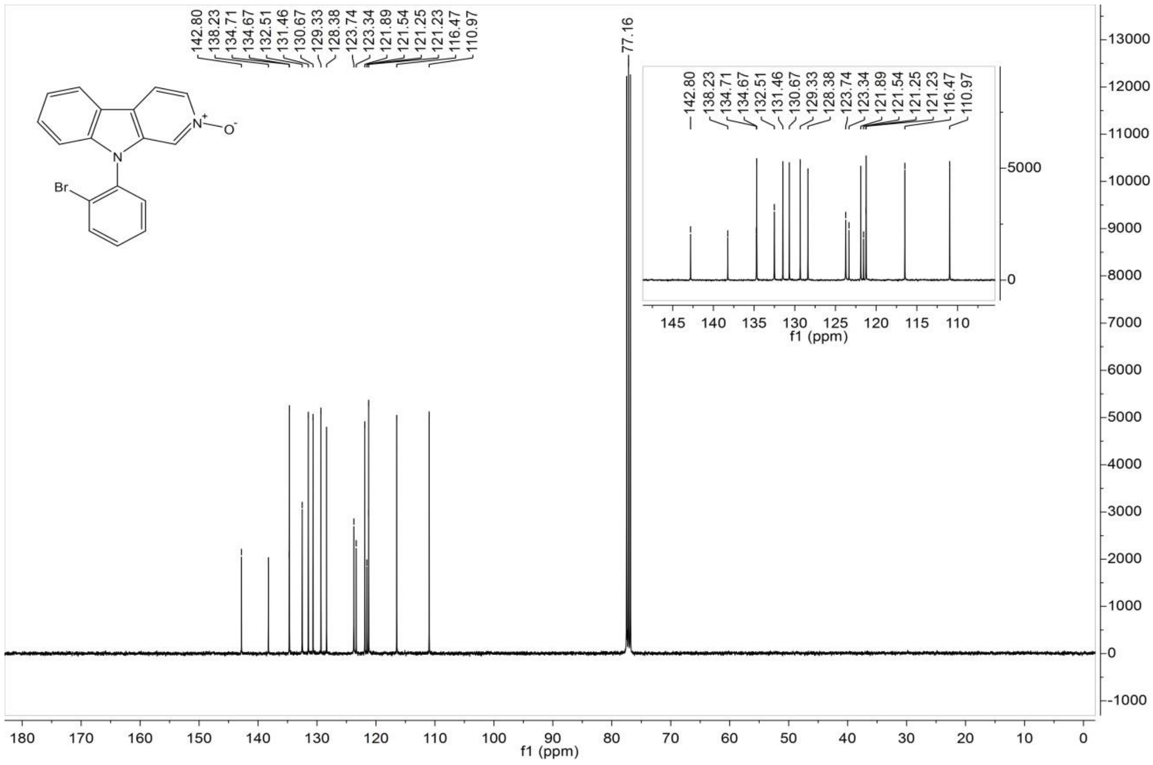

**Figure S30.** $^{13}$C NMR spectrum of **6PCb-Ox** in $CDCl_3$.

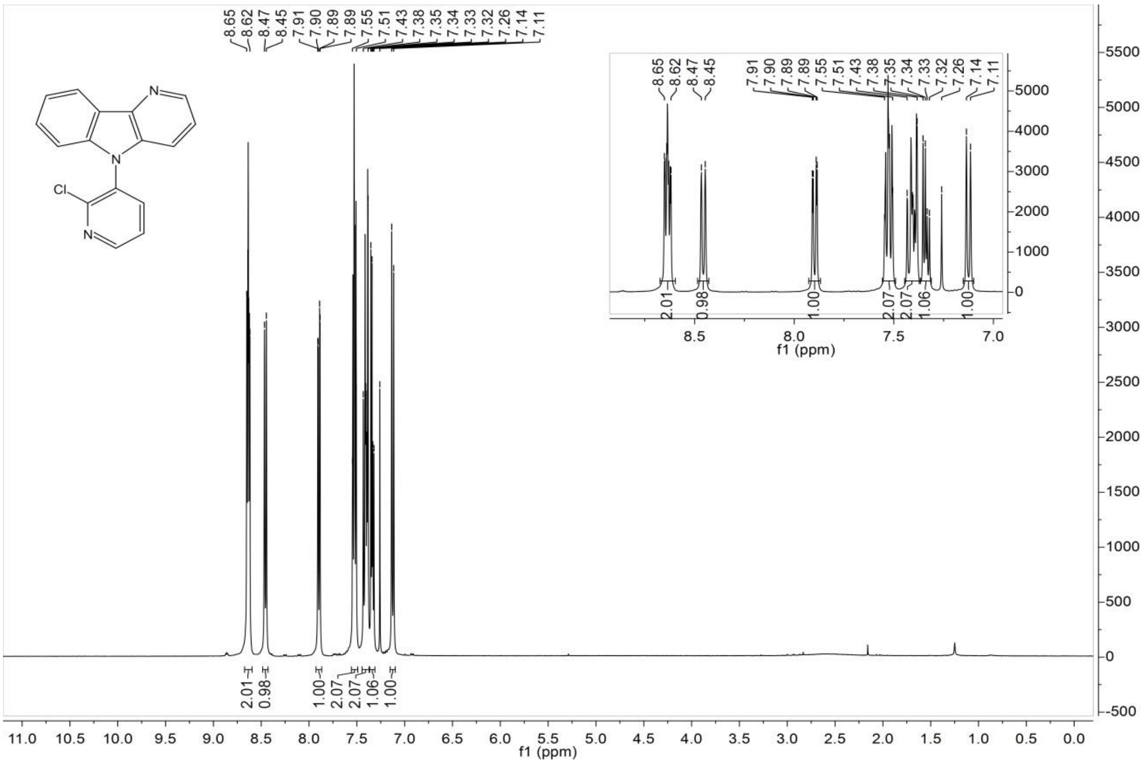

**Figure S31.** $^{1}$H NMR spectrum of **4,12PyCb** in $CDCl_3$.

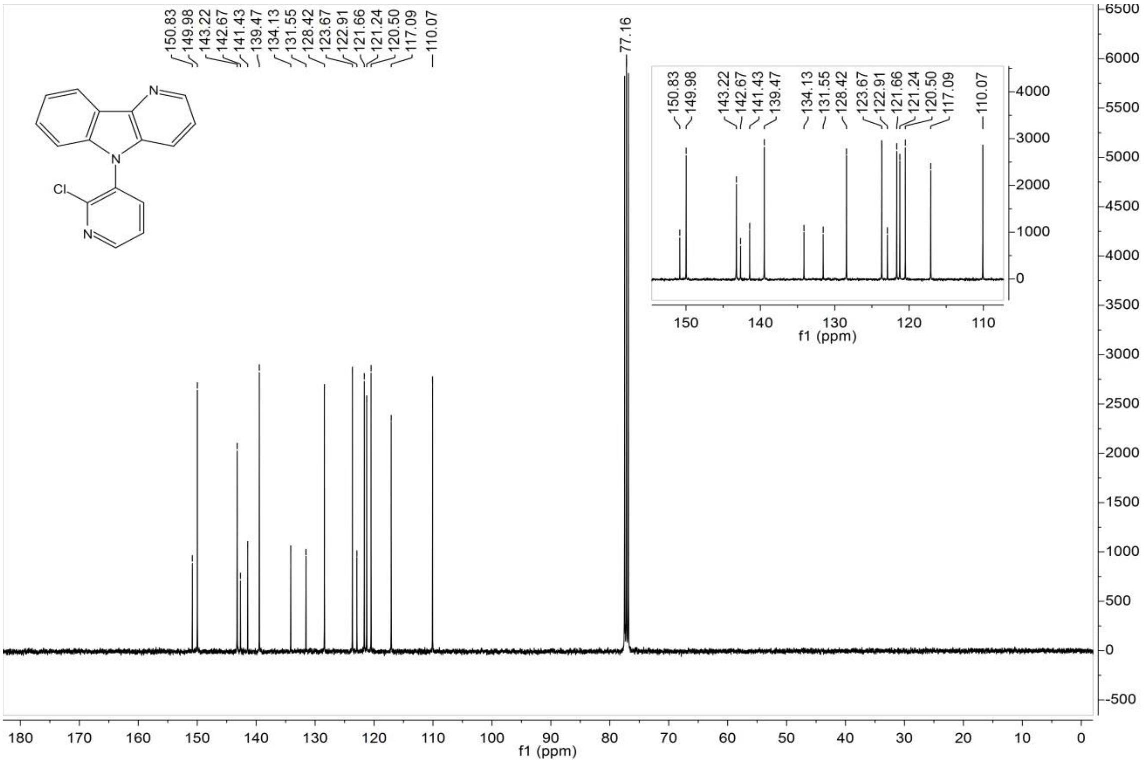

**Figure S32.** $^{13}$C NMR spectrum of **4,12PyCb** in $CDCl_3$.

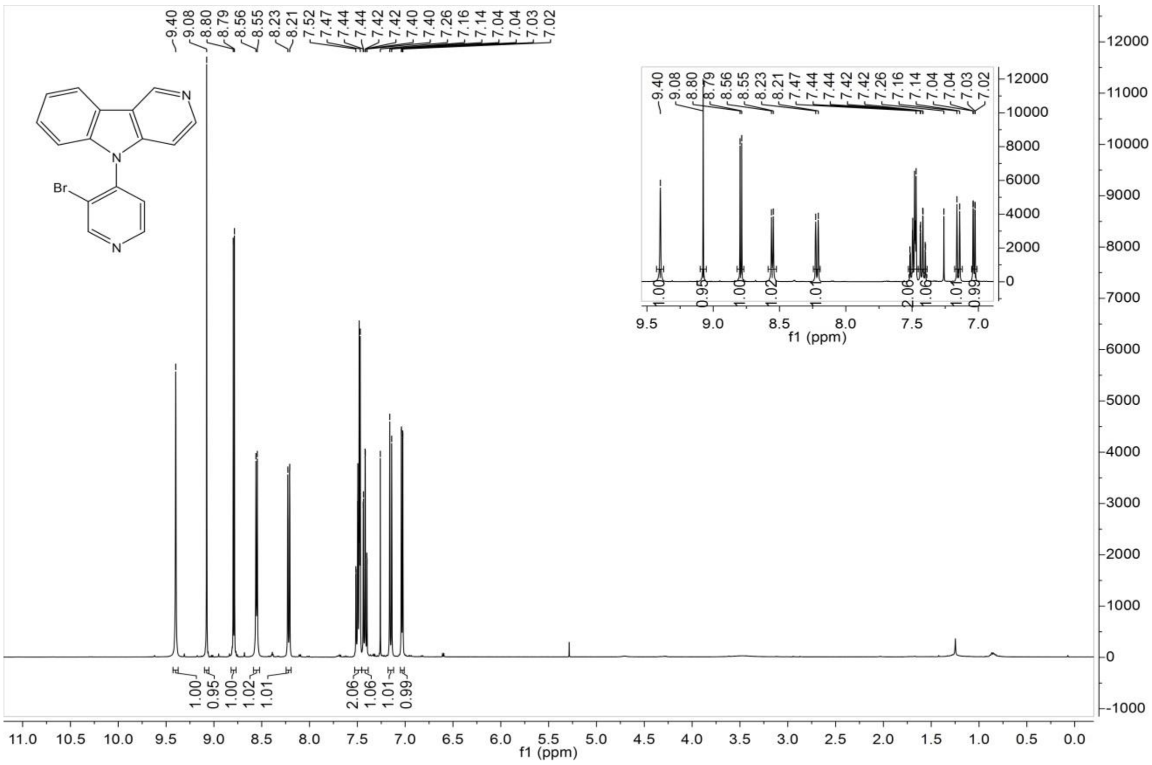


**Figure S33.** $^{1}$H NMR spectrum of **5,11PyCb** in $CDCl_3$.

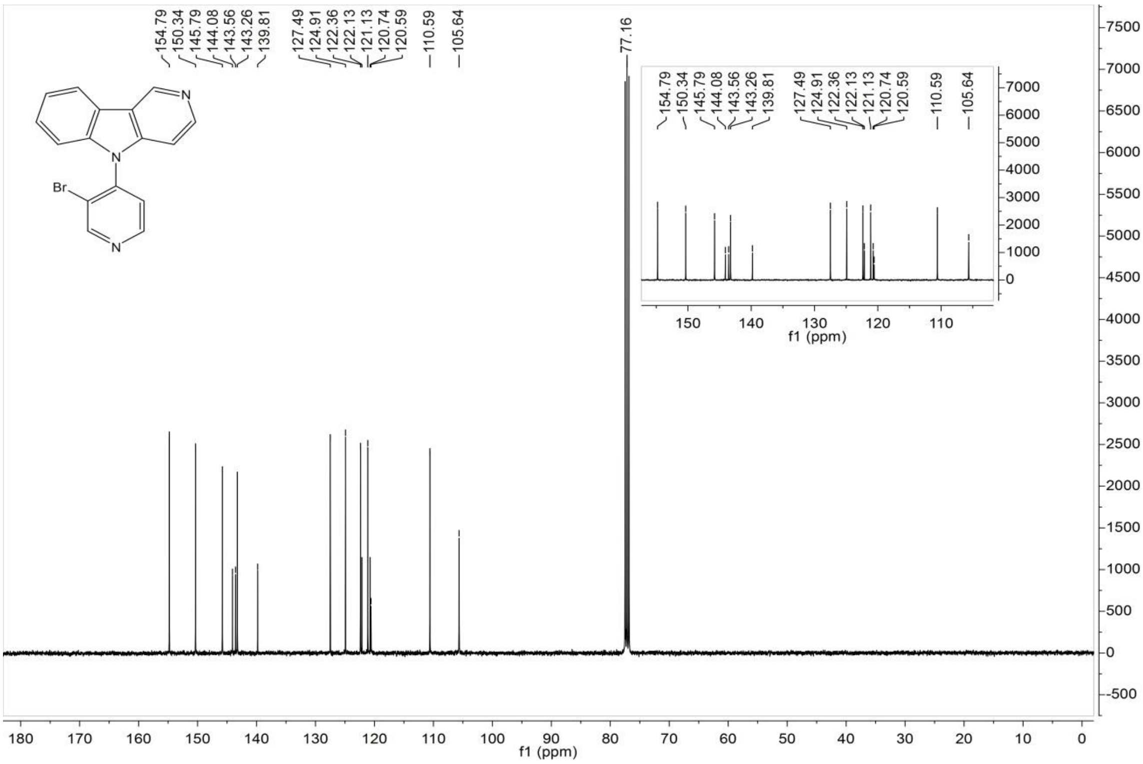


**Figure S34.** $^{13}$C NMR spectrum of **5,11PyCb** in $CDCl_3$.

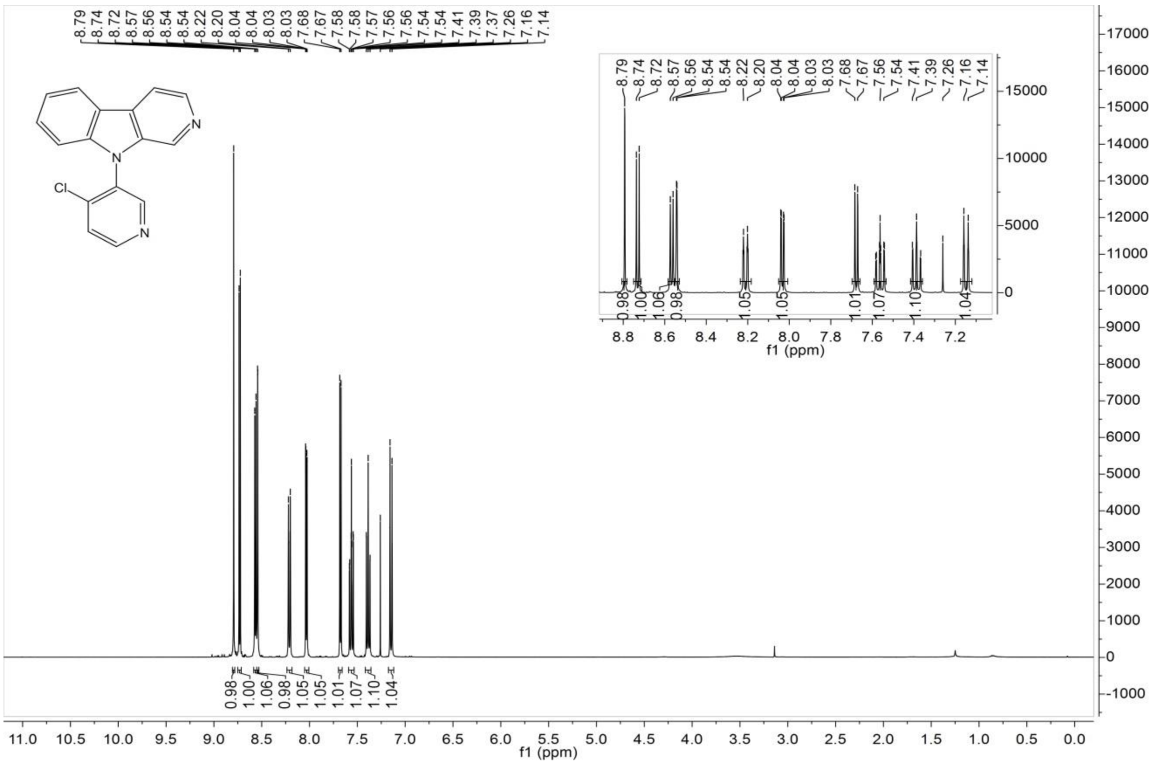

**Figure S35.** $^{1}$H NMR spectrum of **6,10PyCb** in $CDCl_3$.

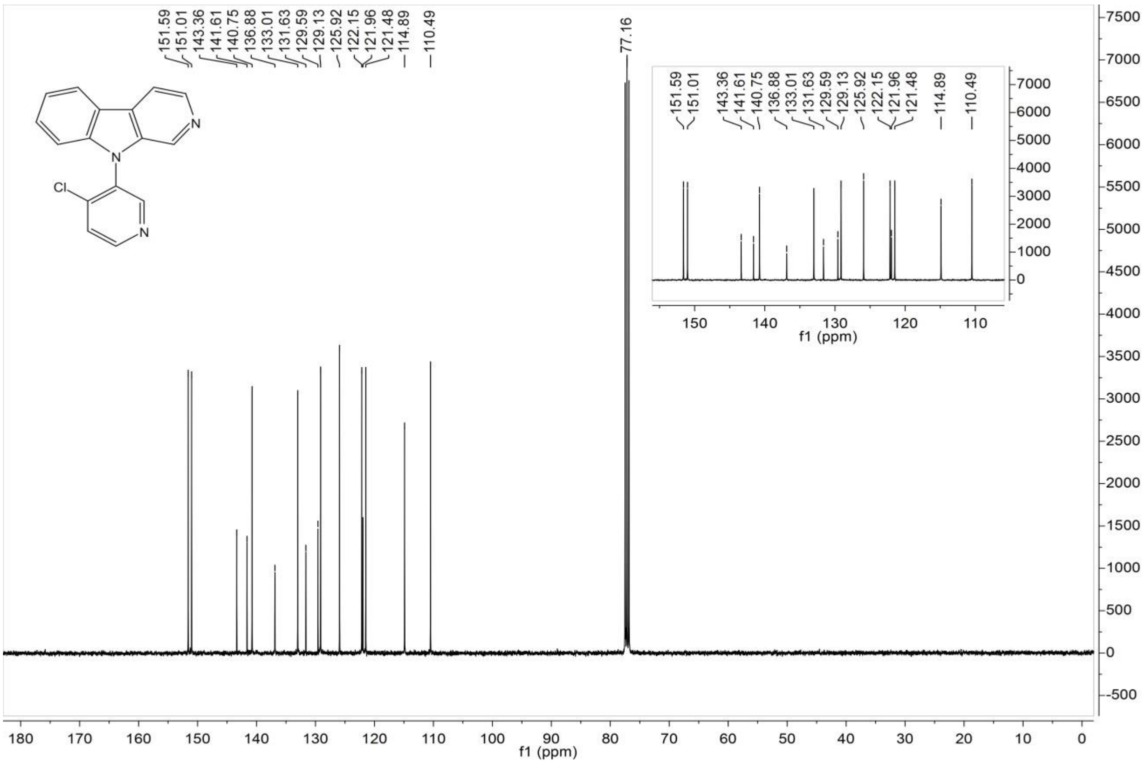

**Figure S36.** $^{13}$C NMR spectrum of **6,10PyCb** in $CDCl_3$.

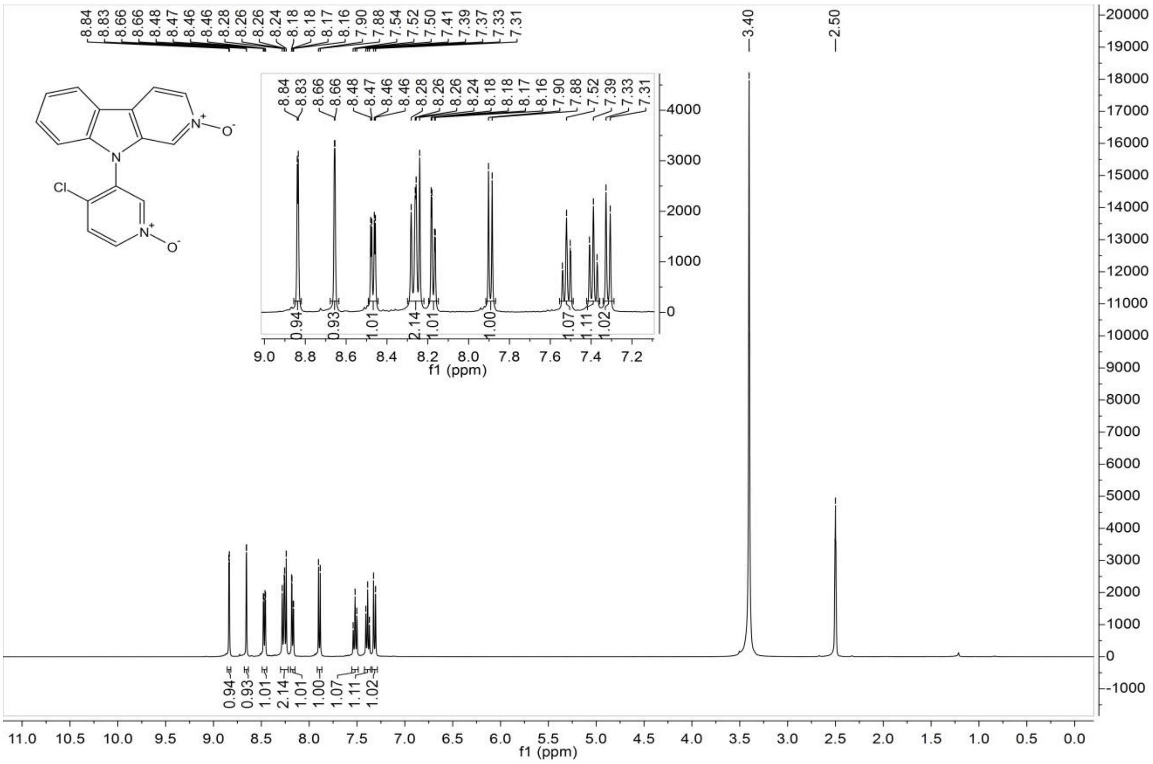

**Figure S37.** $^{1}$H NMR spectrum of **6,10PyCb-Ox** in DMSO.

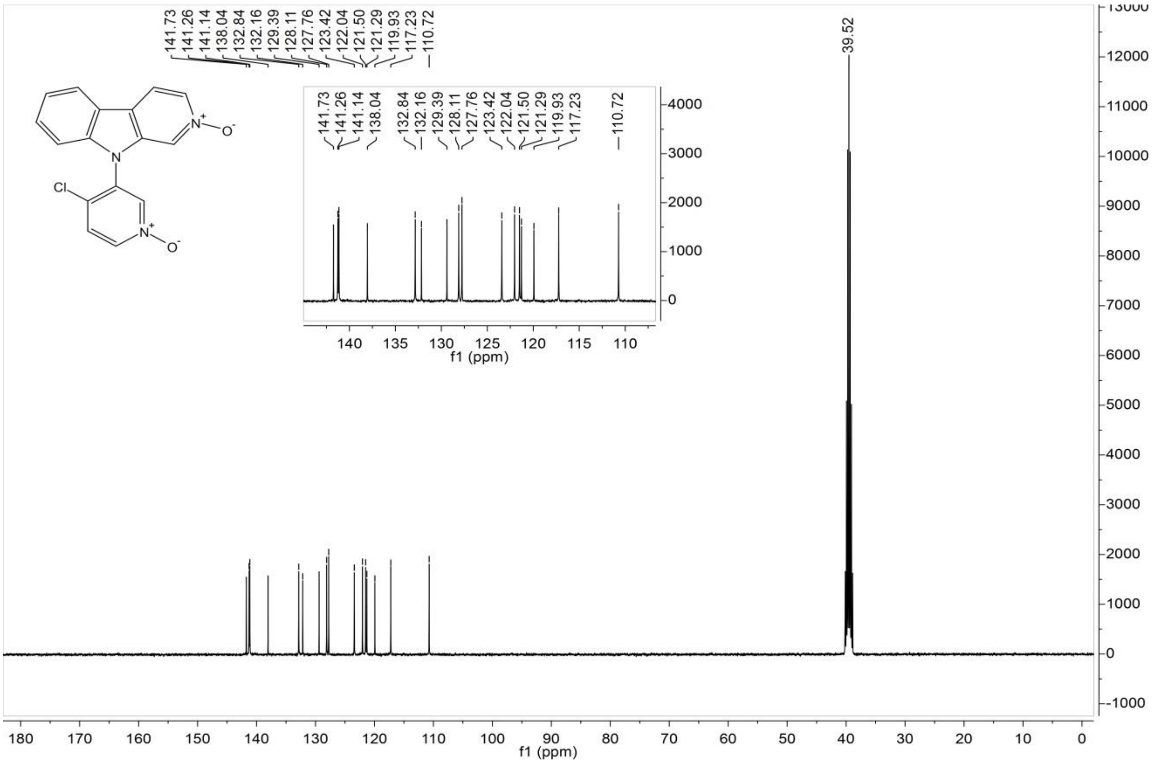

**Figure S38.** $^{13}$C NMR spectrum of **6,10PyCb-Ox** in DMSO.

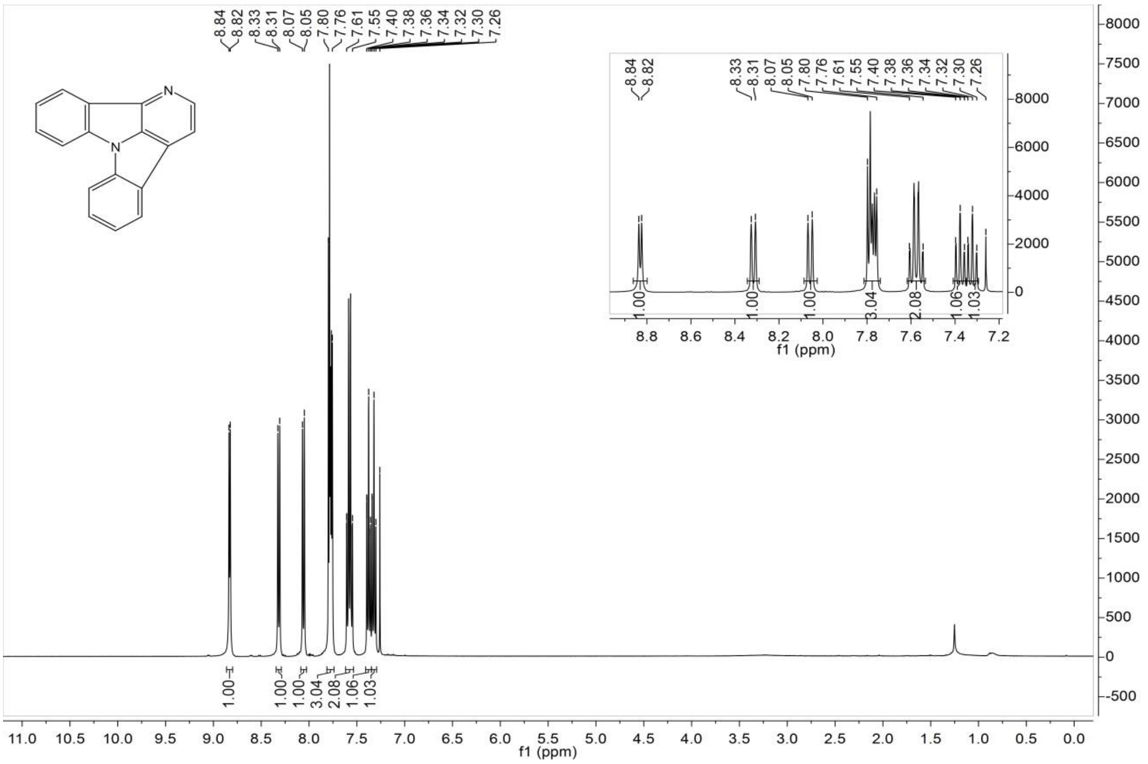

**Figure S39.** $^{1}$H NMR spectrum of **1NICz** in $CDCl_3$.

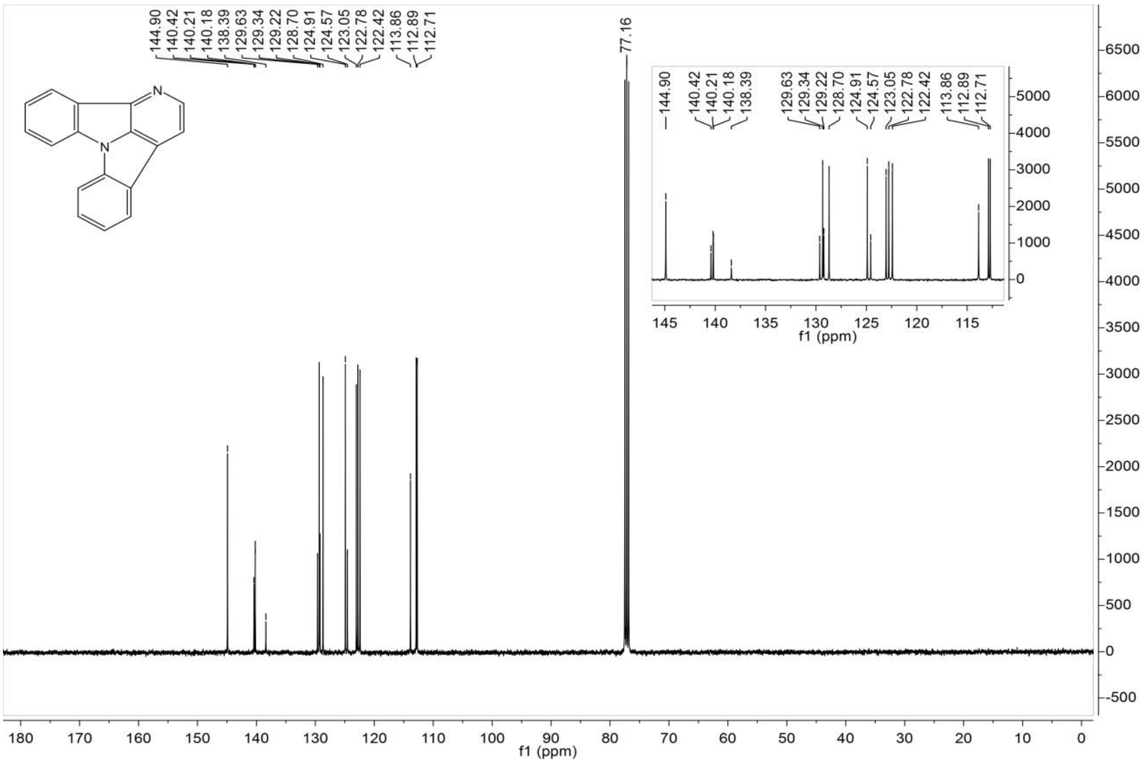

**Figure S40.** $^{13}$C NMR spectrum of **1NICz** in $CDCl_3$.

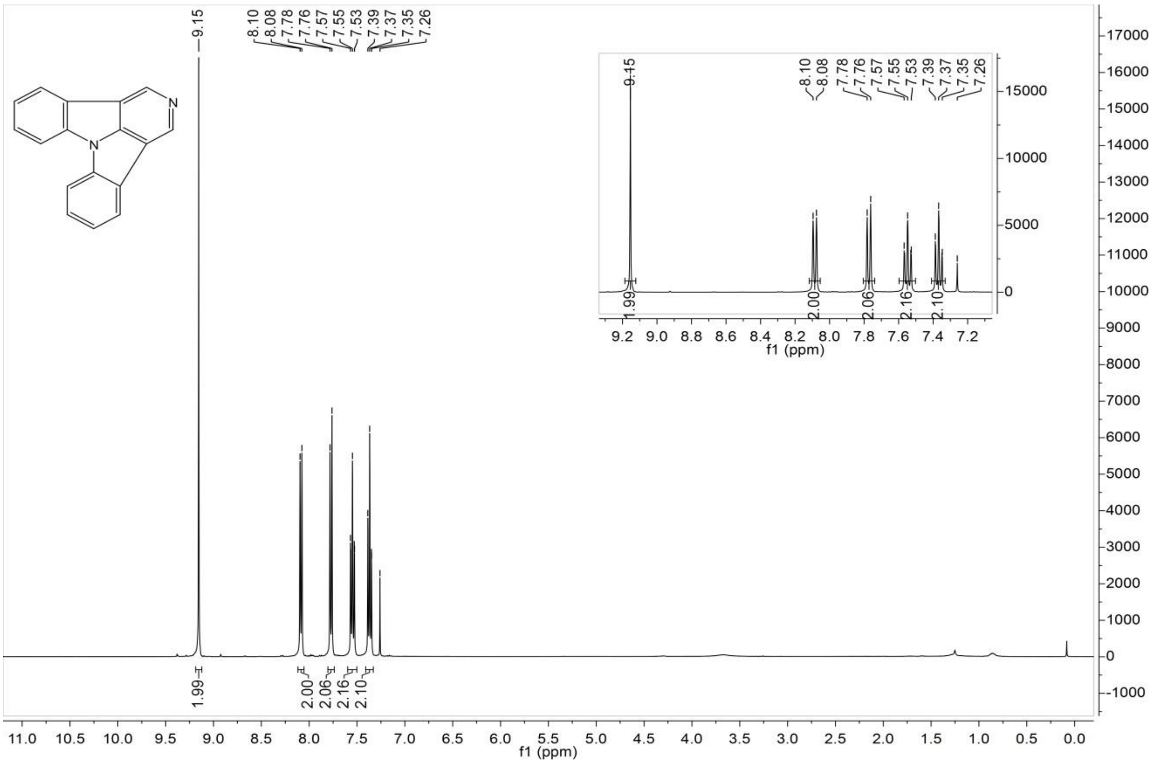


**Figure S41.** $^{1}$H NMR spectrum of **2NICz** in $CDCl_3$.

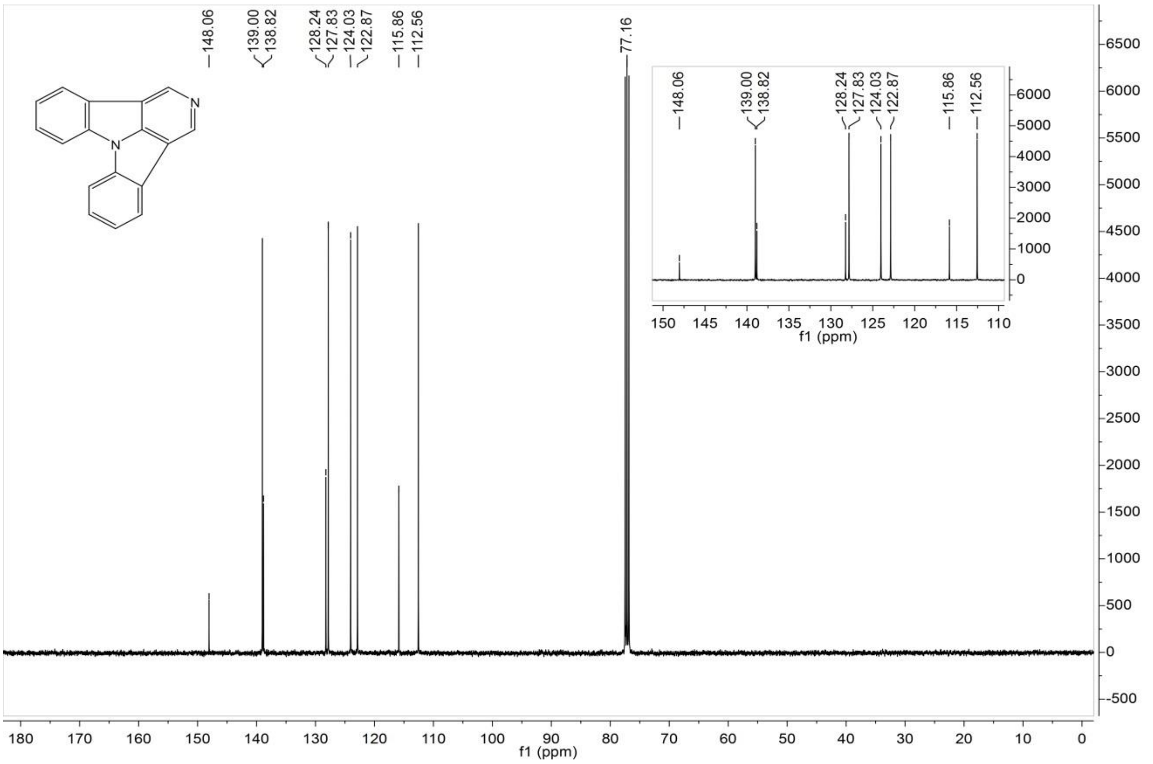


**Figure S42.** $^{13}$C NMR spectrum of **2NICz** in $CDCl_3$.

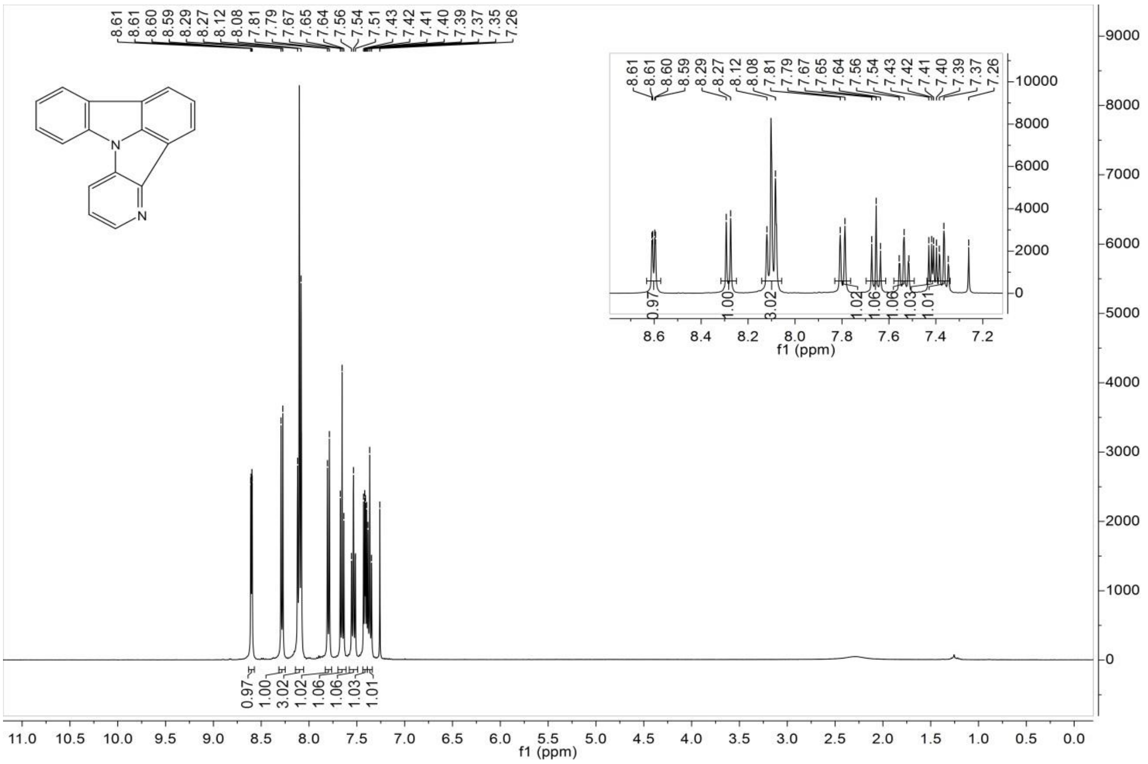

**Figure S43.** $^{1}$H NMR spectrum of **4NICz** in $CDCl_3$.

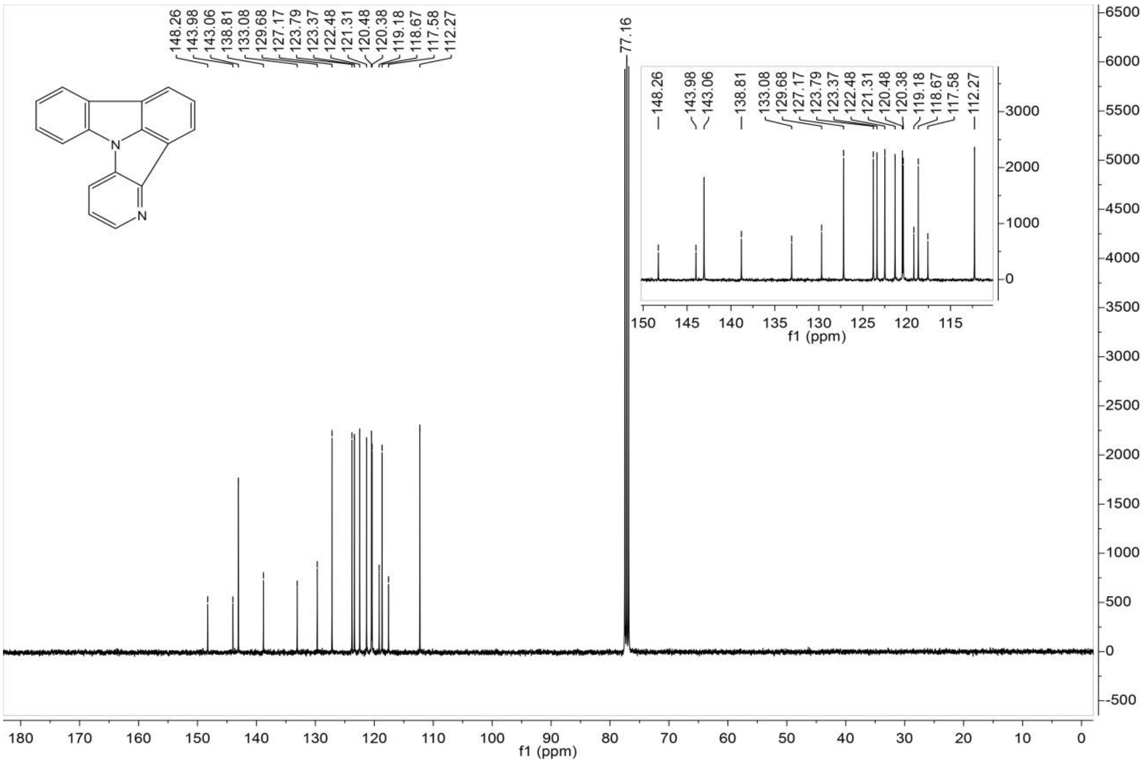

**Figure S44.** $^{13}$C NMR spectrum of **4NICz** in $CDCl_3$.

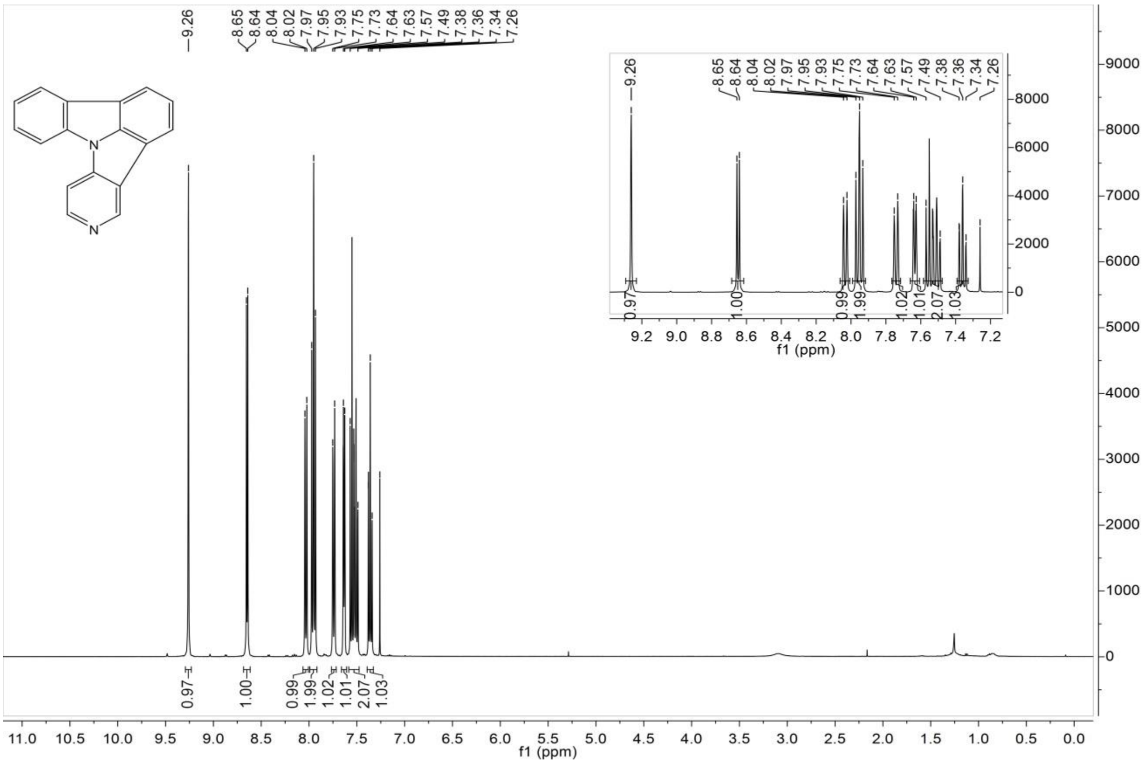

**Figure S45.** $^{1}$H NMR spectrum of **5NICz** in $CDCl_3$.

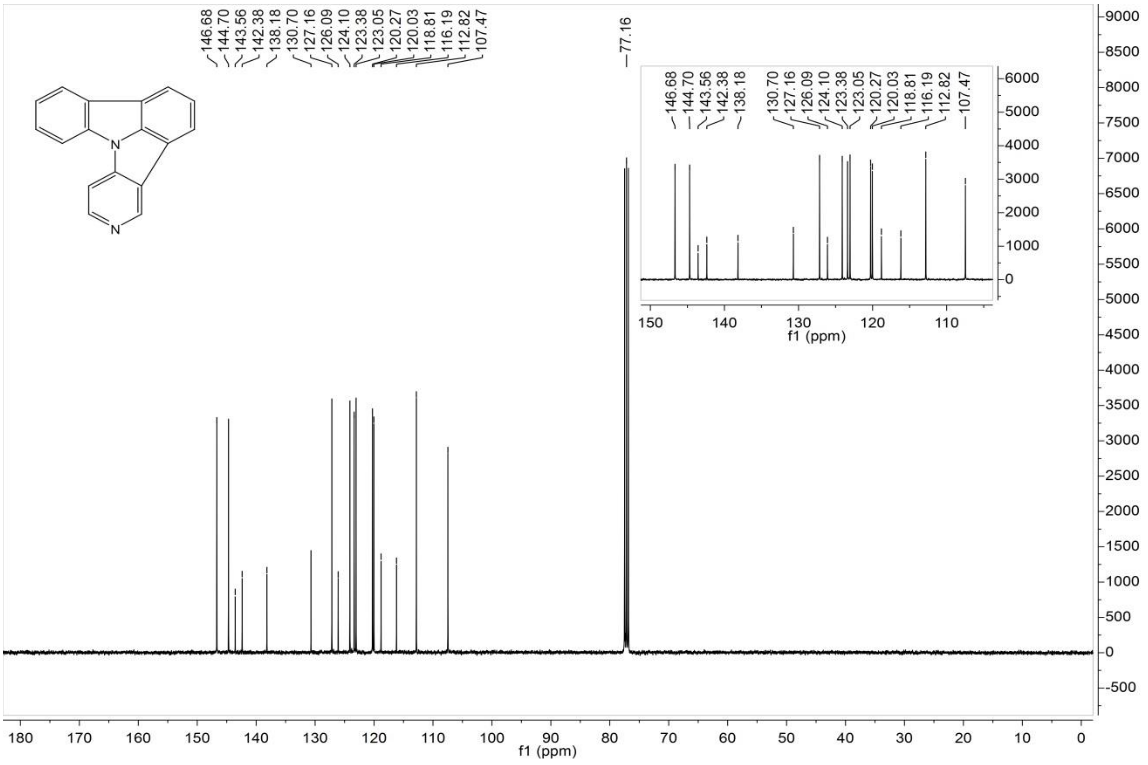

**Figure S46.** $^{13}$C NMR spectrum of **5NICz** in $CDCl_3$.

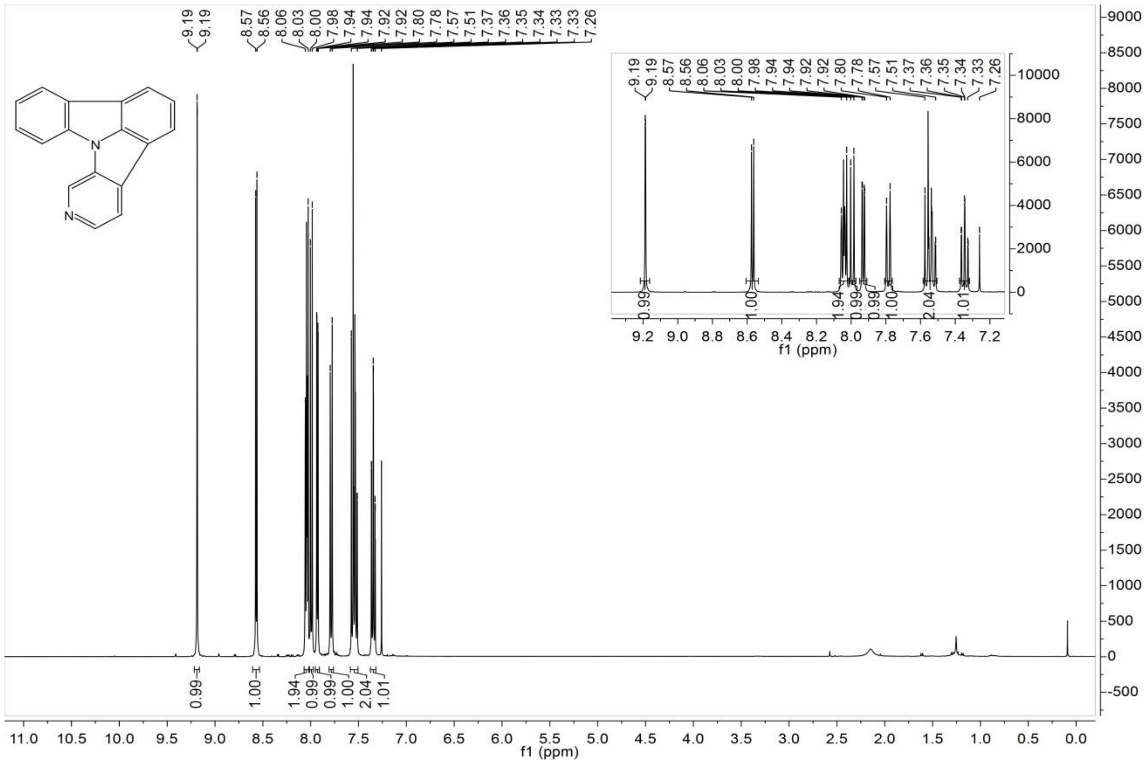

**Figure S47.** $^{1}H$ NMR spectrum of **6NICz** in $CDCl_3$.

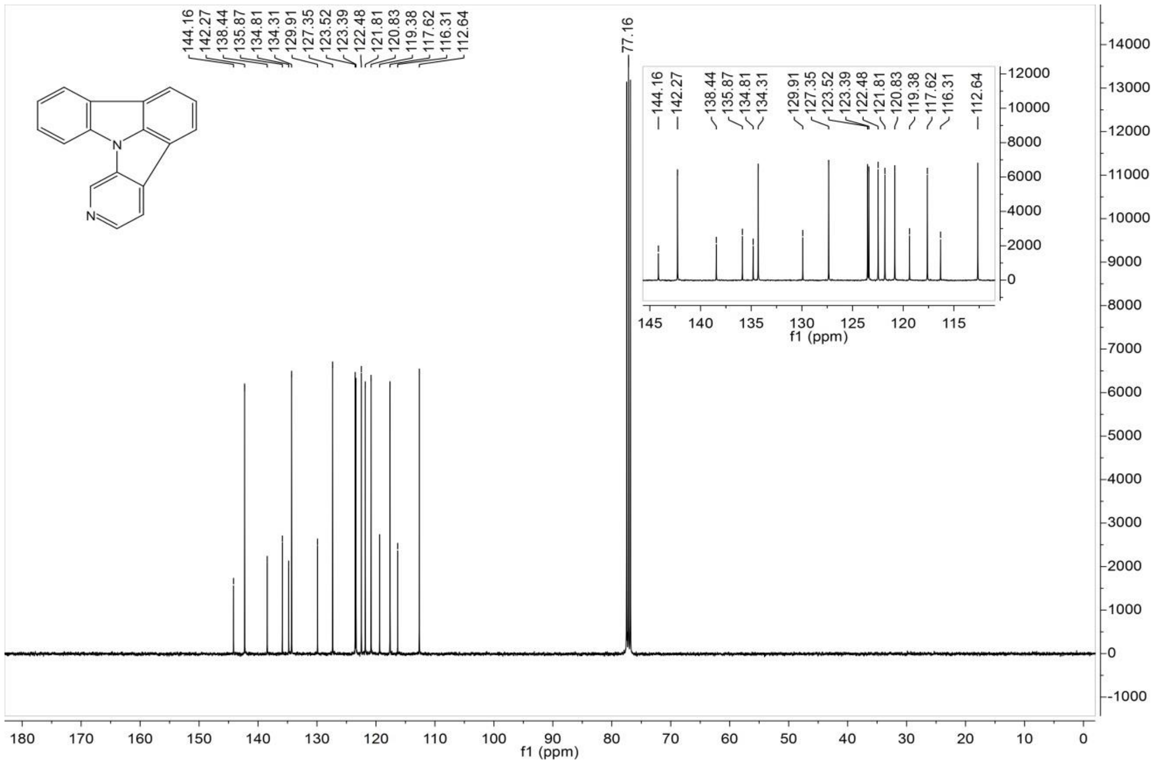

**Figure S48.** $^{13}C$ NMR spectrum of **6NICz** in $CDCl_3$.

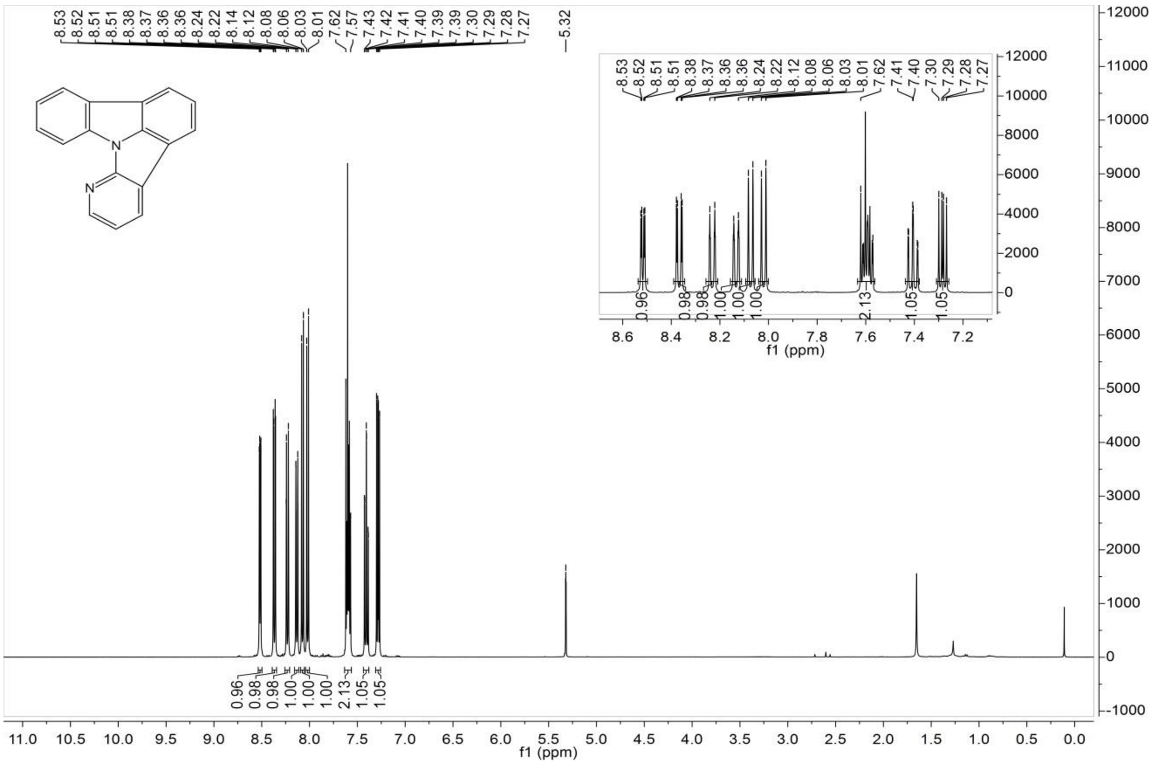


**Figure S49.** $^{1}H$ NMR spectrum of **7NICz** in $CD_2Cl_2$.

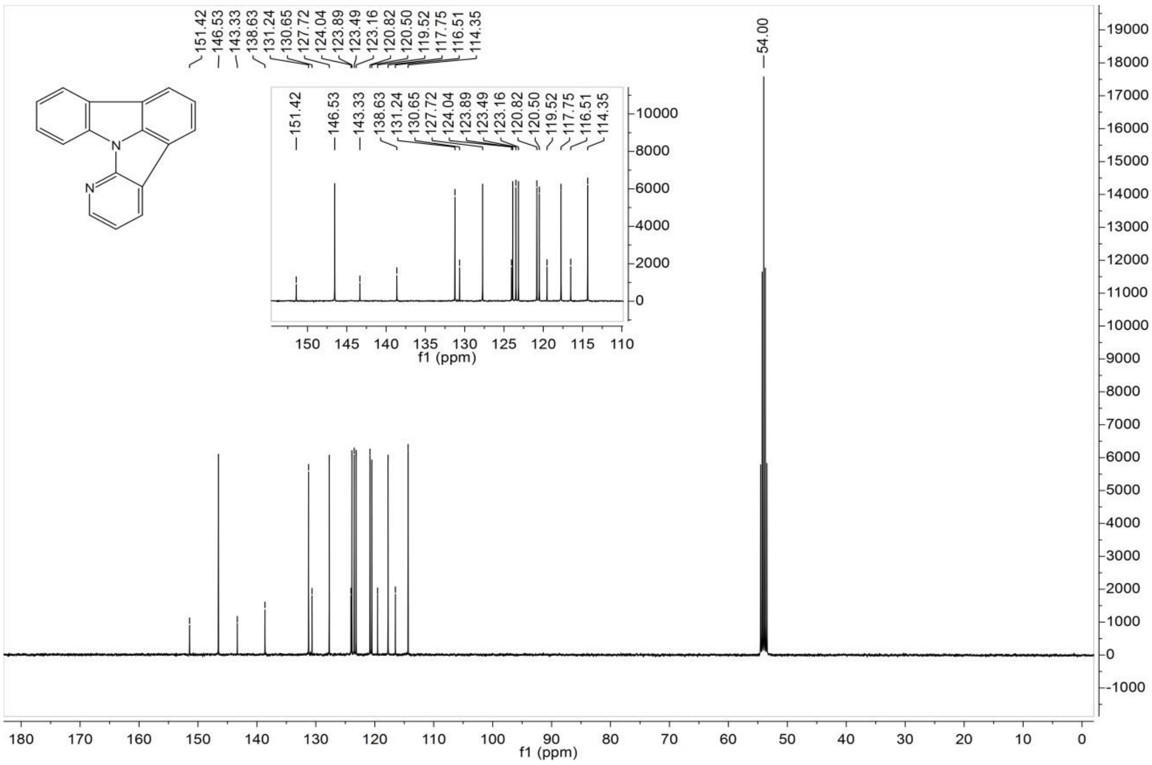


**Figure S50.** $^{13}C$ NMR spectrum of **7NICz** in $CD_2Cl_2$.

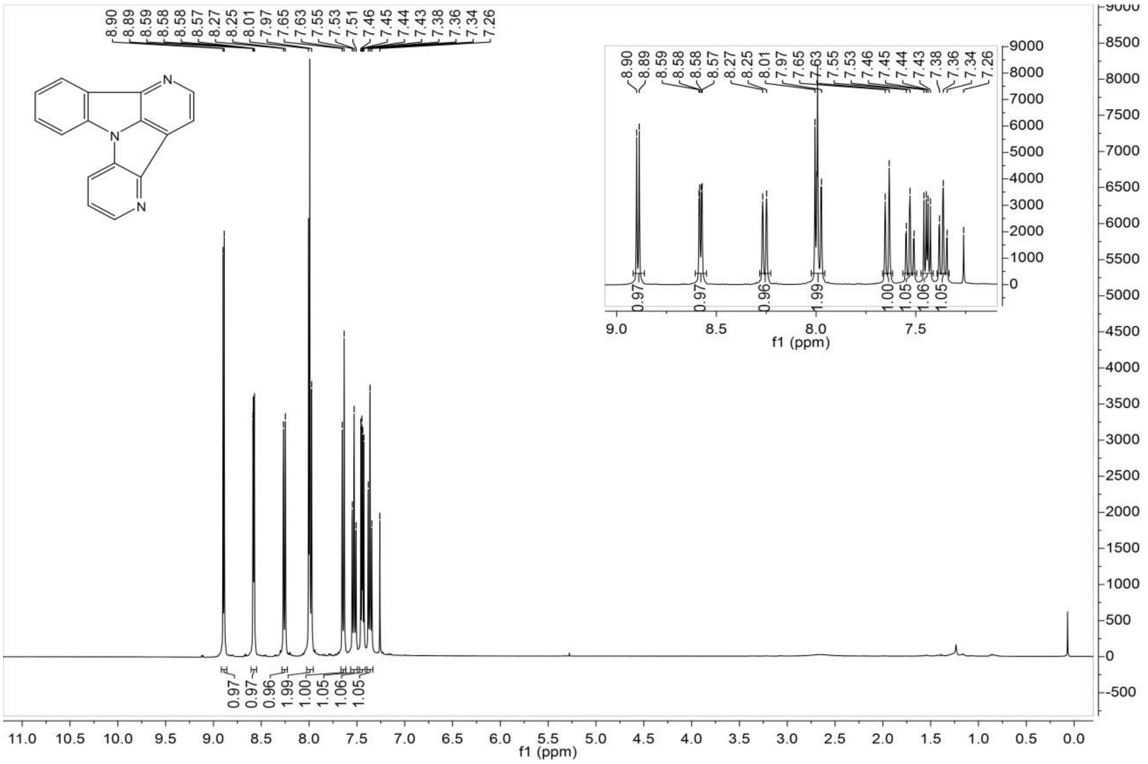


**Figure S51.** $^{1}H$ NMR spectrum of **1,4NICz** in $CDCl_3$.

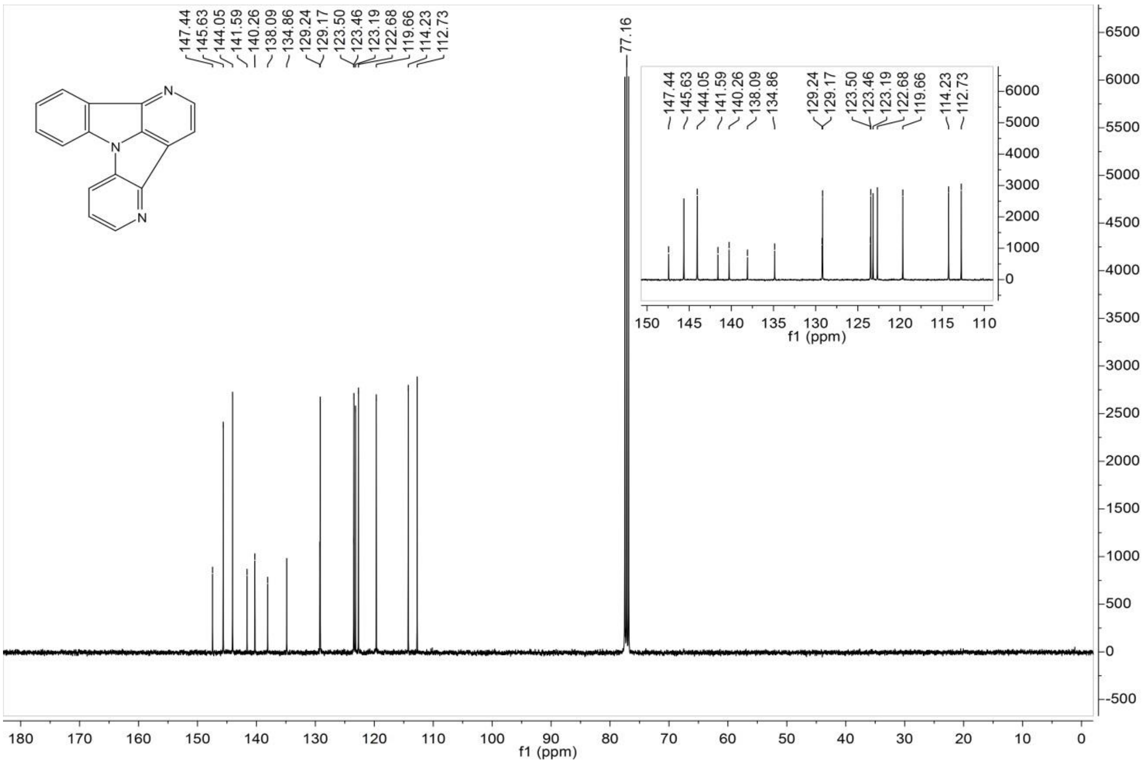


**Figure S52.** $^{13}C$ NMR spectrum of **1,4NICz** in $CDCl_3$.

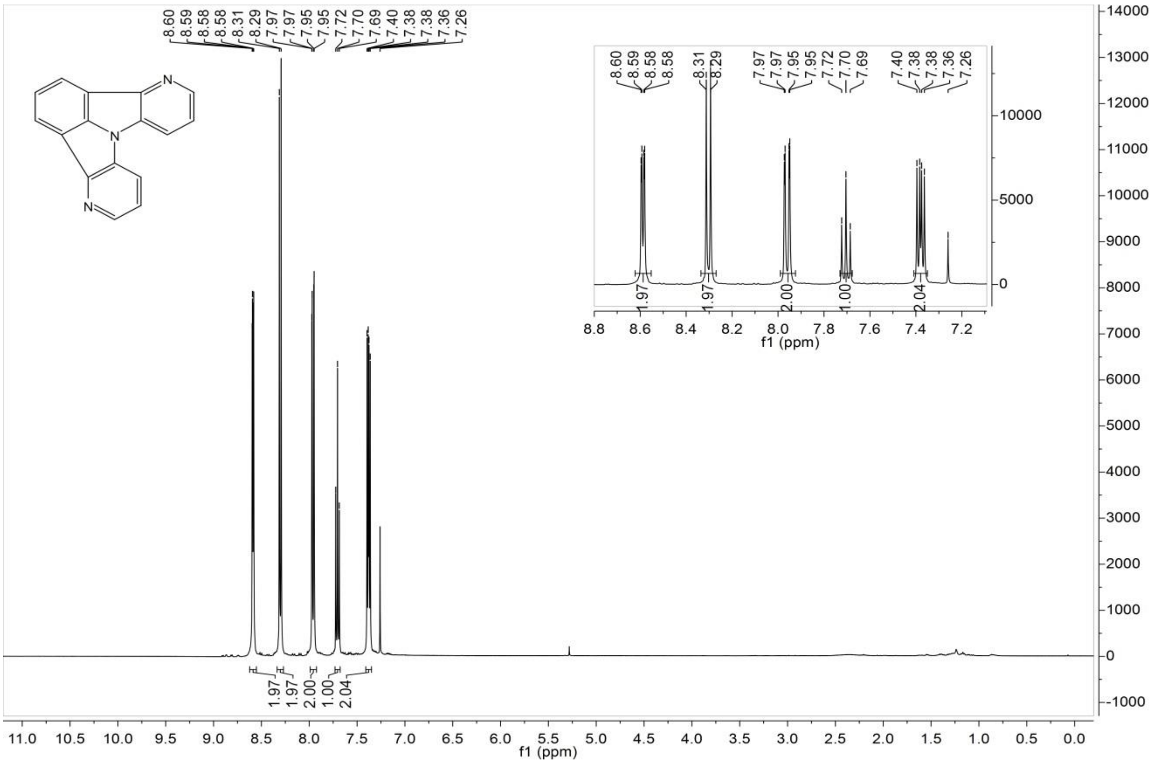

**Figure S53.** $^{1}$H NMR spectrum of **4,12NICz** in $CDCl_3$.

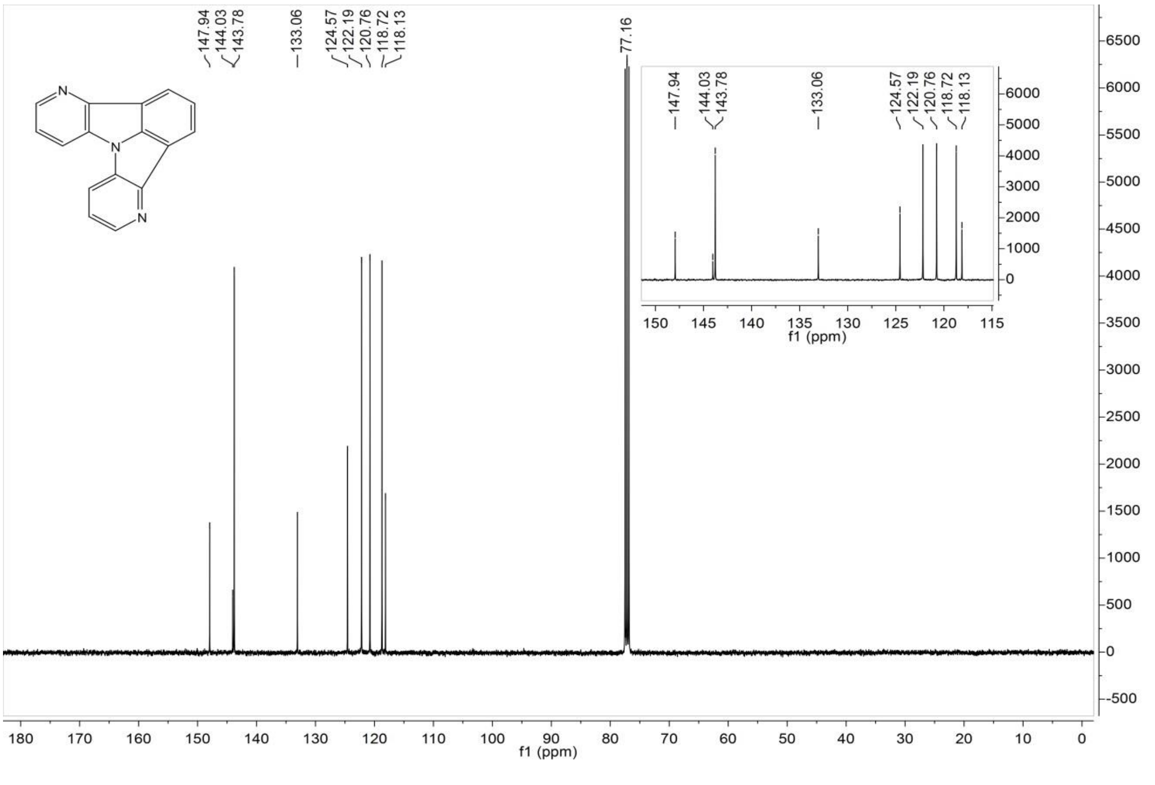

**Figure S54.** $^{13}$C NMR spectrum of **4,12NICz** in $CDCl_3$.

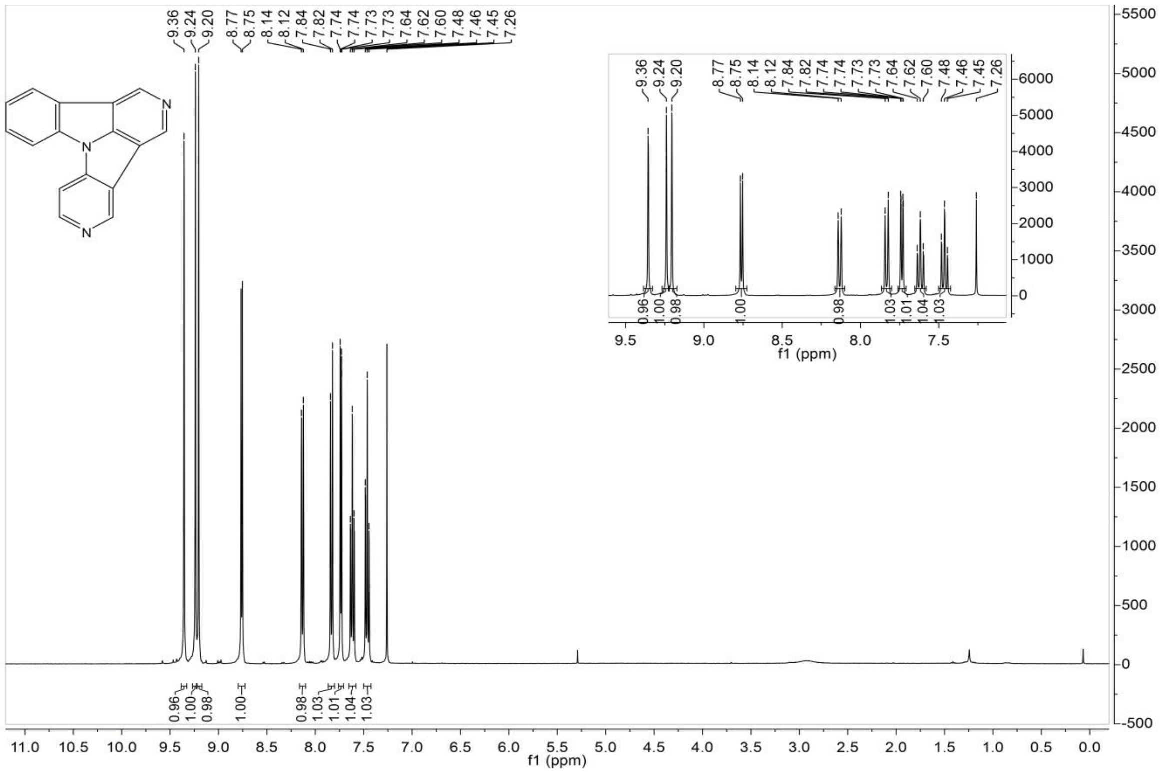

**Figure S55.** $^{1}H$ NMR spectrum of **2,5NICz** in $CDCl_3$.

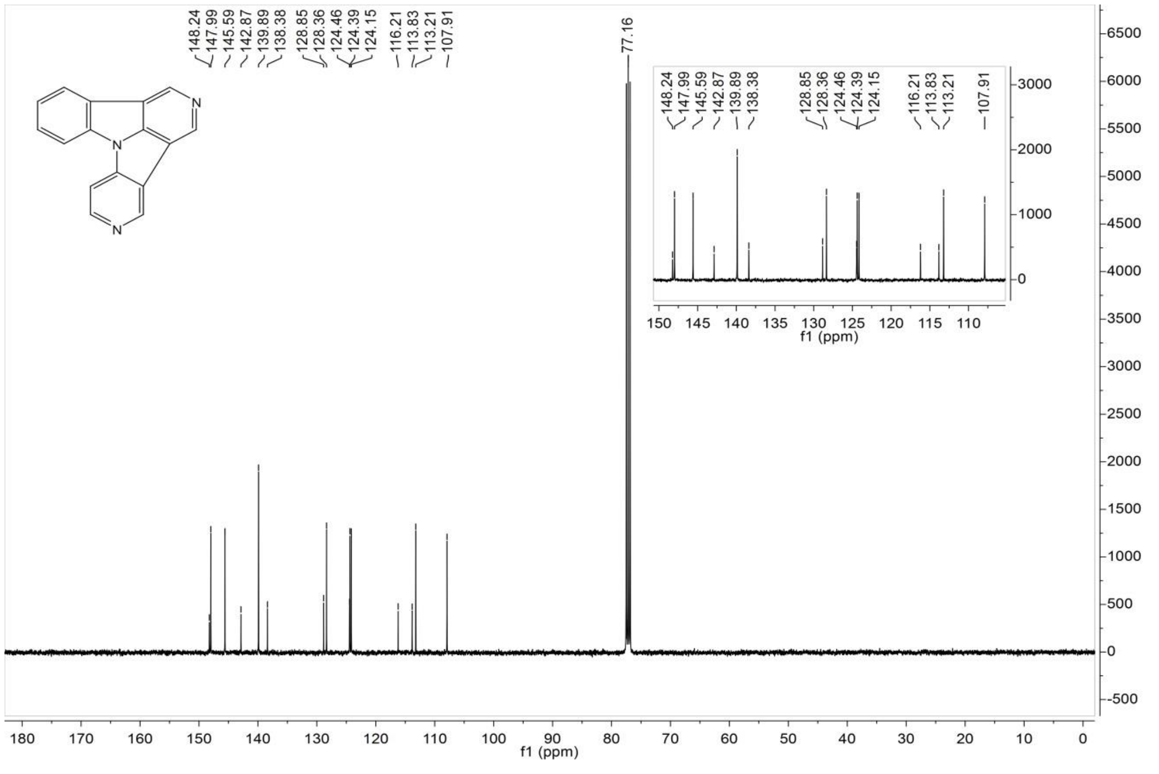

**Figure S56.** $^{13}C$ NMR spectrum of **2,5NICz** in $CDCl_3$.

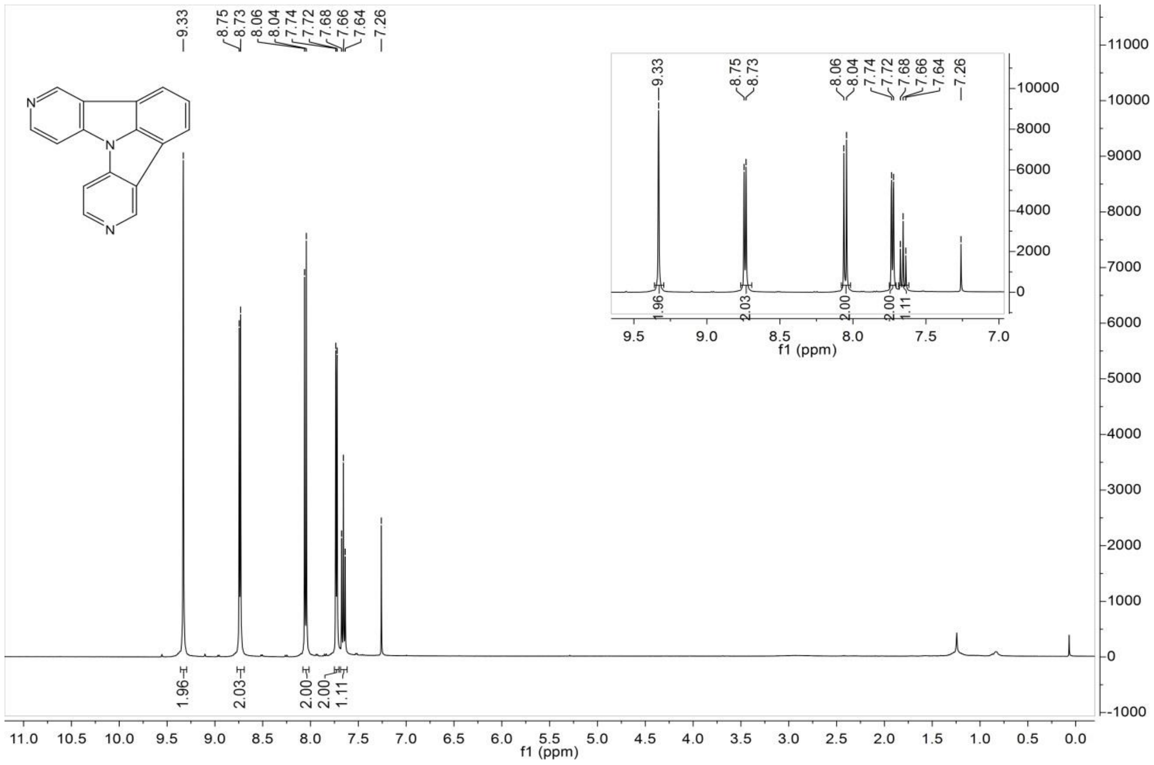


**Figure S57.** $^{1}$H NMR spectrum of **5,11NICz** in $CDCl_3$.

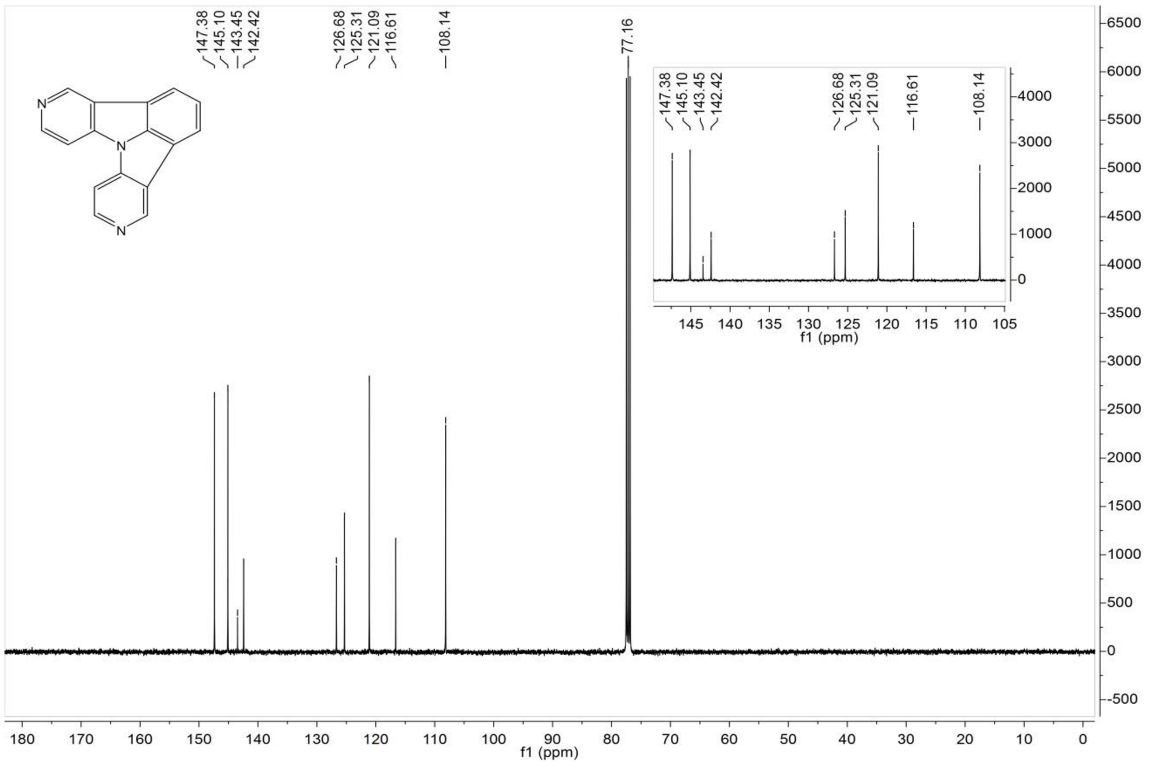


**Figure S58.** $^{13}$C NMR spectrum of **5,11NICz** in $CDCl_3$.

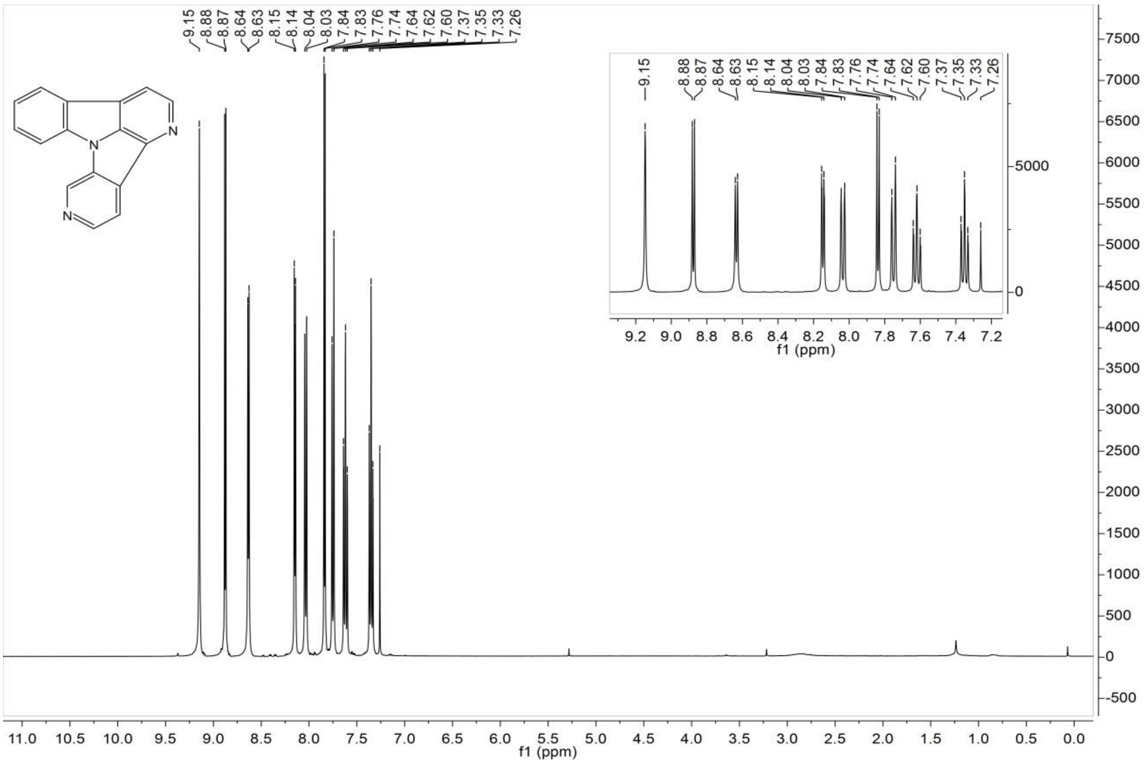

**Figure S59.** $^{1}$H NMR spectrum of **1,10NICz** in $CDCl_3$.

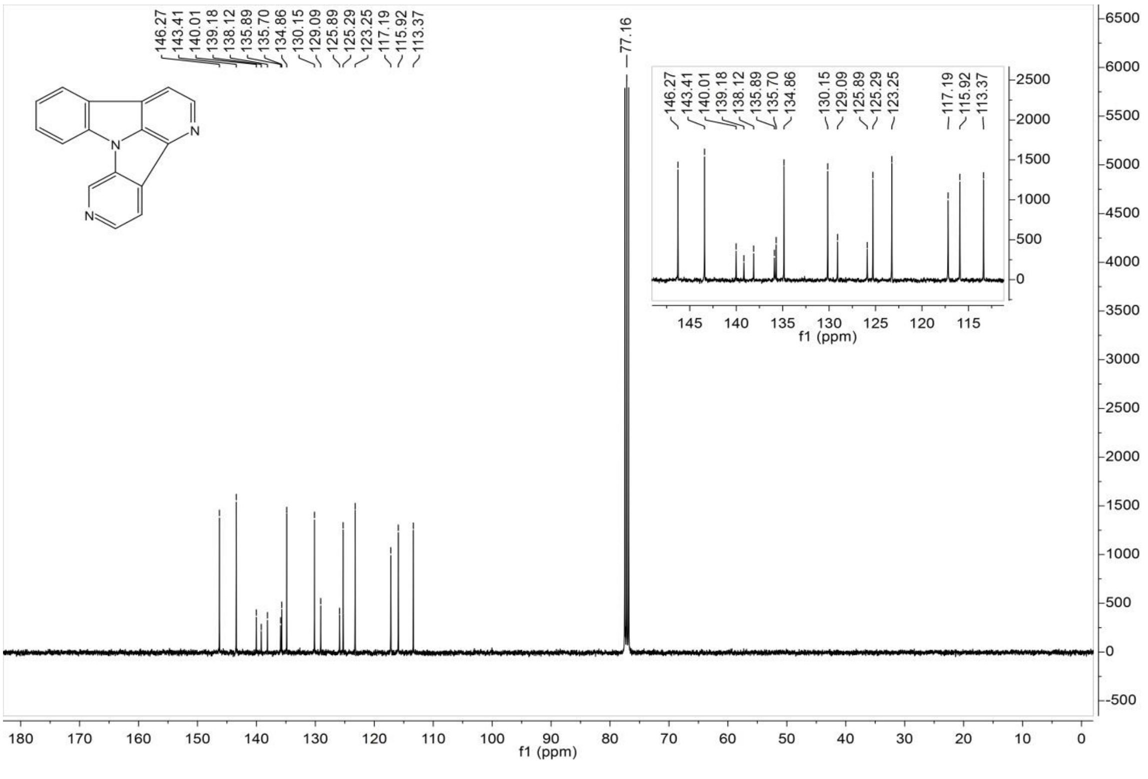

**Figure S60.** $^{13}$C NMR spectrum of **1,10NICz** in $CDCl_3$.

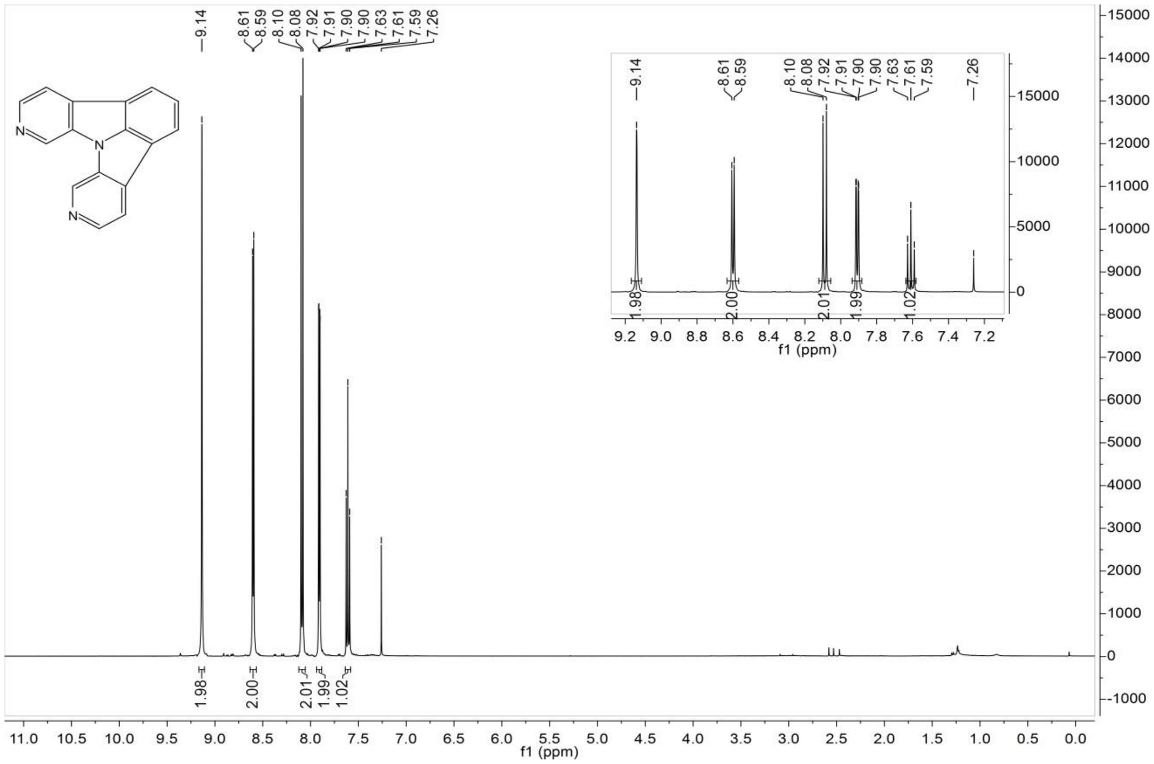

**Figure S61.** $^{1}$H NMR spectrum of **6,10NICz** in $CDCl_3$.

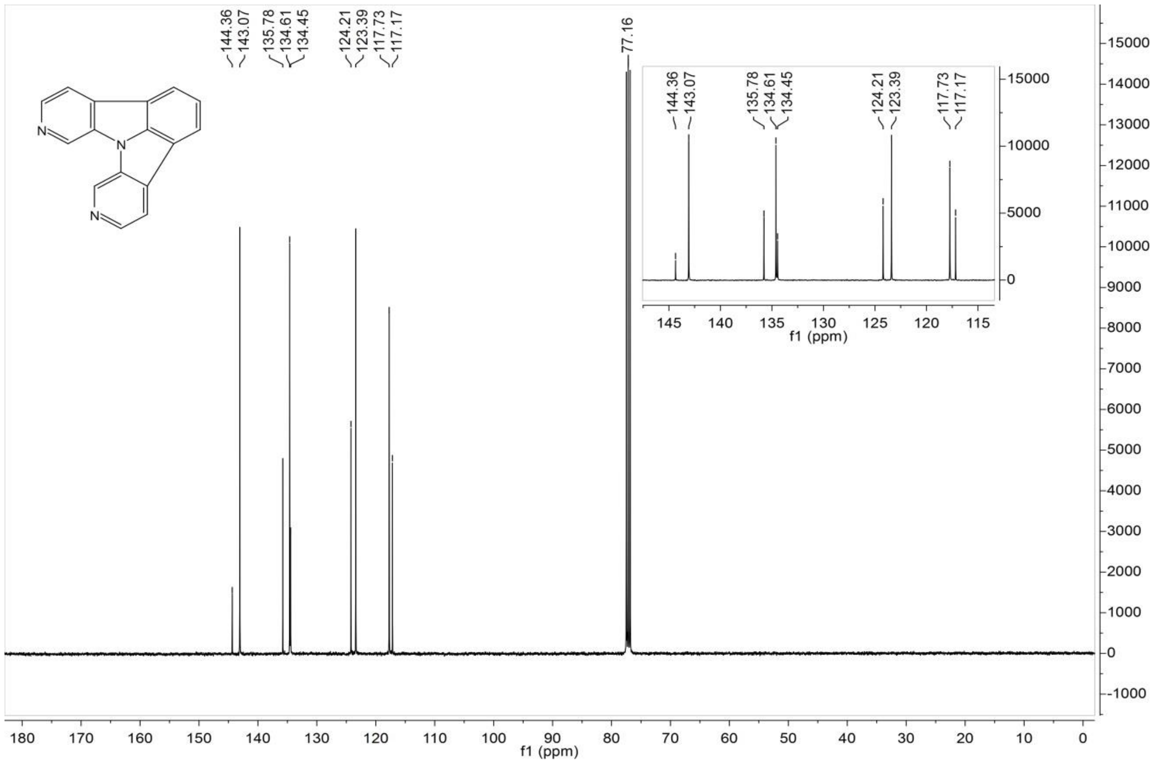

**Figure S62.** $^{13}$C NMR spectrum of **6,10NICz** in $CDCl_3$.

## 4 Additional Screening results

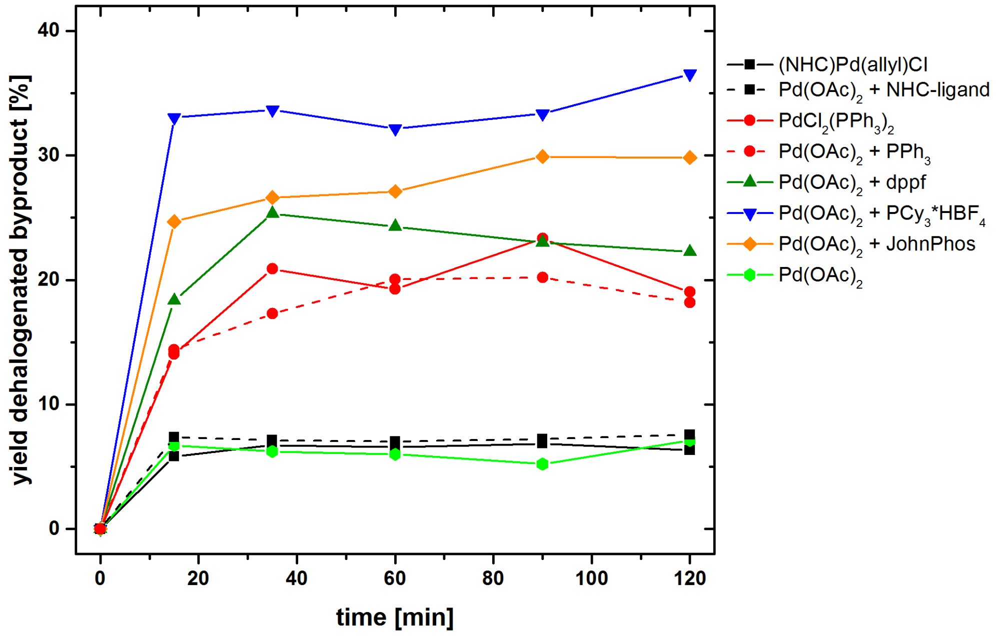


**Figure S63.** Formation of dehalogenated byproduct during C-H activation of bromo-**4PCz** towards **4NICz** applying different catalysts. Reaction conditions: bromo-**4PCz** (0.025 mmol), $K_2CO_3$ (2 eq.), catalyst (10 mol%) and ligand (12 mol%: NHC, dppf; 22 mol%: $PPh_3$, $PCy_3$*$HBF_4$, JohnPhos), DMA, 130 °C.

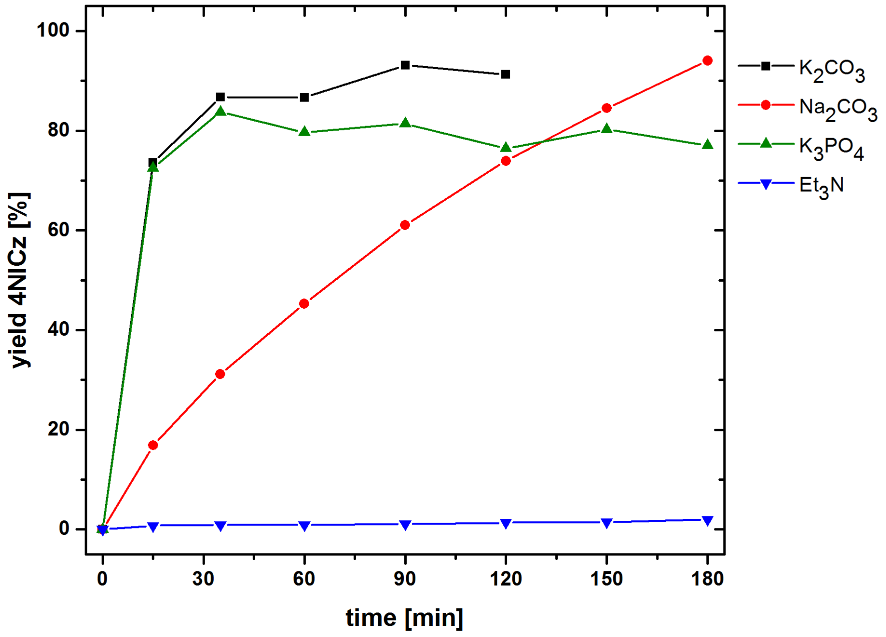


**Figure S64.** C-H activation of bromo-**4PCz** towards **4NICz** applying different bases. Reaction conditions: bromo-**4PCz** (0.025 mmol), base (2 eq.), (NHC)Pd(allyl)Cl (10 mol%), DMA, 130 °C.

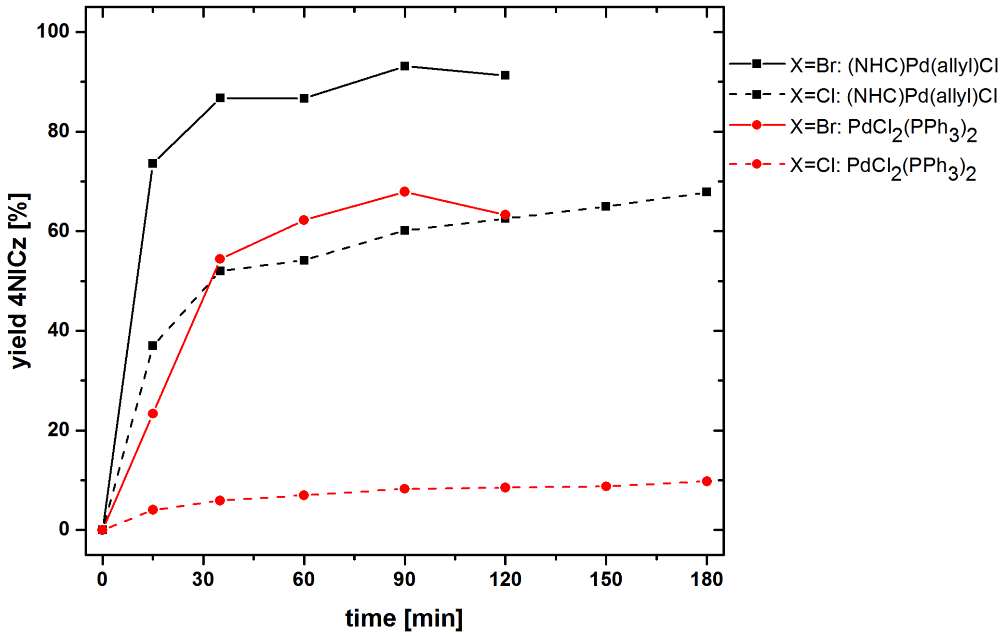


**Figure S65.** C-H activation towards **4NICz** applying bromine and chlorine precursor. Reaction conditions: **4PCz** (0.025 mmol), $K_2CO_3$ (2 eq.), (NHC)Pd(allyl)Cl (10 mol%), DMA, 130 °C.

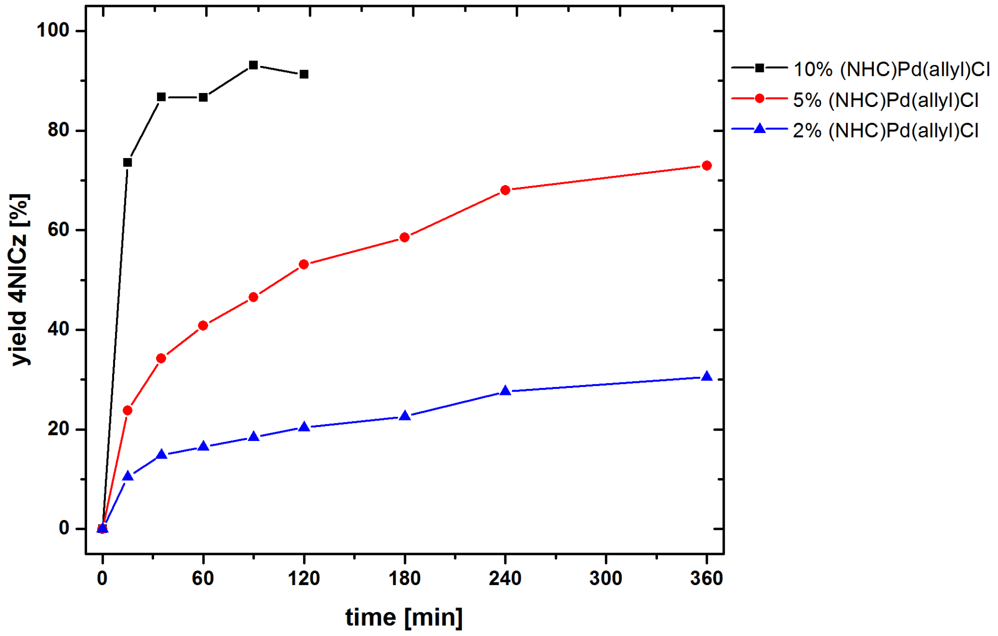


**Figure S66.** C-H activation of bromo-**4PCz** towards **4NICz** applying different catalyst amounts. Reaction conditions: bromo-**4PCz** (0.025 mmol), $K_2CO_3$ (2 eq.), (NHC)Pd(allyl)Cl (2 – 10 mol%), DMA, 130 °C.

# 5 Molar attenuation coefficient

**Table S1.** Molar attenuation coefficients of peak maxima and lowest energy peaks of ICz and the synthesized NICz derivatives.

| | maximum[a] | | lowest energy peak | |
|---|---|---|---|---|
| | λ [nm] | ε [L*mol$^{-1}$*cm$^{-1}$] | λ [nm] | ε [L*mol$^{-1}$*cm$^{-1}$] |
| ICz | 285 | 38100 | 363 | 11120 |
| 1NICz | 280 | 16100 | 372 | 5660 |
| 2NICz | 279 | 16400 | 344 | 2840 |
| 4NICz | 297 | 22100 | 362 | 4820 |
| 5NICz | 283 | 24860 | 351 | 19300 |
| 6NICz | 284 | 25500 | 373 | 11640 |
| 7NICz | 287 | 24220 | 357 | 7380 |
| 1,4NICz | 294 | 23620 | 372 | 4780 |
| 2,5NICz | -[b] | - | 341 | 4040 |
| 1,10NICz | 283 | 16300 | 385 | 6820 |
| 4,12NICz | 297 | 28140 | 351 | 4180 |
| 5,11NICz | 285 | 12780 | 343 | 25160 |
| 6,10NICz | 280 | 21060 | 379 | 14640 |

[a] Only peaks >270 nm considered due to possible influence of the solvent. [b] No distinct peak maximum above 270 nm observable.

# 6 Cyclic Voltammetry

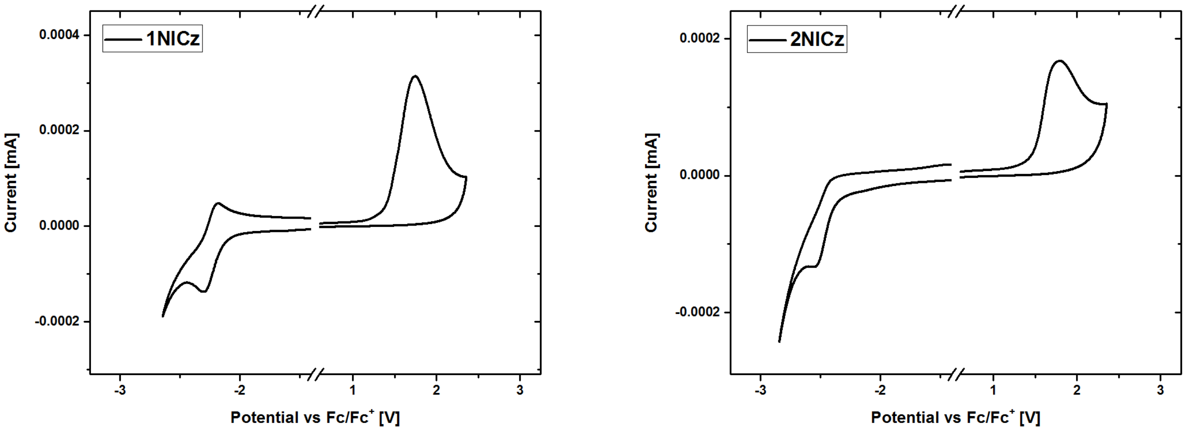


**Figure S67.** Cyclic voltammograms of **1NICz** (left) and **2NICz** (right).

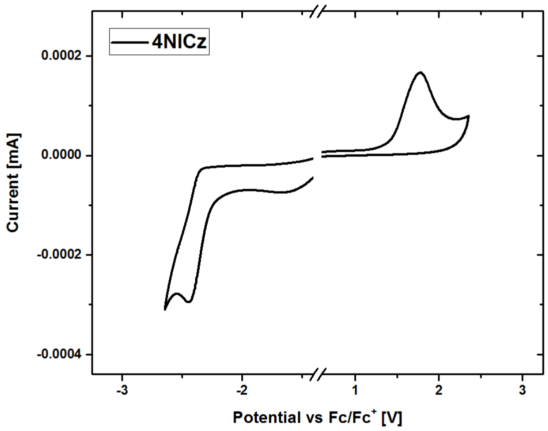


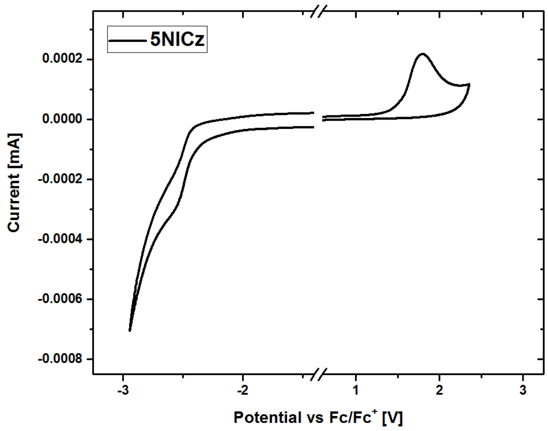


**Figure S68.** Cyclic voltammograms of **4NICz** (left) and **5NICz** (right).

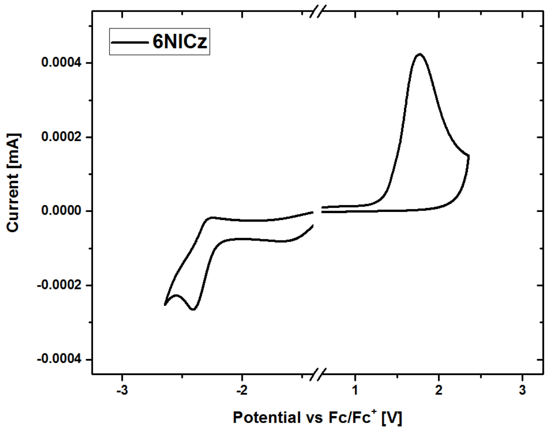


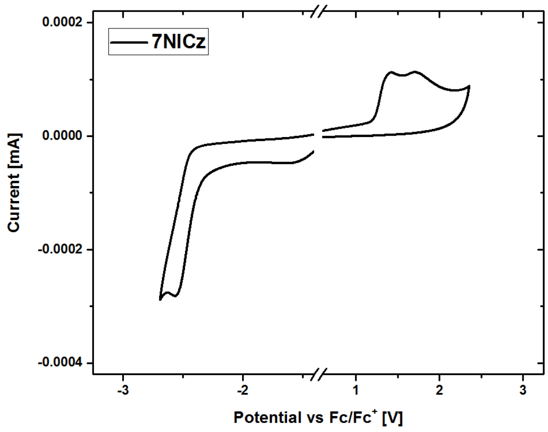


**Figure S69.** Cyclic voltammograms of **6NICz** (left) and **7NICz** (right).

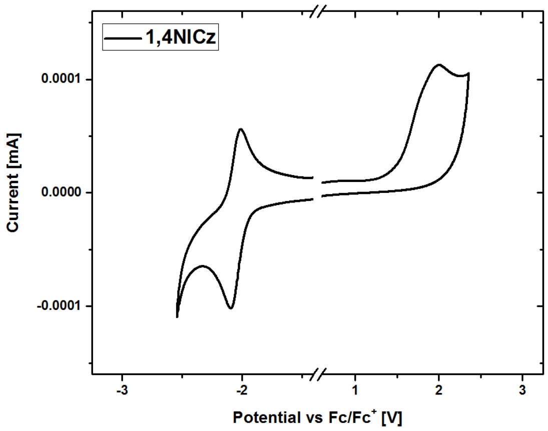


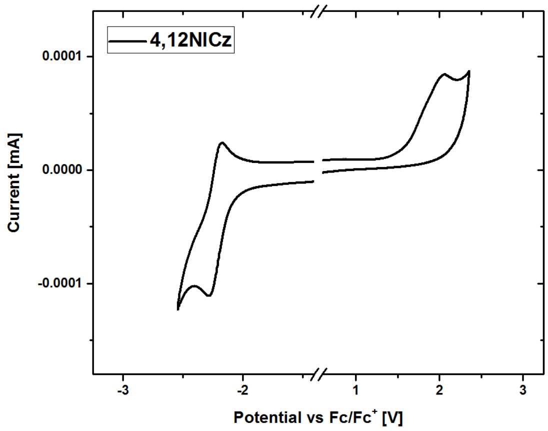


**Figure S70.** Cyclic voltammograms of **1,4NICz** (left) and **4,12NICz** (right).

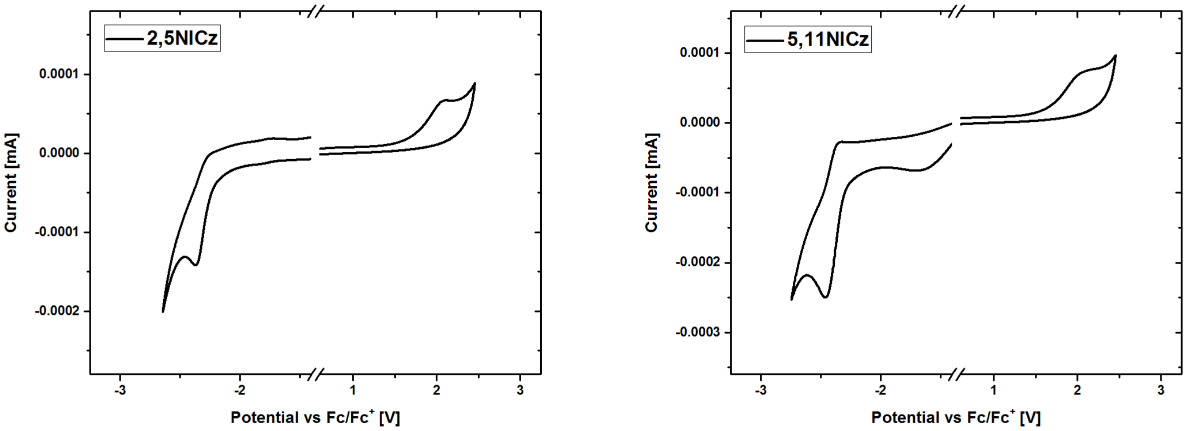


**Figure S71.** Cyclic voltammograms of **2,5NICz** (left) and **5,11NICz** (right).

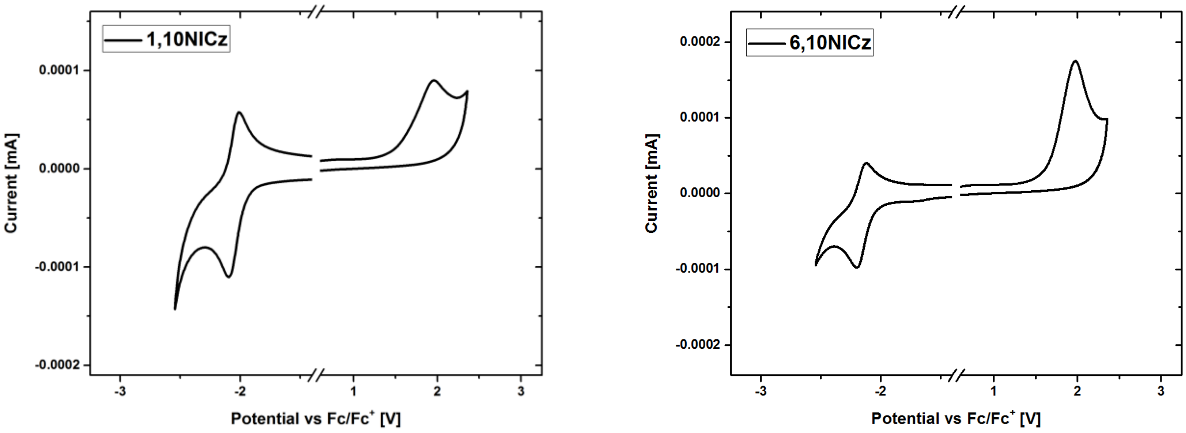


**Figure S72.** Cyclic voltammograms of **1,10NICz** (left) and **6,10NICz** (right).

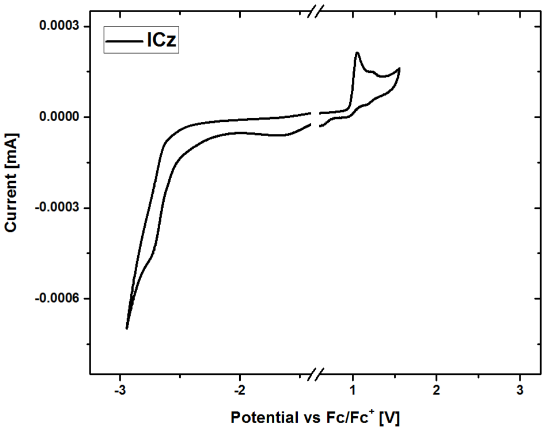


**Figure S73.** Cyclic voltammogram of **ICz**.

# 7 HOMO / LUMO energy levels

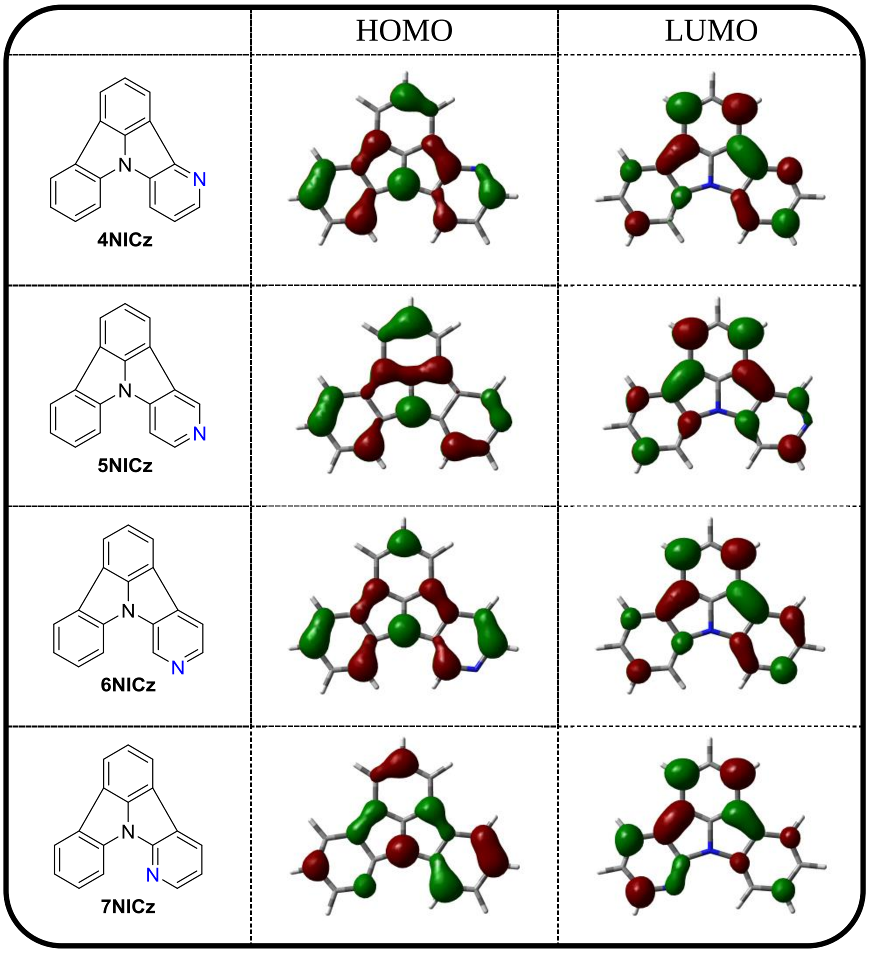


**Figure S74.** Spatial distribution of the HOMO and LUMO levels of mono substituted isomers **4NICz**, **5NICz**, **6NICz** and **7NICz**.

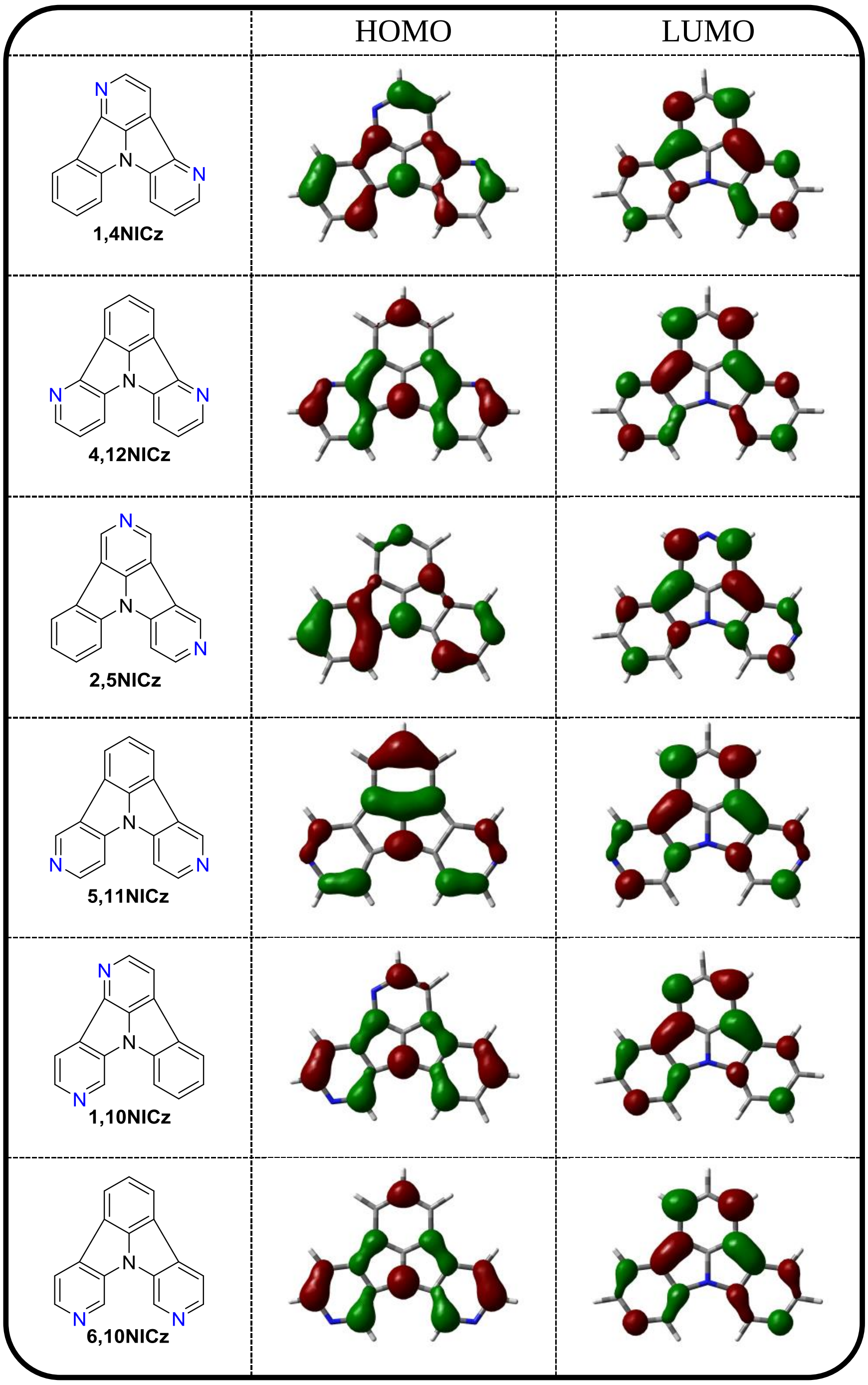


**Figure S75.** Spatial distribution of the HOMO and LUMO levels of twofold substituted NICzs.

# 8 Crystal packing

**Table S2.** Geometries of the C—H···N contacts described in the text. All C--H distances are exactly 0.96 Å since the H atoms were refined asriding on the parent C atoms.

| | H···N [Å] | C···N [Å] | C—H···N [°] |
|---|---|---|---|
| **2NICz** | | | |
| C7—H7···N2 | 2.64 | 3.595(10) | 174.52 |
| C9—H9···N2 | 2.58 | 3.539(10) | 174.62 |
| **5NICz** | | | |
| C7—H7···N5 | 2.49 | 3.438(9) | 168.98 |
| C9—H9···N5 | 2.80 | 3.756(8) | 172.63 |
| C7'—H7'···N5' | 2.48 | 3.411(6) | 162.73 |
| C9'—H9'···N5' | 2.85 | 3.806(7) | 172.47 |
| **2,5NICz** | | | |
| C7—H7···N2 (2×) | 2.59 | 3.525(3) | 165.32 |
| **1,10NICz** | | | |
| C2—H2···N10' | 2.85 | 3.715(6) | 150.62 |
| C9—H9···N1' | 2.68 | 3.565(6) | 153.38 |
| C2'—H2'···N10 | 2.82 | 3.695(6) | 152.58 |
| C9'—H9'···N1 | 2.69 | 3.572(6) | 153.54 |
| **6NICz** | | | |
| C7—H7···N6 | 2.58 | 3.532(3) | 173.39 |
| C9—H9···N6 | 2.54 | 3.498(3) | 175.10 |
| C2'—H2'···N6' | 2.74 | 3.489(3) | 135.06 |
| **6,10NICz** | | | |
| C7—H7···N6 | 2.51 | 3.351(2) | 146.19 |
| C9—H9···N6 | 2.70 | 3.575(2) | 151.42 |

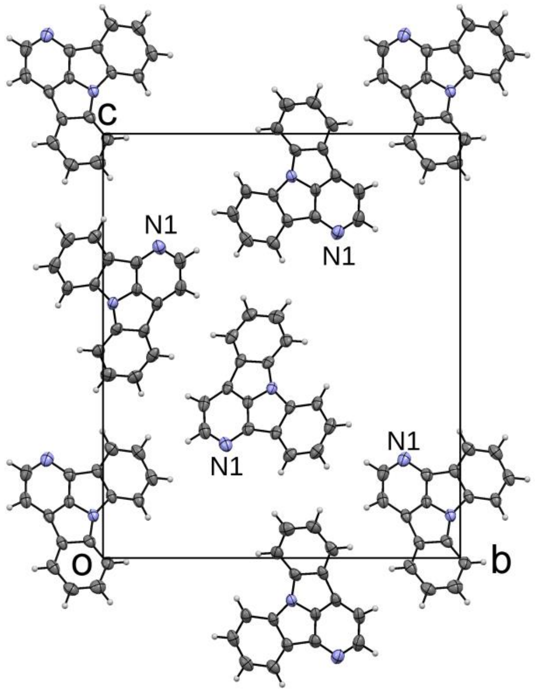


**Figure S76.** Packing of **1NICz**. Color codes as in Figure 6. Disordered solvent molecules have been omitted.

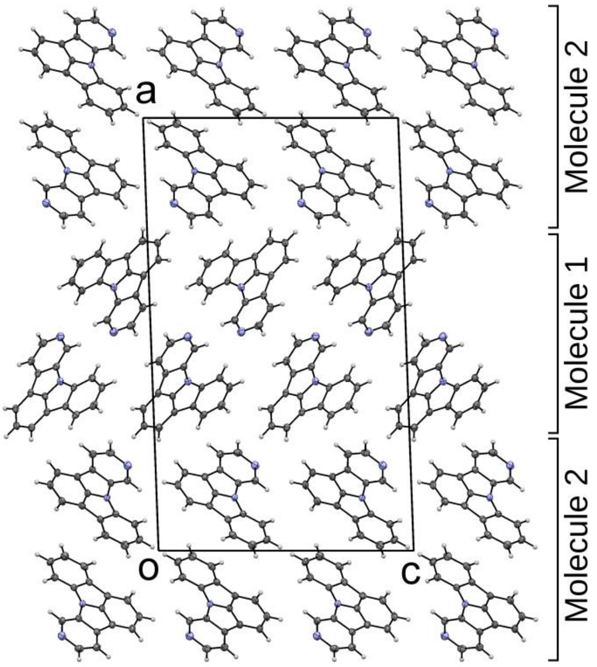


**Figure S77.** Packing of the **6NICz** molecules. Color codes as in Figure 6.

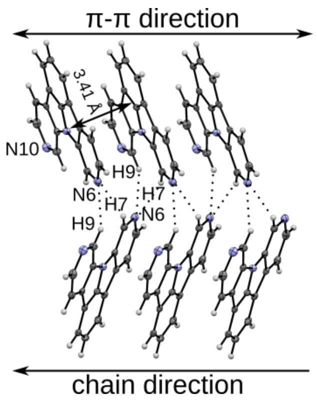


**Figure S78.** Chains of **6,10NICz** connected by hydrogen bonding. Color codes as in Figure 6.

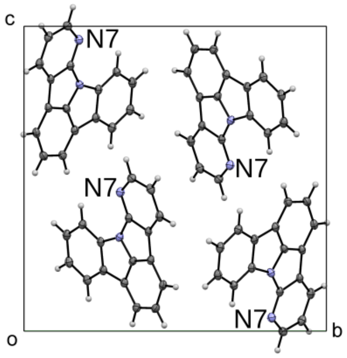


**Figure S79.** Packing of **7NICz**. Color codes as in Figure 6.

# 9 References


(1) Witkop, B. Studies on Carboline Anhydronium Bases. *J. Am. Chem. Soc.* **1953**, *75* (14), 3361–3370.

(2) Kamal, A.; Sathish, M.; Prasanthi, A. V. G.; Chetna, J.; Tangella, Y.; Srinivasulu, V.; Shankaraiah, N.; Alarifi, A. An Efficient One-Pot Decarboxylative Aromatization of Tetrahydro-β-Carbolines by Using N-Chlorosuccinimide: Total Synthesis of Norharmane, Harmane and Eudistomins. *RSC Adv.* **2015**, *5* (109), 90121–90126.

(3) Chen, J.; Chen, W.; Hu, Y. Microwave-Enhanced Fischer Reaction: An Efficient One-Pot Synthesis of Gamma-Carbolines. *Synlett* **2008**, No. 1, 0077–0082.

(4) Iwaki, T.; Yasuhara, A.; Sakamoto, T. Novel Synthetic Strategy of Carbolines via Palladium-Catalyzed Amination and Arylation Reaction. *J. Chem. Soc. Perkin Trans. 1* **1999**, No. 11, 1505–1510.

(5) Chan, K. L.; Watkins, S. E.; Mak, C. S. K.; McKiernan, M. J.; Towns, C. R.; Pascu, S. I.; Holmes, A. B. Poly(9,9-Dialkyl-3,6-Dibenzosilole)—a High Energy Gap Host for Phosphorescent Light Emitting Devices. *Chem. Commun.* **2005**, No. 46, 5766–5768.

(6) Navarro, O.; Nolan, S. P. Large-Scale One-Pot Synthesis of N-Heterocyclic Carbene-Pd(allyl)Cl Complexes. *Synthesis (Stuttg).* **2006**, No. 2, 366–367.
